# Sulforaphane and Broccoli-Derived Preparations in Obesity and Obesity-Related Metabolic Dysfunction: Mechanistic Insights, Preclinical Evidence, and Clinical Perspectives

**DOI:** 10.3390/ph19081244

**Published:** 2026-08-07

**Authors:** Efthymios Poulios, Sousana K. Papadopoulou, Evmorfia Psara, Dimitrios Tasoulas, Constantinos Giaginis

**Affiliations:** 1 Department of Food Science and Nutrition, University of the Aegean, 81400 Lemnos, Greece; epoulios@aegean.gr (E.P.); fnsd21013@fns.aegean.gr (E.P.); 2 Department of Nutritional Sciences and Dietetics, International Hellenic University, 57001 Thessaloniki, Greece; souzpapa@gmail.com; 3 2nd Department of Cardiology, General Hospital of Nikea-Piraeus, 18454 Athens, Greece; dimitriostasoulas@gmail.com

**Keywords:** glucoraphanin, metabolic health, gut microbiota, phytochemicals, bioavailability, precision nutrition, safety, in vitro studies, animal studies, human trials

## Abstract

**Background/Objectives:** Obesity is a major global health challenge characterized by adipose tissue dysfunction, insulin resistance, chronic low-grade inflammation, oxidative stress, mitochondrial dysfunction, and increased cardiometabolic risk. Despite substantial therapeutic advances, limitations related to cost, adverse effects, and long-term adherence have stimulated interest in complementary nutritional approaches. Sulforaphane, a bioactive isothiocyanate derived primarily from broccoli and other cruciferous vegetables, has attracted considerable attention because of its antioxidant, anti-inflammatory, and metabolic regulatory properties. This narrative review critically evaluates the current evidence regarding the role of sulforaphane and broccoli-derived preparations in obesity and obesity-associated metabolic dysfunction. **Methods:** A narrative review was conducted using PubMed/MEDLINE, Scopus, Web of Science, and Google Scholar. Eligible publications included in vitro, animal, clinical, observational, and relevant review studies investigating sulforaphane, glucoraphanin, or broccoli-derived preparations in relation to obesity, adiposity, insulin resistance, metabolic syndrome, inflammation, oxidative stress, energy metabolism, and related metabolic abnormalities. **Results:** In vitro studies consistently demonstrate inhibition of adipocyte differentiation and lipid accumulation, attenuation of oxidative stress and inflammatory signaling, and enhancement of cellular energy metabolism. Animal studies further report reductions in adiposity, insulin resistance, hepatic steatosis, oxidative stress, and chronic inflammation, together with improvements in energy expenditure, metabolic flexibility, and obesity-associated metabolic abnormalities. Mechanistic evidence indicates that sulforaphane exerts pleiotropic metabolic effects through activation of the Nrf2 and AMPK pathways, suppression of NF-κB-mediated inflammation, improvement of mitochondrial function, promotion of thermogenesis and adipose tissue browning, regulation of lipid metabolism, and modulation of gut microbiota composition. Human studies, although limited and heterogeneous, suggest possible improvements in glycemic control, insulin sensitivity, endothelial function, and other surrogate metabolic biomarkers associated with obesity. However, evidence demonstrating clinically meaningful reductions in body weight, adiposity, or body composition remains limited and inconsistent, and improvements in these surrogate biomarkers should not be interpreted as evidence of reduced obesity-related morbidity or clinically meaningful adiposity reduction. Bioavailability, myrosinase activity, food processing, gut microbiota composition, and interindividual variability remain important determinants of efficacy and key translational challenges. **Conclusions:** Current evidence provides strong mechanistic and preclinical support for sulforaphane as a promising candidate adjunctive nutritional intervention for improving obesity-associated metabolic dysfunction. Nevertheless, current human evidence suggests possible metabolic benefits but does not establish sulforaphane as an effective weight-loss intervention or an evidence-based treatment for obesity. Large, long-term randomized controlled trials employing standardized sulforaphane preparations and comprehensive assessments of body weight, adiposity, body composition, metabolic health, pharmacokinetics, and gut microbiota composition are required to establish its clinical efficacy and define its role within precision nutrition strategies for obesity and obesity-associated metabolic dysfunction.

## 1. Introduction

### 1.1. Obesity as a Global Health Challenge

Obesity is one of the most pressing public health challenges worldwide, affecting individuals across all age groups and socioeconomic strata [1]. Defined as abnormal or excessive body fat accumulation that impairs health, obesity has reached epidemic proportions, with more than one billion people estimated to be living with obesity globally. Its prevalence continues to increase owing to the complex interplay of genetic, environmental, behavioral, socioeconomic, and lifestyle factors that collectively disrupt energy homeostasis and metabolic regulation [1].

The burden of obesity extends far beyond excess body weight, imposing substantial healthcare costs while contributing to reduced quality of life, disability, and premature mortality [2]. Obesity is now recognized as a chronic, multifactorial disease characterized not only by excessive adipose tissue accumulation but also by profound metabolic, endocrine, and immunological disturbances that collectively drive disease progression [3]. Dysfunctional expansion of adipose tissue promotes chronic low-grade inflammation, oxidative stress, mitochondrial dysfunction, impaired adipokine secretion, and insulin resistance, thereby contributing to the development of obesity-related metabolic dysfunction and multiple chronic complications [3].

Accordingly, obesity is a major risk factor for numerous non-communicable diseases, including type 2 diabetes mellitus, hypertension, dyslipidemia, cardiovascular disease, metabolic syndrome, Metabolic Dysfunction-Associated Steatotic Liver Disease (MASLD), chronic kidney disease, and obstructive sleep apnea [4]. In addition, obesity has been associated with musculoskeletal disorders, reproductive dysfunction, cognitive impairment, and an increased risk of several malignancies, including colorectal, breast, endometrial, pancreatic, and liver cancers [4]. These diverse clinical consequences underscore the systemic nature of obesity and emphasize that effective management should address not only excess adiposity but also the underlying metabolic abnormalities that contribute to disease progression and long-term complications.

Although significant advances have been made in obesity treatment, current therapeutic strategies remain associated with important limitations. Lifestyle interventions, including dietary modification, increased physical activity, and behavioral therapy, remain the cornerstone of obesity management but are frequently limited by poor long-term adherence and weight regain [5]. Pharmacological therapies, particularly glucagon-like peptide-1 receptor agonists (GLP-1RAs) and newer incretin-based agents, have demonstrated substantial efficacy in promoting weight loss and improving cardiometabolic outcomes; however, their use may be constrained by gastrointestinal adverse effects, high costs, the need for prolonged treatment, and limited accessibility in many healthcare settings [6,7]. Likewise, bariatric surgery remains the most effective intervention for achieving sustained weight loss and remission of obesity-related comorbidities, but its invasive nature, potential perioperative risks, and restricted eligibility limit its widespread application [8]. Consequently, increasing attention has focused on complementary nutritional strategies capable of modulating the biological pathways underlying obesity and obesity-related metabolic dysfunction, with the potential to complement established therapeutic approaches and improve long-term metabolic health.

### 1.2. Nutritional Bioactive Compounds in Obesity and Obesity-Related Metabolic Dysfunction

Growing evidence indicates that dietary bioactive compounds can modulate multiple biological pathways involved in obesity pathophysiology and obesity-related metabolic dysfunction, thereby complementing established therapeutic strategies [9]. Bioactive constituents, including polyphenols, flavonoids, carotenoids, terpenoids, organosulfur compounds, dietary fibers, and probiotics, exert biological activities beyond their nutritional value and have been shown to influence adipogenesis, lipid metabolism, glucose homeostasis, inflammatory signaling, oxidative stress, mitochondrial function, thermogenesis, and gut microbiota composition. Through these pleiotropic actions, dietary bioactive compounds may improve metabolic processes that contribute to obesity and its associated cardiometabolic complications [9].

Among functional foods, cruciferous vegetables have attracted considerable scientific interest owing to their high content of glucosinolates and related bioactive metabolites [10,11]. Members of the Brassicaceae family, including broccoli, cabbage, kale, cauliflower, and Brussels sprouts, are rich sources of sulfur-containing phytochemicals that exhibit antioxidant, anti-inflammatory, anticancer, cardioprotective, and metabolic regulatory properties. These biological activities have been associated with beneficial effects on several molecular pathways implicated in chronic diseases, including obesity-associated metabolic disorders [10,11].

Among the bioactive compounds derived from cruciferous vegetables, sulforaphane is one of the most extensively investigated. This naturally occurring isothiocyanate, generated through the enzymatic hydrolysis of glucoraphanin, has attracted considerable attention because of its ability to modulate oxidative stress, inflammatory signaling, mitochondrial function, insulin sensitivity, and cellular energy metabolism [10,12]. Experimental evidence further suggests that sulforaphane influences key mechanisms involved in obesity pathophysiology, including adipogenesis, lipid metabolism, thermogenesis, and glucose homeostasis. Consequently, sulforaphane has emerged as a promising candidate for improving obesity-related metabolic dysfunction and represents an attractive target for further mechanistic, preclinical, and clinical investigation [10,12].

### 1.3. Sulforaphane and Broccoli-Derived Preparations

Sulforaphane is a naturally occurring isothiocyanate derived primarily from broccoli (*Brassica oleracea* var. *italica*) and broccoli sprouts. It is generated through the enzymatic hydrolysis of its glucosinolate precursor, glucoraphanin, by the enzyme myrosinase following disruption of plant tissues during chewing, cutting, or food processing [10,12].

The efficiency of sulforaphane formation is influenced by numerous factors, including plant genotype, cultivation and environmental conditions, harvest and storage practices, food processing methods, cooking procedures, and gut microbiota activity [13,14]. Thermal processing may substantially reduce myrosinase activity, thereby limiting sulforaphane formation, whereas certain intestinal microorganisms can convert glucoraphanin to sulforaphane in the absence of active plant myrosinase. However, the efficiency of this microbial conversion varies considerably among individuals because of differences in gut microbial composition and glucoraphanin-metabolizing capacity [13,14].

To maximize sulforaphane delivery, various broccoli-derived preparations have been developed for research and nutritional supplementation, including fresh broccoli, broccoli sprouts, broccoli seeds, glucoraphanin-rich extracts, sulforaphane-rich extracts, and dietary supplements [15,16]. Among these, broccoli sprouts represent the richest natural source of glucoraphanin, frequently containing 10- to 100-fold higher concentrations than mature broccoli, depending on cultivar, cultivation conditions, developmental stage, and analytical methodology [15,16].

Bioavailability is a critical determinant of sulforaphane efficacy because it directly influences systemic exposure and the magnitude of its biological effects [16,17]. Following intestinal absorption, sulforaphane undergoes rapid phase II metabolism through the mercapturic acid pathway, initially forming glutathione conjugates that are subsequently converted to cysteinylglycine, cysteine, and N-acetylcysteine metabolites before being excreted predominantly in urine [16,17]. Consequently, substantial interindividual variability in sulforaphane bioavailability has been reported and is influenced by multiple factors, including myrosinase activity, food matrix characteristics, formulation, gastrointestinal physiology, host genetic background, dietary habits, and gut microbiota composition [13,16,17,18].

**Figure 1:**
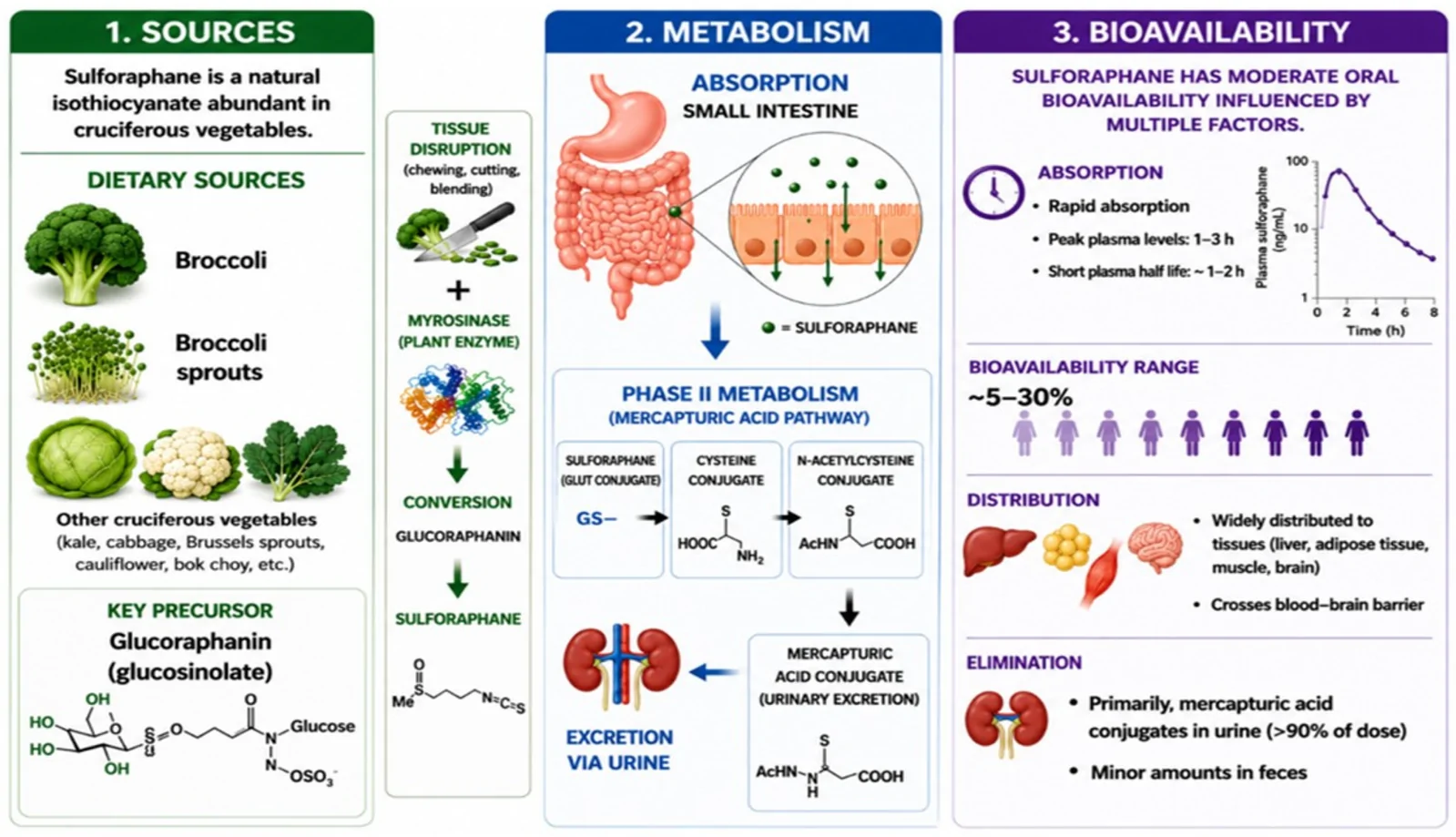
Sources, metabolism, and bioavailability of sulforaphane. The figure was prepared by the authors based on the available evidence summarized in Refs. [10,11,12,13,14,15,16,17,18].

The presence of active myrosinase has consistently been shown to enhance sulforaphane bioavailability, whereas reliance on microbial glucoraphanin hydrolysis alone generally results in lower and more variable systemic exposure [13,16,17,18]. Accordingly, differences in sulforaphane absorption, metabolism, and excretion represent important sources of variability among experimental and clinical studies and should be carefully considered when interpreting biological responses and translational efficacy. Figure 1 summarizes the principal dietary sources, metabolic conversion, and bioavailability of sulforaphane.

Beyond its distinctive pharmacokinetic characteristics and complex metabolism, extensive experimental evidence indicates that sulforaphane modulates multiple biological pathways implicated in obesity pathophysiology and obesity-associated metabolic dysfunction. Sulforaphane is a potent activator of the nuclear factor erythroid 2-related factor 2 (Nrf2) signaling pathway and attenuates oxidative stress and chronic inflammatory responses that contribute to adipose tissue dysfunction and metabolic impairment [19,20]. In addition, experimental studies have demonstrated that sulforaphane influences adipogenesis, lipid metabolism, thermogenesis, white adipose tissue browning, insulin signaling, and glucose homeostasis while improving mitochondrial function and cellular energy metabolism [19,20]. Collectively, these findings provide strong mechanistic evidence supporting the biological plausibility that sulforaphane may modulate key pathways involved in obesity and obesity-associated metabolic dysfunction. However, although experimental evidence consistently demonstrates beneficial effects on these biological mechanisms, current clinical evidence remains limited and heterogeneous and has not consistently demonstrated clinically meaningful reductions in body weight, adiposity, or body composition. These translational limitations are critically examined in the following sections.

### 1.4. Aim and Scope of the Review

Given the increasing global prevalence of obesity and the limitations of current therapeutic strategies, there is growing interest in identifying potentially safe and effective nutritional interventions capable of targeting the complex biological mechanisms underlying obesity and its associated metabolic dysfunction. Sulforaphane and broccoli-derived preparations have emerged as promising candidates for modulating biological pathways involved in obesity-associated metabolic dysfunction, including oxidative stress, chronic inflammation, adipogenesis, lipid metabolism, mitochondrial dysfunction, thermogenesis, and insulin resistance.

Although numerous review articles have summarized the antioxidant, anti-inflammatory, anticancer, and general metabolic properties of sulforaphane and broccoli-derived preparations, relatively few have specifically examined their potential role within the context of obesity while integrating mechanistic, preclinical, clinical, pharmacokinetic, microbiome, and translational evidence. Furthermore, studies investigating obesity-related metabolic abnormalities, such as insulin resistance, hepatic steatosis, oxidative stress, mitochondrial dysfunction, endothelial dysfunction, and chronic inflammation, are frequently considered separately despite representing fundamental components of obesity pathophysiology. Consequently, comprehensive narrative reviews critically integrating the available evidence across these complementary research areas remain limited.

Accordingly, the present narrative review provides a comprehensive and critical synthesis of the current evidence regarding the role of sulforaphane and broccoli-derived preparations in obesity and obesity-related metabolic dysfunction. Specifically, the review evaluates findings from in vitro and in vivo studies investigating the molecular and cellular mechanisms through which sulforaphane influences biological pathways relevant to obesity, including adipogenesis, lipid metabolism, thermogenesis, mitochondrial function, oxidative stress, inflammation, insulin signaling, and glucose homeostasis. In addition, it critically examines evidence from human clinical studies assessing the effects of sulforaphane and broccoli-derived preparations on obesity-related metabolic outcomes while distinguishing studies directly evaluating obesity-related endpoints, such as body weight, adiposity, body composition, and energy balance, from mechanistic investigations that provide supportive evidence through improvements in metabolic, inflammatory, oxidative, or mitochondrial abnormalities closely associated with obesity.

Particular emphasis is placed on integrating evidence across mechanistic, preclinical, and clinical investigations to provide a balanced assessment of the biological plausibility, translational relevance, and current limitations of sulforaphane as a complementary nutritional strategy for obesity-associated metabolic dysfunction. The review also discusses factors influencing bioavailability, pharmacokinetics, gut microbiota interactions, and formulation-dependent efficacy, while highlighting current knowledge gaps and future research priorities required to facilitate the translation of sulforaphane-based interventions into evidence-based nutritional strategies.

To the best of our knowledge, this is among the few narrative reviews that holistically integrate mechanistic, preclinical, clinical, microbiome-related, pharmacokinetic, and bioavailability evidence concerning sulforaphane and broccoli-derived preparations within the broader framework of obesity and obesity-related metabolic dysfunction. By critically evaluating both direct evidence from obesity models and supportive mechanistic evidence from obesity-associated metabolic disorders, this review aims to provide a comprehensive and balanced perspective on the therapeutic potential and translational challenges of sulforaphane in this rapidly evolving field.

## 2. Evidence from In Vitro Cellular Studies

In vitro studies have provided a substantial body of mechanistic evidence supporting the potential role of sulforaphane and broccoli-derived preparations in modulating biological pathways involved in obesity and obesity-related metabolic dysfunction. As summarized in Table 1, available cellular studies comprise both direct obesity-related models, primarily investigating adipogenesis, adipocyte differentiation, lipid accumulation, thermogenesis, and adipocyte browning, as well as supportive mechanistic models examining obesity-associated metabolic abnormalities, including insulin resistance, hepatic steatosis, inflammation, oxidative stress, mitochondrial dysfunction, and impaired cellular energy metabolism [21,22,23,24,25,26,27,28,29,30,31,32,33,34,35,36,37,38,39,40,41,42,43,44,45,46].

Most investigations have employed murine 3T3-L1 preadipocytes, one of the most widely accepted experimental models for studying adipogenesis and adipocyte biology [21,22,23,25,26,27,38,39,40]. Additional studies have utilized primary human adipocytes, hepatocytes, skeletal muscle cells, macrophages, endothelial cells, pancreatic β-cells, cardiomyocytes, retinal cells, and co-culture systems to investigate the effects of sulforaphane on molecular pathways relevant to obesity pathophysiology and its associated metabolic complications [24,28,29,30,31,32,33,34,35,36,37,41,42,43,44,45,46]. Although these complementary cellular models do not directly evaluate obesity-related endpoints such as body weight or adiposity, they provide important mechanistic insight into biological processes that contribute to obesity-associated metabolic dysfunction. Collectively, these studies demonstrate that sulforaphane exerts pleiotropic biological activities capable of modulating multiple interconnected pathways implicated in obesity and its metabolic sequelae.

Unlike many conventional anti-obesity agents that primarily target appetite regulation or nutrient absorption, sulforaphane appears to influence a broad network of signaling pathways, including Nrf2, AMP-activated protein kinase (AMPK), nuclear factor-κB (NF-κB), phosphatidylinositol-3-kinase (PI3K)/protein kinase B (Akt), mammalian target of rapamycin (mTOR), autophagy-related pathways, mitochondrial quality control mechanisms, and inflammatory signaling cascades [21,22,23,24,25,26,27,28,29,30,31,32,33,34,35,36,37,38,39,40,41,42,43,44,45,46]. Through modulation of these interconnected pathways, sulforaphane has attracted considerable interest as a potential metabolic regulator capable of influencing adipocyte differentiation, lipid metabolism, inflammatory responses, oxidative stress, insulin signaling, mitochondrial function, and thermogenic activity. Nevertheless, despite the consistency of many in vitro findings, these observations represent mechanistic evidence generated under controlled experimental conditions. Accordingly, several methodological limitations should be considered when interpreting these results and translating them to the complex pathophysiology of human obesity and obesity-related metabolic dysfunction.

### 2.1. Effects on Adipocyte Differentiation

The differentiation of preadipocytes into mature adipocytes is a fundamental process in adipose tissue development, remodeling, and expansion [47,48]. During adipogenesis, adipose progenitor cells undergo coordinated morphological, metabolic, and transcriptional changes that culminate in the formation of mature lipid-filled adipocytes capable of storing excess energy [47,48]. Under conditions of chronic energy surplus, dysregulated adipogenesis contributes to adipose tissue remodeling and obesity-associated metabolic dysfunction. Consequently, modulation of adipogenic pathways has emerged as a potential therapeutic strategy for improving adipose tissue function and limiting pathological adipose tissue expansion [47,48].

Among the available in vitro evidence, studies employing predominantly murine 3T3-L1 preadipocytes consistently demonstrate that sulforaphane suppresses adipocyte differentiation [21,22,23,24,25,38]. Treatment during the early differentiation phase reduces the formation of mature adipocytes and decreases intracellular lipid droplet accumulation [21,22,23,24,25,28,38]. These anti-adipogenic effects have been observed across multiple experimental conditions and generally occur in a dose-dependent manner [21,22,23,24,25,29,39]. In addition, studies using primary human adipocytes and adipose-derived stem cells have reported comparable inhibitory effects on adipogenic differentiation, suggesting that these observations are not restricted to murine cellular models [23,24]. Nevertheless, it should be noted that only a limited number of studies have been performed in human cells, and further validation in human adipose tissue models remains necessary.

It should also be noted that two studies by Chen et al. and Yang et al. primarily investigated sulforaphene while also including sulforaphane and other structurally related isothiocyanates [49,50]. Their findings suggest that sulforaphene and related isothiocyanates also possess anti-adipogenic activity, with sulforaphene exhibiting greater potency than sulforaphane under the specific experimental conditions investigated [49,50]. However, direct comparisons remain limited, and additional comparative studies are required before differences in biological efficacy among individual isothiocyanates can be established.

Several complementary mechanisms appear to underlie the inhibitory effects of sulforaphane on adipocyte differentiation. One of the earliest events involves suppression of mitotic clonal expansion, a prerequisite for terminal adipocyte differentiation [21,22]. Sulforaphane induces G0/G1 cell-cycle arrest through downregulation of cyclin D1, cyclin A, cyclin-dependent kinase 2 (CDK2), CDK4, and phosphorylated retinoblastoma protein (p-Rb), together with upregulation of the cell-cycle inhibitors p21 and p27 [21,22]. Concurrent activation of AMPK and inhibition of PI3K/Akt and extracellular signal-regulated kinase 1/2 (ERK1/2) signaling further restrict preadipocyte proliferation and interfere with the transcriptional program required for adipocyte maturation [21,22].

Additional studies indicate that sulforaphane regulates multiple signaling pathways involved in adipogenesis beyond cell-cycle control. Activation of AMPK, suppression of adipogenic transcription factors, attenuation of oxidative stress, and modulation of nutrient-sensing pathways collectively contribute to reduced adipocyte differentiation and lipid accumulation [21,22,25,26,27]. In differentiated 3T3-L1 adipocytes, sulforaphane has also been shown to activate the AMPK–mTOR–Unc-51-like autophagy-activating kinase 1 (ULK1) signaling axis, resulting in inhibition of mammalian target of rapamycin (mTOR) activity, activation of lipophagy, and enhanced lipid droplet degradation [26]. Since mTOR is a central regulator of cellular growth, nutrient sensing, and adipocyte differentiation, these findings suggest that modulation of the AMPK–mTOR–ULK1 pathway may contribute to both the anti-adipogenic and lipid-lowering effects of sulforaphane.

Comparable anti-adipogenic effects have also been reported for broccoli-derived preparations rich in glucoraphanin and sulforaphane, including broccoli leaf extracts and broccoli sprout-based formulations [38,39,40,51]. These preparations suppress adipocyte differentiation and lipid accumulation to an extent generally comparable with purified sulforaphane, supporting the concept that sulforaphane is a principal contributor to the metabolic effects of broccoli-derived products. Nevertheless, these preparations contain numerous additional phytochemicals, including other glucosinolates, phenolic compounds, flavonoids, vitamins, minerals, and other bioactive metabolites that may also contribute to the observed biological responses. Consequently, additive or synergistic interactions among multiple constituents cannot be excluded.

Despite the consistency of these findings, several important limitations should be considered. Most available studies rely on the murine 3T3-L1 preadipocyte cell line, which, although widely accepted for mechanistic investigations, cannot fully reproduce the cellular complexity of human adipose tissue. Human adipose tissue comprises multiple interacting cell populations—including adipocytes, immune cells, endothelial cells, fibroblasts, and adipose-derived stem cells—that collectively regulate adipose tissue remodeling through endocrine, paracrine, and immunometabolic interactions. Therefore, cellular responses observed under simplified in vitro conditions should be interpreted as mechanistic evidence rather than direct evidence of clinical efficacy.

Furthermore, although inhibition of adipocyte differentiation is generally considered beneficial in experimental obesity models, complete or sustained suppression of adipogenesis may not always be advantageous. Physiological adipogenesis is essential for healthy adipose tissue expansion and maintenance of metabolic homeostasis, whereas inadequate adipose tissue expandability may promote ectopic lipid deposition in the liver, skeletal muscle, and other peripheral tissues. Consequently, the long-term metabolic consequences of sustained adipogenesis inhibition remain incompletely understood, highlighting the need for additional in vivo and human studies to determine whether modulation, rather than complete suppression, of adipogenesis represents the most favorable therapeutic strategy.

### 2.2. Regulation of Lipid Accumulation

Excessive intracellular lipid accumulation is a characteristic feature of adipocyte dysfunction and contributes to the development of obesity and its associated metabolic complications. Studies performed in adipocyte models consistently demonstrate that sulforaphane reduces triglyceride accumulation during both adipocyte differentiation and in mature adipocytes [21,22,25,26,38,39,40]. These findings have been confirmed using complementary experimental approaches, including Oil Red O staining, triglyceride quantification assays, and microscopic evaluation of intracellular lipid droplets [21,22,25,26,38,39,40].

The reduction in lipid accumulation appears to result from coordinated modulation of multiple metabolic pathways regulating lipid synthesis, storage, and utilization. Sulforaphane suppresses lipogenesis while simultaneously promoting lipid utilization and fatty acid oxidation, with activation of AMPK representing a central regulatory mechanism [21,22,25,26,38,39,40]. During adipogenic differentiation, sulforaphane inhibits lipid droplet formation and intracellular triglyceride deposition through downregulation of key adipogenic transcription factors, including peroxisome proliferator-activated receptor gamma (PPARγ) and CCAAT/enhancer-binding protein alpha (C/EBPα), thereby limiting adipocyte maturation and lipid storage [21,22,25,26,38,39,40].

In mature adipocytes, sulforaphane further reduces intracellular lipid storage by promoting lipid droplet degradation through activation of AMPK-dependent lipophagy pathways [26]. Current evidence indicates that activation of the AMPK–mTOR–ULK1 signaling axis enhances autophagic degradation of lipid droplets and facilitates fatty acid mobilization [26]. However, direct mechanistic evidence supporting activation of this pathway is derived primarily from the study by Masuda et al., whereas Men et al. focused predominantly on adipocyte differentiation and lipid accumulation rather than direct assessment of lipophagic activity [26,39]. Comparable reductions in triglyceride accumulation have also been observed with sulforaphane-rich broccoli-derived preparations, supporting the concept that sulforaphane is a major contributor to the anti-adipogenic activity of cruciferous vegetable preparations [38,39,40,51].

Autophagy has emerged as an additional mechanism contributing to the regulation of intracellular lipid homeostasis [52]. By facilitating degradation of lipid droplets and damaged cellular components, autophagy improves metabolic efficiency and limits intracellular lipid overload [52]. Sulforaphane has been shown to stimulate autophagic flux through activation of AMPK and inhibition of mTOR signaling. In differentiated 3T3-L1 adipocytes, sulforaphane increases AMPK phosphorylation, decreases mTOR phosphorylation, and activates ULK1, thereby promoting lipophagy and autophagic degradation of lipid droplets [26]. Similar AMPK-dependent induction of autophagic flux has also been reported in other experimental systems, suggesting that regulation of the AMPK–mTOR–ULK1 pathway represents a broader biological effect of sulforaphane rather than an adipocyte-specific mechanism [53,54].

Although hepatocyte models do not directly evaluate obesity-related endpoints, hepatic steatosis represents one of the principal metabolic complications of obesity and metabolic dysfunction. Consequently, studies performed in steatotic hepatocytes provide supportive mechanistic evidence regarding the effects of sulforaphane on obesity-associated disturbances in hepatic lipid metabolism rather than direct evidence of anti-obesity efficacy. Several studies have demonstrated that sulforaphane reduces intracellular lipid accumulation and attenuates molecular features characteristic of hepatic steatosis in HepG2, LO2, and other hepatocyte models [33,34,36,41]. Sulforaphane decreases lipid droplet formation and triglyceride accumulation while suppressing major lipogenic regulators, including sterol regulatory element-binding protein-1c (SREBP-1c), fatty acid synthase (FAS), diacylglycerol O-acyltransferase 1 (DGAT1), and PPARγ [33,34,36,41]. These effects are accompanied by activation of AMPK signaling, enhancement of Nrf2-mediated antioxidant defenses, attenuation of endoplasmic reticulum (ER) stress, suppression of inflammatory signaling pathways, and stimulation of autophagy-mediated lipid droplet degradation, collectively improving hepatic lipid homeostasis [32,33,34,36,41]. Furthermore, sulforaphane attenuated palmitic acid-induced apoptosis by reducing cleaved caspase-3 expression, ceramide accumulation, and reactive oxygen species (ROS) generation in hepatocytes [33]. Collectively, these findings suggest that sulforaphane may beneficially modulate molecular pathways involved in obesity-associated metabolic dysfunction beyond adipose tissue, particularly those contributing to MASLD.

Despite the consistency of these mechanistic observations, several limitations should be acknowledged. Most available studies evaluate lipid metabolism under highly controlled experimental conditions that cannot fully reproduce the complex hormonal, nutritional, inflammatory, and multicellular environment characteristic of human obesity. Furthermore, the concentrations of sulforaphane used in some cellular studies may exceed those achievable in human tissues following dietary intake or supplementation. Consequently, these findings should be interpreted as mechanistic evidence supporting the biological plausibility of sulforaphane rather than direct evidence of clinical efficacy, underscoring the need for validation in animal models and well-designed human intervention studies.

### 2.3. Effects on Adipogenic Transcription Factors

The inhibitory effects of sulforaphane on adipocyte differentiation are mediated largely through modulation of transcription factors that regulate adipogenesis, lipid metabolism, and cellular energy homeostasis. Among these, PPARγ and CCAAT/C/EBPα are recognized as the principal transcriptional regulators governing adipocyte differentiation, whereas sterol regulatory element-binding protein-1c (SREBP-1c) primarily regulates lipogenesis and fatty acid synthesis [48,55].

PPARγ is widely regarded as the master regulator of adipogenesis because it coordinates adipocyte differentiation, lipid storage, and the expression of numerous adipocyte-specific genes. Multiple studies performed in differentiating 3T3-L1 adipocytes have consistently demonstrated that sulforaphane suppresses PPARγ expression, resulting in reduced adipocyte maturation and diminished intracellular lipid accumulation [21,22,25,38]. Since activation of PPARγ is required for terminal adipocyte differentiation, its inhibition represents one of the principal mechanisms underlying the anti-adipogenic effects of sulforaphane.

C/EBPα functions cooperatively with PPARγ to establish and maintain the adipogenic transcriptional program. Experimental studies consistently demonstrate that sulforaphane downregulates C/EBPα expression during adipocyte differentiation [21,22,25,38,39]. Simultaneous suppression of PPARγ and C/EBPα disrupts the transcriptional cascade required for terminal adipocyte maturation, thereby limiting adipocyte differentiation and intracellular lipid accumulation [55]. Collectively, these findings indicate that coordinated inhibition of these two transcription factors constitutes a central molecular mechanism through which sulforaphane modulates adipogenesis in cellular models.

In addition to its effects on adipocyte differentiation, sulforaphane regulates lipogenic transcriptional networks involved in hepatic lipid metabolism. Although these observations originate from hepatocyte models and therefore represent supportive mechanistic evidence rather than direct evidence of anti-obesity activity, several studies have demonstrated that sulforaphane suppresses SREBP-1c together with downstream lipogenic enzymes, including FAS and acetyl-CoA carboxylase (ACC), thereby reducing de novo lipogenesis and intracellular lipid accumulation in cellular models of hepatic steatosis [33,35,37]. These findings suggest that modulation of lipogenic transcriptional programs may contribute to the beneficial effects of sulforaphane on obesity-associated metabolic dysfunction, particularly MASLD.

Beyond transcriptional regulation, emerging evidence suggests that sulforaphane may also influence metabolic homeostasis through epigenetic mechanisms. One mechanistic study demonstrated that sulforaphane inhibits histone deacetylase 8 (HDAC8), thereby enhancing peroxisome proliferator-activated receptor gamma coactivator-1 alpha (PGC-1α)-mediated mitochondrial biogenesis and oxidative metabolism in skeletal muscle cells [31]. Mechanistically, sulforaphane promoted cAMP response element-binding protein (CREB) phosphorylation and p53 acetylation, leading to activation of the HDAC8–PGC-1α signaling pathway. These molecular effects were accompanied by improvements in energy expenditure and attenuation of metabolic dysfunction in high-fat diet-fed mice [31]. More broadly, sulforaphane has been proposed to function as an epigenetic modulator through inhibition of histone deacetylases, with potential downstream effects on chromatin remodeling, DNA methylation, and microRNA expression. However, the available evidence remains limited and derives primarily from isolated mechanistic investigations rather than comprehensive obesity-specific epigenetic studies. Consequently, the contribution of these epigenetic mechanisms to the long-term metabolic effects of sulforaphane requires further investigation.

Importantly, although downregulation of adipogenic transcription factors is generally considered beneficial in experimental models of obesity, these regulatory proteins also play essential physiological roles in maintaining adipocyte function, adipose tissue homeostasis, and insulin sensitivity [48,55]. Therefore, complete or sustained suppression of these pathways may not necessarily be desirable. Instead, the available evidence suggests that modulation, rather than complete inhibition, of adipogenic transcriptional networks may represent the most appropriate strategy for improving adipose tissue function while preserving healthy adipose tissue remodeling.

### 2.4. Effects on Inflammatory and Oxidative Stress Markers

Chronic low-grade inflammation and oxidative stress are hallmarks of obesity-associated metabolic dysfunction and play central roles in the development of adipose tissue dysfunction, insulin resistance, and obesity-related complications [56,57]. Excess nutrient availability promotes excessive production of ROS, activation of inflammatory signaling pathways, and secretion of pro-inflammatory cytokines, thereby disrupting adipocyte function and metabolic homeostasis [56,57]. Consequently, modulation of inflammatory and oxidative stress pathways has emerged as an important therapeutic target for obesity and its associated metabolic disorders.

Experimental studies performed in adipocytes, monocytes, macrophages, hepatocytes, and other metabolically stressed cellular models have consistently demonstrated that sulforaphane attenuates inflammatory responses through multiple complementary mechanisms [27,29,46]. Sulforaphane reduces the production of major pro-inflammatory mediators, including tumor necrosis factor-α (TNF-α), interleukin-6 (IL-6), IL-1β, and NOD-, LRR-, and pyrin domain-containing protein 3 (NLRP3) inflammasome-associated factors, while simultaneously promoting macrophage polarization toward an anti-inflammatory M2 phenotype [27,29]. These effects are mediated predominantly through inhibition of NF-κB signaling and downstream inflammatory gene expression, thereby attenuating the inflammatory crosstalk between adipocytes and immune cells that contributes to obesity-associated adipose tissue dysfunction and insulin resistance.

In parallel with its anti-inflammatory activity, sulforaphane is a potent activator of the Nrf2/antioxidant response element (ARE) signaling pathway, which represents the principal endogenous defense mechanism against oxidative stress. In metabolically stressed adipocytes, macrophages, hepatocytes, and other cellular models, sulforaphane promotes nuclear translocation of Nrf2 and subsequent transcriptional activation of multiple antioxidant and cytoprotective genes [27,45]. These include heme oxygenase-1 (HO-1), NAD(P)H:quinone oxidoreductase-1 (NQO1), superoxide dismutase (SOD), glutathione peroxidase (GPx), glutamate-cysteine ligase catalytic and modifier subunits (GCLC/GCLM), and other glutathione-related enzymes, resulting in reduced intracellular ROS production, attenuation of oxidative stress, preservation of cellular integrity, and improvement of metabolic homeostasis [27,45].

Activation of the NLRP3 inflammasome has emerged as a critical mediator linking obesity-induced oxidative stress to chronic inflammation and insulin resistance [58,59]. Upon activation, the NLRP3 complex promotes caspase-1-mediated maturation and secretion of IL-1β and IL-18, thereby amplifying inflammatory responses within adipose tissue and other metabolically active organs [58,59]. Although most available evidence derives from macrophages, hepatocytes, and other metabolically stressed cellular systems rather than adipocytes, these studies provide important supportive mechanistic evidence regarding the regulation of obesity-associated inflammation. Sulforaphane consistently suppresses NLRP3 inflammasome activation, reduces caspase-1 activity and IL-1β production, and attenuates inflammatory signaling in palmitic acid-stimulated monocytes, adipose tissue macrophages, saturated fatty acid-treated hepatocytes, and retinal epithelial cells [29,46,58,59]. These effects are associated with reduced ROS production, restoration of mitochondrial function, activation of autophagic and mitophagic pathways, inhibition of inflammatory gene expression, and enhancement of antioxidant defenses [29,46,58,59]. While activation of Nrf2 contributes substantially to these anti-inflammatory and antioxidant effects, experimental evidence also suggests that sulforaphane may inhibit NLRP3 inflammasome activation through partially Nrf2-independent mechanisms [60].

Additional mechanistic studies indicate that sulforaphane modulates endoplasmic reticulum (ER) stress, another important contributor to obesity-associated metabolic dysfunction. In metabolically stressed hepatocytes, sulforaphane reduces the expression of ER stress markers, including glucose-regulated protein 78 (GRP78), protein kinase RNA (PKR)-like ER kinase (PERK), and C/EBP homologous protein (CHOP), while improving ER–mitochondria communication and hepatic glucose metabolism [42,43]. Similar cytoprotective effects have been reported in hypoxia/reoxygenation-injured cardiomyocytes, where sulforaphane preserves mitochondrial membrane potential, suppresses ER stress-associated apoptotic proteins (GRP78, CHOP, and caspase-12), increases silent information regulator 1 (SIRT1) expression, and improves the Bcl-2/Bax ratio [44]. Although these studies were not conducted in obesity-specific cellular models, they provide additional mechanistic evidence supporting the cytoprotective actions of sulforaphane against oxidative stress, mitochondrial dysfunction, and ER stress, all of which contribute to obesity-associated metabolic abnormalities.

Despite the consistency of these findings, several limitations should be considered. Most studies employ simplified in vitro systems using artificial inflammatory or oxidative stress stimuli, including lipopolysaccharide (LPS), palmitic acid, hydrogen peroxide, or hypoxia/reoxygenation, which cannot fully reproduce the chronic, multifactorial inflammatory environment characteristic of human obesity. Furthermore, the relative contribution of individual signaling pathways, including NF-κB, Nrf2, NLRP3, and ER stress pathways, may differ substantially among cell types and experimental conditions. Consequently, these observations should be interpreted as mechanistic evidence supporting the biological plausibility of sulforaphane rather than direct evidence of clinical efficacy, emphasizing the need for confirmation in animal models and well-designed human intervention studies.

### 2.5. Effects on Thermogenic and Mitochondrial Genes

Enhancing energy expenditure through adaptive thermogenesis has emerged as an attractive strategy for improving metabolic health and limiting obesity-associated metabolic dysfunction. Consequently, considerable interest has focused on the ability of sulforaphane to modulate thermogenic programming, mitochondrial biogenesis, and cellular energy metabolism. Most available evidence originates from adipocyte and skeletal muscle cell models, providing mechanistic insight into pathways involved in energy homeostasis rather than direct evidence of increased whole-body energy expenditure.

Experimental studies performed in adipocyte models have demonstrated that sulforaphane promotes a thermogenic molecular phenotype characterized by increased expression of uncoupling protein-1 (UCP1), PGC-1α, and multiple regulators of mitochondrial biogenesis [27,28]. In beige adipocyte and adipocyte browning models, these molecular changes are accompanied by enhanced mitochondrial content, increased fatty acid oxidation, improved glucose utilization, and elevated expression of thermogenic genes, findings consistent with activation of a brown-like adipocyte program [27,28]. Sulforaphane has also been reported to increase the expression of PR domain-containing protein 16 (PRDM16), although the available evidence supporting regulation of PRDM16 is currently less extensive than that for UCP1, PGC-1α, and other markers of mitochondrial biogenesis [28].

Beyond adipocytes, several mechanistic studies performed in hepatocytes and skeletal muscle cells provide supportive mechanistic evidence that sulforaphane enhances mitochondrial function and bioenergetic capacity [30,31]. Sulforaphane increases mitochondrial biogenesis through upregulation of PGC-1α, nuclear respiratory factors 1 and 2 (NRF-1 and NRF-2), and mitochondrial transcription factor A (TFAM), resulting in increased mitochondrial content, elevated mitochondrial DNA copy number, enhanced respiratory chain activity, and improved fatty acid oxidation [27,28,30]. In addition, selected studies have demonstrated increased mitochondrial oxygen consumption, oxidative phosphorylation, and ATP production, indicating improved mitochondrial respiratory efficiency and cellular energy metabolism [27,30,31]. Collectively, these findings suggest that sulforaphane improves mitochondrial function in metabolically active tissues, thereby supporting metabolic homeostasis and insulin sensitivity.

Maintenance of mitochondrial quality is equally important for preserving cellular metabolic function. In addition to stimulating mitochondrial biogenesis, accumulating evidence suggests that sulforaphane may improve mitochondrial quality control through activation of mitophagy and related mitochondrial surveillance pathways [27,30,31]. By facilitating the selective removal of dysfunctional mitochondria, mitophagy may reduce mitochondrial ROS production, improve respiratory efficiency, and preserve cellular bioenergetics. However, direct evidence demonstrating activation of mitophagy by sulforaphane in adipocytes remains limited. Most mechanistic support currently derives from broader cellular studies investigating mitochondrial quality control rather than adipocyte-specific experiments [61,62,63].

Sulforaphane has also been shown to improve cellular glucose utilization and insulin responsiveness in metabolically stressed adipocytes, hepatocytes, and skeletal muscle cells [27,32,45,64,65]. These effects are associated with restoration of insulin receptor substrate-1 (IRS-1)/Akt signaling and, particularly in skeletal muscle cells, enhanced glucose transporter type 4 (GLUT4) translocation to the plasma membrane [27,32,64,65]. Although activation or restoration of Akt-dependent insulin signaling has been consistently demonstrated, direct evaluation of the complete PI3K/Akt signaling cascade has not been performed in all experimental models [27,32,45]. Instead, current evidence suggests that coordinated crosstalk among PI3K/Akt, AMPK, Nrf2, NF-κB, and mTOR signaling pathways collectively mediates many of the observed metabolic adaptations.

Despite the consistent upregulation of thermogenic and mitochondrial markers reported across multiple studies, several important limitations should be acknowledged. Most investigations infer thermogenic activation primarily from increased expression of molecular markers, including UCP1, PGC-1α, PRDM16, NRFs, and TFAM, rather than from direct assessment of thermogenic function. Only a limited number of studies have evaluated mitochondrial respiration, oxygen consumption rate, or ATP production, while direct measurements of heat production or cellular energy expenditure remain scarce. Consequently, increased expression of thermogenic genes should not be interpreted as definitive evidence of enhanced thermogenesis or increased energy expenditure [63]. Likewise, although mitochondrial adaptations are likely to contribute to the favorable metabolic effects of sulforaphane, their quantitative contribution to obesity prevention or treatment remains uncertain. Therefore, the available findings should be interpreted as mechanistic evidence supporting the biological plausibility of sulforaphane as a modulator of mitochondrial function and thermogenic programming, warranting further validation through comprehensive functional metabolic studies and well-designed in vivo and clinical investigations.

### 2.6. Critical Appraisal of the In Vitro Evidence and Translational Perspectives

Collectively, the available in vitro evidence provides substantial mechanistic support for the potential role of sulforaphane and broccoli-derived preparations in modulating biological pathways involved in obesity and obesity-associated metabolic dysfunction. Across both direct obesity-related cellular models, primarily investigating adipogenesis, lipid accumulation, adipocyte browning, and thermogenic programming, and supportive mechanistic models examining inflammation, insulin resistance, hepatic steatosis, oxidative stress, mitochondrial dysfunction, and cellular energy metabolism, sulforaphane consistently demonstrates pleiotropic biological activities. These include suppression of adipocyte differentiation, reduction in intracellular lipid accumulation, attenuation of inflammatory and oxidative stress responses, enhancement of mitochondrial function and quality control, promotion of thermogenic gene expression, and improvement of cellular metabolic homeostasis. Collectively, these effects are mediated through coordinated modulation of multiple interconnected signaling pathways implicated in obesity pathophysiology, including Nrf2, AMPK, NF-κB, PI3K/Akt, mTOR, autophagy-related pathways, and regulators of mitochondrial quality control. Taken together, these observations support the concept that sulforaphane functions as a multi-target metabolic regulator capable of influencing several complementary processes involved in adipose tissue biology and metabolic homeostasis.

**Figure 2:**
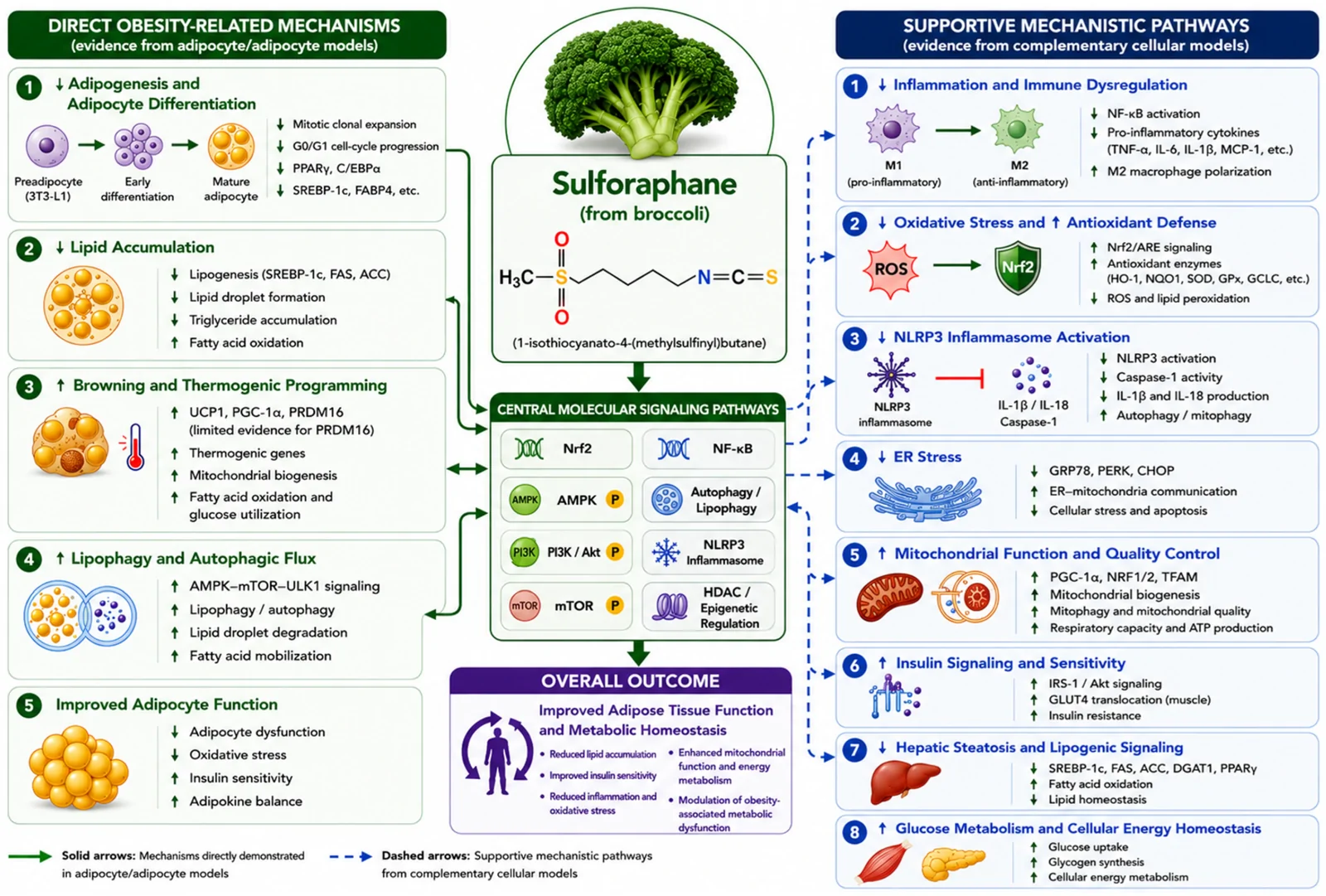
Molecular mechanisms through which sulforaphane modulates obesity and obesity-associated metabolic dysfunction. The figure was created by the authors based on the mechanistic evidence summarized in Refs. [19,20,21,22,23,24,25,26,27,28,29,30,31,32,33,34,35,36,37,38,39,40,41,42,43,44,45,46], with complementary support from the preclinical studies discussed in Refs. [47,48,49,50,51,52,53,54,55,56,57,58,59,60,61,62,63,64,65,66,67,68,69,70,71,72,73,74].

Importantly, the biological actions of sulforaphane extend beyond regulation of adipocyte differentiation. Experimental cellular studies indicate that sulforaphane enhances fatty acid oxidation, stimulates autophagy and lipophagy, attenuates endoplasmic reticulum stress, suppresses NLRP3 inflammasome activation, improves mitochondrial quality control, and may influence epigenetic regulatory mechanisms. These observations are particularly relevant given the multifactorial nature of obesity, which involves complex interactions among adipose tissue dysfunction, chronic inflammation, oxidative stress, mitochondrial impairment, insulin resistance, and metabolic disturbances affecting multiple organs. Consequently, the available cellular evidence provides a strong mechanistic rationale for further investigation of sulforaphane and broccoli-derived preparations in animal models and human intervention studies. The principal molecular pathways through which sulforaphane and broccoli-derived preparations may modulate obesity-related biological processes are summarized in Figure 2.

Nevertheless, several important limitations should be considered when interpreting the available in vitro evidence. Most studies have been performed in simplified cellular systems that cannot fully reproduce the complex multicellular, endocrine, nutritional, and inflammatory environment characteristic of human obesity. Moreover, considerable heterogeneity exists among cell types, experimental models, treatment protocols, exposure durations, and outcome measures, making direct comparisons among studies difficult. An additional and important translational limitation is that many experiments employ sulforaphane concentrations that may exceed physiologically achievable tissue exposures following dietary consumption of cruciferous vegetables or currently available broccoli-derived nutritional supplements.

Indeed, the concentrations of sulforaphane used in most mechanistic studies typically range from approximately 1 to 20 μM, with many biological effects reported at 5–10 μM and some investigations extending to 20 μM. Although these concentrations have been invaluable for elucidating molecular mechanisms, their physiological relevance remains uncertain. Following consumption of broccoli, broccoli sprouts, or glucoraphanin-containing preparations, circulating concentrations of sulforaphane and its metabolites are generally lower than those frequently employed in in vitro studies and are influenced by numerous factors, including the food matrix, myrosinase activity, formulation, administered dose, gastrointestinal metabolism, gut microbiota composition, and interindividual metabolic variability. Furthermore, although plasma pharmacokinetics have been reasonably well characterized, information regarding sulforaphane distribution, metabolism, and accumulation within metabolically relevant tissues, including adipose tissue, liver, and skeletal muscle, remains limited. Accordingly, the concentrations employed in many mechanistic cellular studies should not be assumed to reflect tissue concentrations achieved following dietary broccoli consumption or oral supplementation with broccoli-derived preparations. Consequently, it remains difficult to determine whether the concentrations required to elicit many of the reported cellular effects are achieved in vivo. Future studies integrating pharmacokinetic, tissue-distribution, and mechanistic investigations will therefore be essential for defining biologically effective and physiologically attainable exposure levels and for improving the translational interpretation of in vitro findings.

Overall, the current in vitro literature provides strong mechanistic support for the biological plausibility of sulforaphane and broccoli-derived preparations as promising nutritional strategies for improving obesity-associated metabolic dysfunction. However, these findings should be interpreted as mechanistic and hypothesis-generating rather than as direct evidence of anti-obesity efficacy. Likewise, the molecular effects observed under experimental conditions should not be directly extrapolated to the physiological effects expected following dietary broccoli consumption or nutritional supplementation without confirmation in appropriate animal models and well-designed randomized clinical trials. Confirmation of the observed cellular effects in well-designed animal studies and randomized clinical trials will be essential to determine whether modulation of these molecular pathways translates into clinically meaningful improvements in obesity, metabolic health, and obesity-related complications.

## 3. Evidence from In Vivo Animal Studies

Animal studies have provided substantial preclinical evidence supporting the potential role of sulforaphane and broccoli-derived preparations in modulating obesity and obesity-related metabolic dysfunction. While in vitro investigations provide mechanistic insight into cellular and molecular pathways, in vivo animal models enable evaluation of the integrated physiological interactions among adipose tissue, liver, skeletal muscle, gastrointestinal tract, endocrine organs, immune cells, and the central nervous system under conditions that more closely resemble the complexity of whole-organism metabolism [28,31,33,34,35,36,38,39,41,42,45,46,59,64,65,66,67,68,69,70,71,72,73,74,75,76,77,78,79,80,81,82,83]. Consequently, animal studies represent a critical intermediate step in the translational continuum between mechanistic cellular observations and human clinical investigations.

As summarized in Table 2, the available in vivo studies comprise both direct obesity-related animal models, which primarily evaluate body weight, adiposity, adipose tissue remodeling, adipocyte hypertrophy, energy expenditure, thermogenesis, and glucose homeostasis, and supportive mechanistic models, which investigate obesity-associated metabolic abnormalities, including insulin resistance, MASLD, chronic inflammation, oxidative stress, mitochondrial dysfunction, endothelial dysfunction, diabetic complications, and other obesity-related metabolic disturbances. Although these complementary models do not always directly assess obesity-related endpoints such as body weight or adiposity, they provide important mechanistic evidence regarding the biological pathways through which sulforaphane may influence obesity-associated metabolic dysfunction.

Most experimental investigations have employed high-fat diet (HFD)-induced obesity models in mice or rats, which reproduce many of the metabolic abnormalities observed in human obesity, including excessive body weight gain, adipocyte hypertrophy, insulin resistance, dyslipidemia, chronic low-grade inflammation, and MASLD. Additional studies have utilized genetically obese animals, diabetic models, transgenic mice, and disease-specific experimental models to investigate the molecular mechanisms underlying the metabolic actions of sulforaphane in obesity-associated complications. Collectively, these studies indicate that sulforaphane exerts pleiotropic biological activities through coordinated modulation of multiple interconnected signaling pathways implicated in obesity pathophysiology. However, it is important to distinguish evidence demonstrating direct effects on obesity-related phenotypes, such as reductions in body weight, adiposity, and adipose tissue dysfunction, from studies providing supportive mechanistic evidence through improvements in metabolic, inflammatory, oxidative, mitochondrial, or organ-specific abnormalities associated with obesity. This distinction is essential for accurately interpreting the current preclinical evidence and its translational relevance to human obesity.

### 3.1. Animal Models Used to Investigate the Effects of Sulforaphane

Most preclinical studies evaluating the metabolic effects of sulforaphane and broccoli-derived preparations have employed diet-induced obesity (DIO) models, which provide the principal source of direct obesity-related evidence [85,86,87,88,89,90,91,92,93]. These models typically involve long-term consumption of HFDs providing approximately 45–60% of total caloric intake from fat for periods ranging from 8 to 24 weeks [85,86,87,88,89,90,91,92,93]. Chronic HFD feeding induces progressive body weight gain, adipocyte hypertrophy, increased adiposity, insulin resistance, dyslipidemia, chronic low-grade inflammation, oxidative stress, and MASLD, thereby reproducing many of the metabolic abnormalities observed in human obesity [85,86,87,88,89,90,91,92,93]. Because obesity develops gradually in response to sustained energy excess, DIO models are widely regarded as among the most physiologically relevant preclinical approaches for evaluating interventions targeting obesity and its associated metabolic dysfunction [85,86,87,88,89,90,91,92,93].

Among rodent models, C57BL/6J mice are the most frequently used strain because of their high susceptibility to diet-induced obesity, glucose intolerance, insulin resistance, adipose tissue expansion, hepatic steatosis, and chronic inflammation following HFD feeding [85,86,87,88,89,90,91,92]. Their well-characterized metabolic phenotype and reproducible response to obesogenic diets have established them as one of the standard experimental models for obesity research [85,86,87,88,89,90,91,92]. Several studies have demonstrated that sulforaphane administration or sulforaphane-generating broccoli-derived preparations attenuate body weight gain, reduce adiposity, improve glucose homeostasis, and ameliorate obesity-associated metabolic abnormalities in HFD-fed C57BL/6J mice [31,32,33,34,39,42,51,59,68,70,75,77,78,79,80]. Notably, two landmark studies by Liu et al. and Choi et al. employed the closely related C57BL/6N strain rather than C57BL/6J and similarly demonstrated reduced adiposity, enhanced thermogenesis, and improved metabolic function following sulforaphane treatment [28,66]. Furthermore, Çubuk et al. extended these observations using a high-glycemic-index diet model in C57BL/6 mice, demonstrating that sulforaphane both prevented the development of obesity-associated metabolic abnormalities and improved established metabolic dysfunction by reducing body weight gain, improving glycemic control, and enhancing insulin sensitivity [81]. Comparable metabolic benefits have also been reported in HFD-fed Sprague–Dawley rats [64,67] and Wistar rats [35,36,71,72,82], indicating that the beneficial actions of sulforaphane are reproducible across multiple rodent strains.

In addition to DIO models, several investigations have utilized genetically obese mice, providing further direct evidence for the effects of sulforaphane on obesity-related phenotypes. In leptin-deficient ob/ob mice, sulforaphane or sulforaphane-rich broccoli leaf extracts reduced obesity, improved serum lipid and glucose metabolism, attenuated hepatic lipid accumulation, and activated AMPK-dependent pathways involved in energy homeostasis [38]. Mechanistic studies employing leptin-deficient (Lep^ob/ob^) and leptin receptor-deficient (Lepr^db/db^) mice further demonstrated that the weight-reducing effects of sulforaphane depend, at least in part, on intact leptin receptor signaling, suggesting that reversal of leptin resistance contributes to its metabolic actions [73]. Collectively, these studies strengthen the evidence that sulforaphane directly modulates obesity-related phenotypes, including body weight regulation, adiposity, and adipose tissue remodeling.

In contrast, several additional animal models have been employed primarily to investigate obesity-associated metabolic complications and therefore provide supportive mechanistic evidence rather than direct evidence of anti-obesity efficacy. Streptozotocin (STZ)-induced diabetic models and combined HFD/low-dose STZ models have been widely used to evaluate the effects of sulforaphane on insulin resistance, glucose homeostasis, oxidative stress, dyslipidemia, and MASLD, which represent common metabolic complications of obesity [46,65]. Likewise, high-fructose-fed rat models have been utilized to investigate insulin resistance, hepatosteatosis, vascular dysfunction, oxidative stress, and chronic inflammation following sulforaphane administration [67,82,83]. Although these models do not primarily evaluate body weight or adiposity, they provide important mechanistic insight into the molecular pathways through which sulforaphane may influence obesity-associated metabolic dysfunction.

Finally, several specialized experimental models have been employed to elucidate the molecular mechanisms underlying the metabolic actions of sulforaphane. Studies using Nrf2-deficient mice have demonstrated that disruption of Nrf2 signaling markedly attenuates many of the beneficial effects of sulforaphane, including improvements in adiposity, insulin sensitivity, oxidative stress, and inflammation, highlighting the central role of the Nrf2 pathway in mediating its metabolic actions [73]. Similarly, mechanistic studies using SV129 humanized CYP2E1 knock-in mice [41] and ICR mice [45] have clarified the involvement of oxidative stress and antioxidant signaling pathways. Additional investigations employing cold-exposed mice have examined thermogenic responses, whereas microbiota-focused models have explored interactions between sulforaphane and the gut microbiome [28,69]. Although these models are not designed primarily to evaluate obesity outcomes, they provide valuable mechanistic evidence supporting the biological pathways through which sulforaphane may influence obesity-associated metabolic dysfunction.

Despite their considerable value, animal models possess inherent limitations. Laboratory animals are maintained under highly controlled environmental conditions and exhibit relatively homogeneous genetic backgrounds that differ substantially from the genetic, environmental, dietary, and lifestyle heterogeneity characteristic of human populations. Furthermore, differences in species, experimental diets, treatment duration, dosing regimens, and sulforaphane bioavailability may influence the observed metabolic responses. Consequently, findings derived from animal studies should be interpreted as preclinical evidence supporting biological plausibility rather than definitive evidence of clinical efficacy, emphasizing the need for well-designed human intervention studies to establish the translational relevance of these observations.

### 3.2. Effects on Body Weight and Adiposity

Reduction in body weight gain and adiposity represents one of the most consistent findings reported in animal studies investigating the effects of sulforaphane and broccoli-derived preparations. Across multiple diet-induced and genetic models of obesity, sulforaphane consistently attenuates body weight gain, reduces adipose tissue mass, limits adipocyte hypertrophy, and improves body composition, providing some of the strongest direct preclinical evidence supporting its potential role in obesity management [28,31,35,36,38,39,45,64,66,68,70,71,72,73,74,77,78,79,80,81,82,84].

Early evidence was provided by Choi et al., who demonstrated that sulforaphane significantly attenuated HFD-induced obesity in C57BL/6N mice by reducing body weight gain, adipose tissue mass, adipocyte hypertrophy, and hepatic lipid accumulation through activation of AMPK signaling and suppression of adipogenesis [66]. These findings were subsequently confirmed by Nagata et al., who reported that dietary glucoraphanin supplementation reduced body weight gain, visceral adiposity, hepatic steatosis, and insulin resistance in HFD-fed C57BL/6J mice while simultaneously promoting adipose tissue browning, increasing UCP1 expression, reducing metabolic endotoxemia, and activating Nrf2-dependent antioxidant pathways [84].

Comparable findings have been reported in several independent studies. Liu et al. demonstrated that sulforaphane reduced fat mass and adipocyte size while promoting white adipose tissue browning, mitochondrial biogenesis, energy expenditure, and insulin sensitivity in obese mice [28]. Likewise, Yang et al. showed that sulforaphane suppressed HFD-induced obesity through enhancement of skeletal muscle mitochondrial biogenesis and increased energy expenditure [31]. Additional investigations employing dietary or pharmacological administration of sulforaphane have consistently demonstrated reductions in adiposity together with improvements in glucose and lipid metabolism and attenuation of obesity-associated metabolic dysfunction [36,71,79,80].

Evidence supporting therapeutic efficacy has also emerged from studies evaluating established obesity. Although most investigations assessed prevention of HFD-induced weight gain, Çakır et al. demonstrated that sulforaphane induced significant weight loss in already obese mice through restoration of leptin sensitivity and reversal of leptin resistance [73]. Similarly, Luo et al. reported that oral sulforaphane administration reduced adiposity and improved insulin sensitivity in diet-induced obese mice without altering food intake, indicating therapeutic benefits beyond obesity prevention [79]. Furthermore, Çubuk et al. demonstrated that sulforaphane attenuated obesity-associated metabolic abnormalities induced by a high-glycemic-index diet, improving body weight regulation, glycemic control, insulin sensitivity, and lipid metabolism [81].

Histological analyses consistently support these physiological findings. Across multiple obesity models, sulforaphane or sulforaphane-generating preparations reduced adipocyte hypertrophy, limited excessive adipose tissue expansion, and improved adipose tissue architecture [28,38,66,79]. Several studies further demonstrated reductions in macrophage infiltration, adipose tissue inflammation, fibrosis, and obesity-associated tissue remodeling, including promotion of anti-inflammatory M2 macrophage polarization and attenuation of adipose tissue fibrosis [77,84]. These histological improvements are consistent with healthier adipose tissue remodeling and reduced chronic adipose tissue inflammation during obesity. Although suppression of adipogenic regulators such as PPARγ and C/EBPα suggests inhibition of adipocyte hyperplasia, most animal studies quantified adipocyte size rather than adipocyte number. Consequently, current evidence more strongly supports reductions in adipocyte hypertrophy than direct inhibition of adipocyte hyperplasia in vivo [28,38,66,79].

Several complementary mechanisms appear to contribute to these reductions in body weight and adiposity. Animal studies consistently identify activation of AMPK as a central regulatory event linking sulforaphane administration to inhibition of adipogenesis, suppression of lipogenesis, stimulation of fatty acid oxidation, enhancement of mitochondrial function, and increased energy expenditure [35,45,46,66,72,80]. Additional studies implicate promotion of adipose tissue browning, improved skeletal muscle oxidative metabolism, modulation of gut microbiota composition, and enhancement of metabolic flexibility as complementary mechanisms contributing to reduced adiposity [28,31,68,69,70,74,79,80,84].

Importantly, several investigations reported significant reductions in body weight gain and adiposity despite minimal or no changes in food intake [28,45,70,79]. These observations suggest that the anti-obesity effects of sulforaphane are mediated predominantly through increased energy expenditure and improved metabolic efficiency rather than appetite suppression. Enhanced thermogenesis, adipose tissue browning, mitochondrial biogenesis, and fatty acid oxidation therefore appear to play central roles in the regulation of energy balance following sulforaphane administration [28,45,70,79].

Despite the consistency of these findings, several limitations should be acknowledged. Considerable variability exists among studies with respect to animal strain, sex, dietary composition, obesity severity, treatment duration, sulforaphane formulation, and administered dose, all of which may influence the magnitude of the observed effects. Furthermore, many studies employ doses substantially higher than those achievable through normal dietary consumption of cruciferous vegetables, which may limit direct translation of these findings to humans. Consequently, although animal studies provide compelling direct preclinical evidence that sulforaphane attenuates body weight gain and adiposity, confirmation in well-designed human clinical trials remains essential before these findings can be translated into clinical recommendations.

### 3.3. Effects on Energy Expenditure and Thermogenesis

Increasing energy expenditure through adaptive thermogenesis represents an attractive strategy for obesity prevention and treatment. Animal studies provide consistent direct obesity-related evidence that sulforaphane and broccoli-derived preparations enhance thermogenic activity and promote adipose tissue browning, thereby contributing to reductions in body weight gain and adiposity [28,31,74,79,80,84].

As discussed in Section 3.2, several studies have reported significant reductions in body weight gain and fat mass despite minimal or no changes in food intake, suggesting that increased energy expenditure, rather than appetite suppression, constitutes a major mechanism underlying the anti-obesity effects of sulforaphane [28,45,70,79,84]. One of the earliest demonstrations was provided by Nagata et al., who showed that dietary glucoraphanin increased oxygen consumption, elevated core body temperature, enhanced thermogenic gene expression, and reduced metabolic endotoxemia in HFD-fed mice, collectively indicating increased energy expenditure [84]. Likewise, Liu et al. demonstrated that sulforaphane reduced adiposity and adipocyte hypertrophy while promoting white adipose tissue browning and increasing the expression of key thermogenic regulators, including UCP1, PGC-1α, PR domain containing 16 (PRDM16), cell death-inducing DFFA-like effector A (CIDEA), and transmembrane protein 26 (TMEM26), together with enhanced mitochondrial biogenesis [28].

These findings indicate that sulforaphane promotes the conversion of energy-storing white adipocytes toward a metabolically active beige phenotype capable of dissipating excess energy as heat. The coordinated upregulation of UCP1, PGC-1α, and PRDM16 is consistent with activation of the canonical thermogenic program, whereas increased expression of CIDEA and TMEM26 further supports enhanced beige adipocyte differentiation and thermogenic competence [27,28].

Animal studies further demonstrate that enhanced thermogenesis is accompanied by improvements in mitochondrial function and oxidative metabolism. Increased expression of regulators of mitochondrial biogenesis and fatty acid oxidation, including NRF1, TFAM, carnitine palmitoyltransferase-1 (CPT-1), and other mitochondrial respiratory proteins, has been reported following sulforaphane administration [28,31]. These adaptations are associated with increased mitochondrial content, enhanced fatty acid utilization, greater oxidative metabolism, improved glucose utilization, and increased metabolic flexibility [27,28,31,79]. Consistent with these findings, Yang et al. demonstrated that sulforaphane enhanced skeletal muscle mitochondrial biogenesis through activation of the HDAC8–PGC-1α pathway, whereas Luo et al. reported improved skeletal muscle oxidative metabolism and mitochondrial function in obese mice, indicating that both adipose tissue and skeletal muscle contribute to increased whole-body energy expenditure [31,79].

Several studies suggest that activation of AMPK represents a central upstream event linking sulforaphane administration to enhanced thermogenesis. Activation of AMPK promotes PGC-1α-mediated mitochondrial biogenesis, stimulates fatty acid oxidation through activation of CPT-1, suppresses lipogenesis by inhibiting ACC, SREBP-1c, FAS, and stearoyl-CoA desaturase-1 (SCD1), and shifts cellular metabolism toward increased energy expenditure [28,35,66]. More recently, Jeon et al. identified activation of the 5-hydroxytryptamine receptor 2A (5-HT2A)/AMPK signaling pathway as an additional mechanism mediating the thermogenic effects of sulforaphane-rich broccoli seed extracts, whereas Çakır et al. demonstrated that restoration of leptin sensitivity may further contribute to enhanced energy balance and weight reduction in obese animals [73,80].

In addition to directly stimulating thermogenic pathways, sulforaphane appears to improve the adipose tissue microenvironment, thereby facilitating thermogenic remodeling. Chronic obesity is characterized by adipose tissue fibrosis, extracellular matrix remodeling, mitochondrial dysfunction, and infiltration of pro-inflammatory macrophages, all of which impair beige adipocyte formation. Animal studies have demonstrated that sulforaphane attenuates adipose tissue fibrosis, promotes macrophage polarization toward the anti-inflammatory M2 phenotype through Nrf2- and Krüppel-like factor 4 (KLF4)-dependent mechanisms, and suppresses NF-κB- and NLRP3 inflammasome-mediated inflammatory signaling [45,77,78]. These changes reduce production of pro-inflammatory cytokines, including TNF-α, IL-6, and IL-1β, creating a tissue environment more permissive for mitochondrial biogenesis, adipose tissue browning, and sustained thermogenic activity [45,77,78].

Although the available evidence consistently supports thermogenic activation as an important contributor to the anti-obesity effects of sulforaphane, several limitations should be considered. Most studies infer increased thermogenesis from changes in thermogenic gene or protein expression, whereas direct assessments of whole-body energy expenditure, including indirect calorimetry, oxygen consumption, respiratory exchange ratio, heat production, or mitochondrial respiration, remain comparatively limited. Consequently, increased expression of thermogenic markers does not necessarily translate into proportional increases in whole-body energy expenditure. Furthermore, considerable heterogeneity exists among studies regarding animal strain, dietary model, treatment duration, sulforaphane formulation, and administered dose, and many experimental doses exceed those likely to be achieved through habitual dietary consumption of cruciferous vegetables. Therefore, although animal studies provide compelling evidence that sulforaphane promotes adipose tissue browning, mitochondrial biogenesis, and thermogenic programming, the physiological significance and translational relevance of these adaptations require confirmation in well-designed human intervention studies.

### 3.4. Effects on Insulin Resistance and Glucose Homeostasis

Improvement of insulin resistance and glucose homeostasis represents one of the most consistently reported metabolic benefits of sulforaphane and broccoli-derived preparations in animal studies. Although many investigations employed obesity models, a substantial proportion utilized diabetic or mixed metabolic disease models. Consequently, the available evidence should be interpreted as supportive mechanistic evidence demonstrating the ability of sulforaphane to ameliorate obesity-associated insulin resistance and glucose dysregulation rather than as direct evidence of anti-obesity efficacy [45,51,65,67,70,79].

Across HFD-induced obesity, high-fructose, high-fat/high-fructose, high-glycemic-index, and STZ-induced diabetic models, sulforaphane consistently reduced fasting blood glucose and insulin concentrations, improved glucose tolerance, attenuated insulin resistance, and enhanced overall metabolic control [45,51,65,67,70,79]. These beneficial effects have been observed in both preventive and therapeutic experimental settings, indicating that sulforaphane improves glucose metabolism across a broad spectrum of obesity-associated metabolic disorders.

Several complementary mechanisms appear to underlie these metabolic improvements. Activation of AMPK has consistently been identified as a central regulatory event linking sulforaphane administration to enhanced glucose utilization, increased fatty acid oxidation, reduced ectopic lipid accumulation, and improved insulin responsiveness [45,66,72]. Likewise, sulforaphane enhances insulin receptor signaling through activation of the IRS-1/PI3K)/Ak pathway, thereby promoting GLUT4 translocation, increasing peripheral glucose uptake, suppressing hepatic glucose production, and improving intracellular glucose utilization [51,68,75].

Additional animal studies indicate that suppression of stress- and inflammation-associated signaling pathways further contributes to improved insulin sensitivity. Tian et al. demonstrated that sulforaphane inhibited c-Jun N-terminal kinase (JNK) activation while simultaneously stimulating fibroblast growth factor-21 (FGF21) signaling, thereby preserving insulin receptor signaling and enhancing systemic insulin sensitivity [70]. Similarly, activation of the Kelch-like ECH-associated protein 1 (Keap1)- Nrf2 pathway has been associated with reductions in oxidative stress, lipid peroxidation, and ferroptotic signaling, resulting in improved insulin responsiveness and metabolic homeostasis [45]. These antioxidant effects likely protect insulin signaling pathways from oxidative damage while preserving mitochondrial function.

Chronic low-grade inflammation also represents an important therapeutic target. Animal studies have demonstrated that sulforaphane suppresses multiple inflammatory pathways implicated in obesity-associated insulin resistance, including NF-κB, toll-like receptor 4 (TLR4), JNK, Janus kinase 2/signal transducer and activator of transcription 3 (JAK2/STAT3), and NLRP3 inflammasome signaling [71,75,77,78]. These changes are accompanied by reduced production of TNF-α, IL-6, IL-1β, and monocyte chemoattractant protein-1 (MCP-1), together with promotion of anti-inflammatory M2 macrophage polarization, thereby creating a tissue microenvironment that favors restoration of insulin sensitivity [71,75,77,78].

Improved hepatic metabolism appears to constitute another important component of the insulin-sensitizing effects of sulforaphane. In animal models of obesity-associated MASLD, sulforaphane reduced hepatic triglyceride accumulation, suppressed lipogenic signaling, attenuated endoplasmic reticulum stress, improved mitochondrial function, and restored hepatic insulin responsiveness [28,70,72,75]. Complementary studies further suggest that modulation of the gut–liver axis contributes to these metabolic benefits. Glucoraphanin supplementation reduced circulating lipopolysaccharide concentrations and metabolic endotoxemia, whereas sulforaphane restored intestinal barrier integrity and suppressed lipopolysaccharide/TLR4 signaling, thereby reducing systemic inflammation and improving glucose homeostasis [68,75,89].

Skeletal muscle also contributes substantially to these metabolic adaptations. Yang et al. demonstrated that sulforaphane enhanced skeletal muscle mitochondrial biogenesis through activation of the HDAC8-PGC-1α pathway, whereas Luo et al. reported improvements in skeletal muscle oxidative metabolism, mitochondrial function, metabolic flexibility, and glucose utilization in obese mice [31,79]. Because skeletal muscle is responsible for most insulin-stimulated glucose disposal, these adaptations likely contribute substantially to improved whole-body glucose homeostasis. Evidence from STZ-induced diabetic models further supports the glucose-regulatory potential of sulforaphane, demonstrating improvements in insulin sensitivity, hepatic glycogen storage, lipid metabolism, antioxidant defenses, inflammatory status, and pancreatic β-cell preservation [46,65,70].

Collectively, the available animal evidence demonstrates that sulforaphane consistently improves insulin resistance and glucose homeostasis through coordinated regulation of multiple complementary pathways, including enhancement of insulin signaling, attenuation of oxidative and inflammatory stress, improvement of hepatic and skeletal muscle metabolism, preservation of mitochondrial function, and modulation of the gut–liver axis. However, because many studies employed diabetic or mixed metabolic disease models rather than obesity models alone, these findings should be interpreted primarily as supportive mechanistic evidence for the beneficial effects of sulforaphane on obesity-associated metabolic dysfunction rather than as direct evidence of anti-obesity efficacy. Furthermore, most investigations utilized relatively short intervention periods, high doses of sulforaphane, and limited metabolic phenotyping, while hyperinsulinemic-euglycemic clamp studies remain scarce. Therefore, additional long-term preclinical and clinical studies are needed to establish dose–response relationships, tissue-specific mechanisms, and the translational relevance of these findings for human obesity and metabolic disease.

### 3.5. Effects on Hepatic Steatosis

MASLD is one of the most prevalent metabolic complications of obesity and is characterized by excessive hepatic lipid accumulation, insulin resistance, oxidative stress, mitochondrial dysfunction, chronic low-grade inflammation, and progressive liver injury [91]. Consequently, animal studies evaluating the effects of sulforaphane and broccoli-derived preparations in MASLD models provide supportive mechanistic evidence regarding obesity-associated metabolic dysfunction rather than direct evidence of anti-obesity efficacy. Nevertheless, a substantial body of preclinical evidence demonstrates that sulforaphane consistently attenuates hepatic steatosis, reduces hepatic triglyceride accumulation, improves liver histology, and ameliorates obesity-associated metabolic abnormalities [33,67,75,76].

Across multiple HFD-, high-fat/high-fructose-, and fructose-induced models of MASLD, sulforaphane has consistently reduced hepatic lipid accumulation while improving liver morphology and metabolic function [33,35,67,72,75,76]. These improvements are largely attributed to coordinated regulation of hepatic lipid metabolism, including suppression of de novo lipogenesis together with stimulation of mitochondrial and peroxisomal fatty acid oxidation. Animal studies have demonstrated downregulation of major lipogenic regulators, including SREBP-1c, FAS, ACC, and SCD1, accompanied by increased expression of genes involved in β-oxidation, such as CPT-1, acyl-CoA oxidase-1 (ACOX1), and PPARα [33,35,66,70].

Several studies identify activation of AMPK as a central mechanism underlying these hepatoprotective effects. Sulforaphane-mediated AMPK activation suppresses hepatic lipogenesis, enhances fatty acid oxidation, improves insulin sensitivity, and reduces hepatic triglyceride accumulation in experimental models of obesity and MASLD [66,72]. Additional studies demonstrate improvements in mitochondrial function and oxidative metabolism, resulting in more efficient lipid utilization and reduced hepatic lipid overload [79]. These adaptations likely contribute to attenuation of lipotoxicity, oxidative stress, and progression of hepatic injury.

Oxidative stress and endoplasmic reticulum (ER) stress also represent important therapeutic targets. Activation of the Keap1-Nrf2 pathway enhances endogenous antioxidant defenses, preserves mitochondrial integrity, and reduces lipid peroxidation and hepatocellular injury [41]. Consistent with these observations, several animal studies reported reductions in oxidative stress biomarkers together with improvements in liver histology following sulforaphane administration [66,82]. Similarly, Tian et al. demonstrated that sulforaphane improved abnormal hepatic lipid metabolism through modulation of X-box binding protein 1 (XBP1)-dependent ER stress pathways, whereas Mansour et al. showed that attenuation of ER stress through AMPK activation contributed to restoration of hepatic metabolic homeostasis [35,72].

Accumulating evidence further indicates that suppression of hepatic inflammation contributes substantially to the anti-steatotic effects of sulforaphane. Xu et al. demonstrated that sulforaphane ameliorated high-fat/high-fructose-induced MASLD through modulation of the lipopolysaccharide (LPS)/TLR4 signaling pathway within the gut–liver axis, resulting in reduced hepatic inflammation and improved metabolic function [75]. Likewise, Huang et al. reported that sulforaphane attenuated metabolic dysfunction-associated steatohepatitis (MASH) by promoting Krüppel-like factor 4 (KLF4)-mediated polarization of macrophages toward an anti-inflammatory M2 phenotype, thereby reducing hepatic inflammatory responses and improving liver pathology [78]. These findings indicate that sulforaphane not only limits hepatic lipid accumulation but also suppresses inflammatory mechanisms responsible for disease progression.

Modulation of the gut microbiota represents another emerging mechanism contributing to hepatic protection. Animal studies have demonstrated that glucoraphanin and sulforaphane improve gut microbial composition, reduce metabolic endotoxemia, strengthen intestinal barrier integrity, and modify host metabolic profiles [68,76]. Reduced translocation of bacterial endotoxins into the portal circulation likely attenuates hepatic TLR4 activation and downstream inflammatory signaling, thereby contributing to improvements in hepatic steatosis, insulin sensitivity, and overall metabolic homeostasis [68,75,76].

Beyond reducing hepatic lipid accumulation, sulforaphane also appears to improve several metabolic abnormalities that contribute to progressive liver injury. Animal studies have demonstrated improvements in dyslipidemia, insulin resistance, oxidative stress, endothelial dysfunction, and systemic inflammation in fructose-fed and HFD-induced models [67,82,83]. More recent investigations in MASLD and MASH models further reported reductions in hepatocellular injury, inflammatory infiltration, and overall metabolic dysfunction, suggesting that the beneficial effects of sulforaphane extend beyond simple steatosis and may delay progression toward more advanced stages of liver disease [75,76].

Despite these encouraging findings, several limitations should be considered. Most studies evaluated early-stage hepatic steatosis and metabolic dysfunction, whereas comparatively few investigated advanced outcomes such as significant fibrosis, cirrhosis, hepatocellular carcinoma, or long-term liver-related morbidity. Furthermore, considerable variability exists among experimental models, treatment protocols, and sulforaphane formulations, and many studies employed pharmacological doses that exceed those achievable through normal dietary consumption of cruciferous vegetables. Consequently, although current animal evidence strongly supports the hepatoprotective effects of sulforaphane and provides important supportive mechanistic evidence for its role in ameliorating obesity-associated metabolic dysfunction, additional long-term preclinical studies and well-designed clinical trials are required to determine its efficacy against advanced MASLD and establish its translational relevance in humans.

### 3.6. Effects on Inflammation and Oxidative Stress

Chronic low-grade inflammation and oxidative stress are fundamental mechanisms linking obesity to insulin resistance, adipose tissue dysfunction, MASLD, endothelial dysfunction, and other obesity-associated metabolic complications [91]. Consequently, animal studies investigating these outcomes provide supportive mechanistic evidence for the metabolic benefits of sulforaphane rather than direct evidence of anti-obesity efficacy. Nevertheless, a substantial body of preclinical evidence demonstrates that sulforaphane and sulforaphane-generating preparations consistently attenuate inflammatory responses and oxidative stress across multiple models of obesity and metabolic disease [45,67,82].

Animal studies consistently report reductions in systemic and tissue inflammatory markers following sulforaphane administration. Decreased concentrations of TNF-α, IL-6, IL-1β, MCP-1, and other pro-inflammatory mediators have been observed in adipose tissue, liver, and circulation, accompanied by improvements in insulin sensitivity, hepatic steatosis, and overall metabolic function [66,82]. These findings indicate that suppression of chronic metabolic inflammation represents an important contributor to the metabolic benefits of sulforaphane.

Several complementary molecular pathways appear to mediate these anti-inflammatory effects. Animal studies demonstrate that sulforaphane suppresses activation of NF-κB, TLR4, JNK, and NLRP3 inflammasome signaling, thereby reducing transcription and release of multiple pro-inflammatory cytokines implicated in obesity-associated metabolic dysfunction [45,46,75,78]. Inhibition of these inflammatory pathways likely contributes to improved insulin signaling, attenuation of adipose tissue inflammation, and protection against progression of hepatic steatosis toward metabolic dysfunction-associated steatohepatitis (MASH).

In addition to suppressing inflammatory signaling, sulforaphane favorably modifies immune cell composition within metabolically active tissues. Nagata et al. demonstrated that glucoraphanin supplementation reduced metabolic endotoxemia and obesity-associated inflammation while improving insulin sensitivity in HFD-fed mice [84]. More recently, Zhang et al. reported reduced adipose tissue fibrosis and enhanced macrophage polarization toward the anti-inflammatory M2 phenotype [77], whereas Huang et al. demonstrated that sulforaphane attenuated MASH through KLF4-mediated M2 macrophage polarization, resulting in reduced hepatic inflammation and tissue injury [78]. These observations suggest that sulforaphane promotes resolution of chronic inflammation in addition to suppressing pro-inflammatory signaling.

Emerging evidence also supports an important contribution of the gut–liver–adipose axis to the anti-inflammatory effects of sulforaphane. Obesity-associated gut dysbiosis and increased intestinal permeability promote metabolic endotoxemia through translocation of bacterial LPS, leading to activation of TLR4-dependent inflammatory pathways. Xu et al. demonstrated that sulforaphane ameliorated high-fat/high-fructose-induced MASLD by modulating LPS/TLR4 signaling within the gut–liver axis [75]. Complementary studies involving glucoraphanin-rich preparations further demonstrated favorable alterations in gut microbiota composition and host metabolic profiles [68,76], suggesting that improved intestinal barrier function and reduced endotoxin exposure contribute to attenuation of systemic inflammation.

Parallel to its anti-inflammatory activity, sulforaphane exerts potent antioxidant effects through activation of the Keap1-Nrf2 pathway. Activation of Nrf2 enhances endogenous antioxidant defenses by increasing expression of multiple cytoprotective enzymes, including heme oxygenase-1 (HO-1), NAD(P)H quinone oxidoreductase-1 (NQO1), GPx, glutamate-cysteine ligase, catalase, SOD, and other components of the glutathione antioxidant system [41,45]. Consistent with this mechanism, animal studies consistently demonstrate reductions in reactive oxygen species generation, lipid peroxidation, and oxidative damage together with improvements in insulin sensitivity, dyslipidemia, hepatic steatosis, endothelial dysfunction, and overall metabolic status [45,67,82,83]. Furthermore, Zhang et al. showed that activation of the AMPK/Nrf2/GPx4 signaling axis contributed to protection against lipid peroxidation and ferroptosis while improving metabolic homeostasis [45].

Additional studies suggest that attenuation of endoplasmic reticulum (ER) stress and preservation of mitochondrial function further contribute to the antioxidant and anti-inflammatory actions of sulforaphane. Mansour et al. demonstrated that sulforaphane alleviated ER stress through AMPK activation in experimental MASLD [72], whereas Tian et al. reported modulation of ER stress-associated pathways involving XBP1, ACC, and SCD1 [35]. Animal studies also demonstrate enhanced mitochondrial biogenesis, oxidative capacity, and metabolic flexibility following sulforaphane administration through mechanisms involving AMPK activation and increased expression of peroxisome PGC-1α and other mitochondrial regulators [28,31,79]. These adaptations likely reduce oxidative stress at its source while restoring cellular energy homeostasis.

Collectively, the available animal evidence demonstrates that sulforaphane consistently attenuates obesity-associated inflammation and oxidative stress through coordinated modulation of inflammatory signaling, antioxidant defenses, immune cell polarization, mitochondrial function, ER stress, and the gut–liver–adipose axis. These complementary mechanisms contribute to improvements in insulin sensitivity, preservation of adipose tissue and hepatic function, and attenuation of obesity-associated metabolic complications. However, because inflammation and oxidative stress represent downstream manifestations of obesity rather than obesity itself, these findings should be interpreted primarily as supportive mechanistic evidence explaining the beneficial metabolic effects of sulforaphane. Furthermore, many studies employed relatively high experimental doses, short intervention periods, and surrogate biomarkers of oxidative stress and inflammation. Therefore, additional long-term animal studies and well-designed clinical trials are required to determine the extent to which these molecular adaptations translate into clinically meaningful benefits in individuals with obesity.

### 3.7. Effects on Leptin Sensitivity, Appetite Regulation, and Gut–Brain Signaling

Compared with the evidence supporting the effects of sulforaphane on adiposity, thermogenesis, insulin resistance, and hepatic steatosis, considerably fewer animal studies have investigated its influence on central regulation of energy balance. Consequently, the available findings should be regarded as emerging supportive mechanistic evidence suggesting that sulforaphane may modulate leptin sensitivity, neuro-metabolic signaling, and gut–brain communication, thereby contributing indirectly to improvements in obesity-associated metabolic dysfunction [73,80].

The strongest evidence currently available derives from the study by Çakır et al., who demonstrated that sulforaphane induced significant weight loss in obese mice primarily through restoration of leptin sensitivity rather than direct appetite suppression [73]. Sulforaphane improved hypothalamic responsiveness to endogenous leptin and restored leptin receptor signaling, indicating that reversal of obesity-associated leptin resistance may contribute to improvements in energy balance and body weight regulation [73]. Because leptin resistance is a hallmark of obesity, these findings suggest that sulforaphane may influence central mechanisms regulating both energy intake and energy expenditure.

Additional studies indicate that attenuation of hypothalamic inflammation may underlie these beneficial effects. Obesity-associated activation of inflammatory pathways, including JAK2/STAT3/SOCS3 and NF-κB, impairs leptin receptor signaling and contributes to central leptin resistance. In this regard, Ashmawy et al. demonstrated modulation of the JAK2/STAT3/SOCS3 pathway in HFD-fed mice following sulforaphane administration [71], whereas Tian et al. reported suppression of JNK signaling together with enhancement of insulin and FGF2 signaling [70]. Although these studies primarily evaluated systemic metabolic responses rather than hypothalamic function directly, they support the hypothesis that attenuation of inflammatory signaling may improve central regulation of energy homeostasis.

Emerging evidence also suggests that neuro-metabolic signaling pathways may participate in the regulation of energy balance. Jeon et al. demonstrated that a sulforaphane-rich broccoli seed extract attenuated diet-induced obesity through activation of the 5-HT2A/AMPK signaling pathway, resulting in increased energy expenditure, improved metabolic function, and reduced adiposity [80]. Although the relative contributions of central and peripheral serotonergic signaling remain uncertain, these findings suggest that serotonergic regulation may contribute to the metabolic actions of sulforaphane. Likewise, activation of the Keap1-Nrf2 pathway and the accompanying reduction in oxidative stress may indirectly preserve hypothalamic neuronal function by limiting neuroinflammatory signaling and oxidative injury [45,82].

More consistent evidence is available regarding modulation of the gut microbiota. Animal studies have repeatedly demonstrated that glucoraphanin and sulforaphane alter gut microbial composition, increase microbial diversity, improve intestinal barrier integrity, and reduce metabolic endotoxemia [68,69,76,84]. Nagata et al. reported that glucoraphanin supplementation reduced circulating LPS concentrations and metabolic endotoxemia while improving obesity-associated insulin resistance and adipose tissue inflammation [84]. Similarly, Xu et al. demonstrated favorable alterations in gut microbial composition together with improvements in lipid metabolism [68]. More recent studies further showed that glucoraphanin improved MASLD through modulation of gut microbiota composition and host metabolic pathways [76,90], whereas Jun et al. demonstrated coordinated changes in both the intestinal microbiome and metabolome following sulforaphane administration [69]. These findings indicate that modulation of the gut microbiota represents one of the more reproducible metabolic actions of sulforaphane in animal models.

Alterations in the gut microbiota may influence host metabolism through multiple complementary mechanisms, including increased production of short-chain fatty acids, improved intestinal barrier integrity, altered bile acid metabolism, reduced endotoxin translocation, and attenuation of inflammatory signaling. These adaptations provide a plausible mechanistic link between gut microbial remodeling and improvements in systemic metabolic homeostasis. Consistent with this concept, Xu et al. demonstrated that sulforaphane ameliorated metabolic dysfunction through modulation of the LPS/TLR4 pathway within the gut–liver axis [75]. Although these observations support the existence of gut–brain communication, direct evidence that microbiota-mediated changes are responsible for improvements in hypothalamic function or appetite regulation remains limited.

Overall, current animal evidence suggests that sulforaphane influences energy balance through both peripheral and central mechanisms, including restoration of leptin sensitivity, attenuation of inflammatory signaling, modulation of neuro-metabolic pathways, and remodeling of the gut microbiota. However, the available evidence indicates that these mechanisms probably complement, rather than replace, the better-established actions of sulforaphane on thermogenesis, mitochondrial function, adipose tissue browning, and energy expenditure. Moreover, direct measurements of hypothalamic neuronal activity, appetite-regulating neuropeptides, food reward pathways, and gut–brain signaling remain scarce, and many reported central effects are inferred from systemic metabolic improvements rather than measured directly. Consequently, although the available studies provide promising supportive mechanistic evidence, additional mechanistic investigations and well-designed translational studies are required to establish the contribution of central nervous system and gut–brain signaling pathways to the anti-obesity effects of sulforaphane.

### 3.8. Critical Appraisal of the Preclinical Evidence and Translational Perspectives

Collectively, the available in vivo evidence indicates that sulforaphane and sulforaphane-generating preparations exert broad metabolic benefits across a wide range of experimental models of obesity and obesity-associated metabolic dysfunction. The strongest direct evidence derives from diet-induced and genetic obesity models, in which sulforaphane consistently attenuates body weight gain, reduces adiposity, limits adipocyte hypertrophy, promotes adipose tissue browning, enhances thermogenesis, and improves energy expenditure [28,31,66,73,79,80,84]. These findings support a direct role for sulforaphane in regulating energy balance and adipose tissue biology during obesity.

Complementing these observations, a substantial body of supportive mechanistic evidence demonstrates that sulforaphane ameliorates many of the metabolic abnormalities accompanying obesity, including insulin resistance, impaired glucose homeostasis, MASLD, chronic inflammation, oxidative stress, mitochondrial dysfunction, endoplasmic reticulum stress, gut microbiota dysbiosis, and alterations in neuro-metabolic signaling [35,41,45,46,51,65,67,68,70,71,72,75,76,77,78,79,82,84]. Although these findings do not directly demonstrate reductions in obesity, they provide important mechanistic insight into the biological pathways through which sulforaphane may improve obesity-associated metabolic dysfunction.

Overall, the available animal evidence suggests that the beneficial effects of sulforaphane result from coordinated modulation of multiple complementary pathways rather than a single molecular target. Activation of AMPK and Keap1-Nrf2 signaling emerges as a central mechanistic hub linking sulforaphane administration to enhanced fatty acid oxidation, adipose tissue browning, thermogenesis, mitochondrial biogenesis, and antioxidant defense, while simultaneously suppressing lipogenesis, inflammation, oxidative stress, and metabolic dysfunction. Emerging evidence further suggests additional contributions from restoration of leptin sensitivity, modulation of the gut microbiota, and regulation of the gut–liver and gut–brain axes, although these mechanisms remain less extensively characterized than the metabolic effects on adipose tissue and energy expenditure.

Despite the remarkable consistency of the preclinical findings, several important limitations should be acknowledged. Although purified sulforaphane is the predominant intervention used in experimental animal studies, substantial methodological heterogeneity exists with respect to the administered dose, route of administration (e.g., dietary incorporation, oral gavage, intraperitoneal injection, and, less frequently, subcutaneous administration), dosing frequency, treatment duration, timing of intervention (preventive versus therapeutic protocols), and experimental obesity models. Differences in animal species, genetic background, dietary composition, obesity severity, and study duration further complicate direct comparisons among investigations and preclude identification of an optimal dosing regimen for translation to humans. Consequently, while the overall direction of the metabolic effects is highly consistent, the relative efficacy of different dosing strategies and treatment protocols remains difficult to establish.

Furthermore, another important source of heterogeneity among the included animal studies was the considerable variation in experimental protocols, including differences in animal age, diet composition, housing temperature, comparator groups, study duration, and methodological practices such as randomization, blinding, and monitoring or control of food intake (e.g., pair-feeding). These variables were inconsistently reported across studies and may substantially influence metabolic outcomes, thereby limiting direct comparisons between studies and complicating the interpretation and translation of the preclinical evidence. Future investigations should adopt standardized experimental protocols and comprehensive reporting in accordance with established guidelines to improve reproducibility, comparability, and translational relevance.

An additional translational consideration is that many experimental studies administer purified sulforaphane at pharmacological doses that exceed those likely to be achieved through habitual dietary consumption of cruciferous vegetables or currently available broccoli-derived nutritional supplements. Furthermore, although oral administration (dietary supplementation or oral gavage) more closely reflects the intended route of human exposure, several studies employed intraperitoneal or subcutaneous administration, which bypasses gastrointestinal absorption, glucoraphanin conversion by myrosinase, first-pass metabolism, and the influence of the gut microbiota on sulforaphane bioavailability. While these experimental approaches are valuable for elucidating biological mechanisms and establishing proof-of-concept, they do not fully reproduce the pharmacokinetic profile or systemic exposure associated with dietary intake or oral supplementation and should therefore be interpreted cautiously when considering their translational relevance to human nutrition.

Another important limitation is the predominance of male animal models throughout the preclinical literature. Because sex differences influence adipose tissue biology, brown adipose tissue activity, mitochondrial function, inflammatory responses, insulin sensitivity, hepatic lipid metabolism, gut microbiota composition, and neuroendocrine regulation of energy balance, findings obtained predominantly in male animals cannot be assumed to apply equally to females. Given the recognized role of estrogens and other sex hormones in regulating energy homeostasis and susceptibility to obesity-related metabolic dysfunction, future studies should systematically include both male and female animals and evaluate sex-specific responses to sulforaphane. Such an approach would improve the reproducibility, clinical relevance, and translational value of the preclinical evidence while supporting the development of more personalized therapeutic strategies. In addition, much of the available evidence focuses on relatively early stages of obesity and metabolic dysfunction, whereas comparatively few studies have investigated advanced outcomes such as severe fibrosis, cirrhosis, cardiovascular complications, or long-term metabolic morbidity.

Taken together, the available animal evidence provides strong biological plausibility for the therapeutic potential of sulforaphane in obesity while also highlighting the complexity of its mechanisms of action. Future research should prioritize the standardization of experimental protocols, including sulforaphane dose selection, route and frequency of administration, treatment duration, timing of intervention, and experimental outcome measures, to improve reproducibility and facilitate direct comparisons across studies. Particular emphasis should be placed on studies employing clinically relevant oral dosing regimens and exposure levels that more closely approximate those achievable through dietary intake or nutritional supplementation, thereby strengthening the translational relevance of the preclinical evidence. Additional efforts should focus on optimizing formulations and dosing strategies, enhancing sulforaphane bioavailability, identifying pharmacokinetic and pharmacodynamic biomarkers of treatment response, and clarifying interactions with the gut microbiota and neuroendocrine pathways. Greater emphasis should also be placed on investigating sex-specific responses, long-term efficacy, and advanced obesity-associated complications to strengthen the translational value of the preclinical evidence. Ultimately, well-designed randomized clinical trials are required to determine whether the robust metabolic benefits consistently observed in animal models can be translated into safe and effective strategies for the prevention and management of obesity and obesity-associated metabolic dysfunction in humans.

## 4. Evidence from Human Clinical Studies

### 4.1. Clinical Efficacy of Sulforaphane and Broccoli-Related Preparations

Human clinical evidence evaluating the effects of sulforaphane and broccoli-derived preparations in obesity remains considerably more limited than the extensive body of mechanistic and preclinical research. Importantly, direct clinical evidence investigating obesity-related endpoints, such as body weight, adiposity, body composition, or energy balance, is relatively scarce. Instead, the majority of randomized clinical trials have primarily evaluated obesity-associated metabolic abnormalities, including impaired glucose homeostasis, insulin resistance, type 2 diabetes mellitus (T2DM), prediabetes, MASLD, cardiovascular risk factors, oxidative stress, and chronic inflammation. Consequently, the available clinical literature provides substantially stronger evidence for improvements in obesity-associated metabolic dysfunction than for clinically meaningful reductions in body weight or adiposity [94,95,96,97,98,99,100,101,102,103].

Accordingly, the clinical evidence may be broadly classified into two complementary categories: (i) direct obesity-related evidence, including studies evaluating body weight, adiposity, body composition, and related anthropometric outcomes, and (ii) supportive mechanistic clinical evidence, comprising studies demonstrating improvements in glycemic control, insulin sensitivity, oxidative stress, inflammation, hepatic function, endothelial function, and other metabolic abnormalities frequently associated with obesity. Although these mechanistic improvements provide important biological support for the therapeutic potential of sulforaphane, they should not be interpreted as direct evidence of anti-obesity efficacy. Furthermore, considerable heterogeneity exists among clinical studies with respect to participant characteristics, intervention formulations, sulforaphane dose, treatment duration, bioavailability, and primary outcome measures, thereby limiting direct comparisons and the strength of current conclusions. Table 3 summarizes the available clinical studies according to these complementary categories.

Overall, evidence demonstrating clinically meaningful reductions in body weight or adiposity remains limited and inconsistent. Most randomized controlled trials were not specifically designed or statistically powered to detect changes in body weight or body composition, as these outcomes were generally secondary or exploratory endpoints rather than the primary objectives of the studies. Consequently, the absence of substantial weight loss should not necessarily be interpreted as evidence of a lack of biological activity but rather reflects the design and primary aims of the currently available clinical trials.

The strongest clinical evidence currently available concerns improvements in glucose metabolism and insulin sensitivity. One of the landmark randomized placebo-controlled trials was conducted by Axelsson et al. in patients with T2DM receiving a concentrated broccoli sprout extract providing 150 μmol/day of sulforaphane [94]. Sulforaphane supplementation significantly reduced fasting blood glucose and glycated hemoglobin (HbA1c), with the greatest improvements observed in obese participants with poorly controlled diabetes. Mechanistic analyses further suggested suppression of hepatic gluconeogenesis through Nrf2-dependent downregulation of phosphoenolpyruvate carboxykinase, indicating that the observed metabolic improvements resulted primarily from direct modulation of hepatic glucose metabolism rather than from reductions in body weight [94].

Earlier randomized double-blind clinical trials by Bahadoran et al. similarly demonstrated improvements in several metabolic biomarkers among patients with T2DM. Broccoli sprout powder rich in sulforaphane significantly improved oxidative stress parameters, including total antioxidant capacity, malondialdehyde, oxidized low-density lipoprotein (ox-LDL), and the oxidative stress index [95]. In a related trial, supplementation also reduced fasting blood glucose, fasting insulin, and the homeostasis model assessment of insulin resistance (HOMA-IR), particularly following administration of 10 g/day of broccoli sprout powder [96]. Additional analyses from the same research group further demonstrated reductions in serum triglycerides and the ox-LDL/LDL-cholesterol ratio, suggesting improvements in lipid-related oxidative damage and cardiovascular risk [97]. However, it should be noted that these three studies had overlapping participants. Likewise, Mirmiran et al. reported reductions in high-sensitivity C-reactive protein (hs-CRP) and interleukin-6 (IL-6), whereas TNF-α showed less consistent responses [98]. Collectively, these studies consistently demonstrate improvements in metabolic, oxidative, and inflammatory abnormalities associated with obesity and T2DM, although they were not designed to evaluate weight reduction as a primary endpoint.

More recently, Dwibedi et al. extended these findings to overweight and obese individuals with prediabetes in a 12-week randomized, double-blind, placebo-controlled trial [99]. Broccoli sprout extract produced a modest reduction in fasting blood glucose compared with placebo, although the predefined primary efficacy target was not achieved. Importantly, clinical responses were more pronounced in metabolically defined subgroups and appeared to depend partly on baseline gut microbiota composition, introducing the concept of microbiome-dependent responsiveness to sulforaphane and highlighting the potential role of personalized nutrition approaches [99]. Although this study enrolled overweight and obese participants, its principal outcome remained improvement of glycemic regulation rather than body weight reduction.

Human studies in metabolically healthy individuals further suggest that sulforaphane functions primarily as a modulator of adaptive stress responses rather than as a direct anti-obesity intervention. Van Steenwijk et al. demonstrated that broccoli sprout supplementation modified inflammatory responses during an acute caloric challenge; however, no consistent improvements in metabolic stress biomarkers were observed, indicating that the physiological effects of sulforaphane in healthy individuals are likely subtle and context-dependent [100]. Moreover, the van Steenwijk single—dose study should not be interpreted as evidence for or against weight reduction because it was not designed to evaluate sustained changes in body weight or adiposity.

Several clinical studies have also evaluated obesity-associated hepatic dysfunction and ectopic lipid metabolism. Sulforaphane- or glucoraphanin-rich broccoli preparations have been associated with improvements in hepatic lipid content, liver function, and selected markers of visceral fat accumulation in some populations [101,102]. Nevertheless, these studies generally did not demonstrate clinically meaningful reductions in body weight, and direct assessments of body composition using dual-energy X-ray absorptiometry (DXA), magnetic resonance imaging (MRI), computed tomography (CT), or other validated imaging techniques were rarely performed. Consequently, the effects of sulforaphane on total fat mass, visceral adiposity, lean body mass, and ectopic fat depots remain insufficiently characterized [101,102].

Additional evidence supports the anti-inflammatory properties of broccoli-derived preparations. In overweight adults, daily consumption of broccoli sprouts (30 g/day) for 10 weeks followed by a 10-week washout period resulted in reductions in circulating IL-6 and C-reactive protein (CRP), supporting the capacity of sulforaphane-rich preparations to attenuate low-grade inflammation associated with obesity [103]. However, these improvements occurred in the absence of clinically meaningful anthropometric outcomes and therefore should be interpreted as supportive mechanistic evidence rather than direct evidence of anti-obesity efficacy.

Finally, a bioavailability study in overweight men, premenopausal women, and postmenopausal women demonstrated increased concentrations of metabolites within the glutathione detoxification pathway following five weeks of broccoli sprout consumption, particularly among postmenopausal women [104]. Increased systemic exposure to sulforaphane and sulforaphane-cysteine metabolites confirmed effective absorption and metabolism of broccoli-derived phytochemicals. Nevertheless, because this investigation focused exclusively on pharmacokinetics and bioavailability, it was not designed to evaluate obesity-related clinical outcomes [104].

Collectively, the current clinical evidence indicates that sulforaphane and broccoli-derived preparations consistently improve several metabolic abnormalities commonly associated with obesity, particularly glycemic control, insulin sensitivity, oxidative stress, inflammation, and selected hepatic outcomes. In contrast, evidence supporting clinically meaningful reductions in body weight, adiposity, or body composition remains limited and inconsistent. Therefore, based on the currently available randomized clinical trials, sulforaphane should primarily be regarded as a promising nutritional strategy for improving obesity-associated metabolic dysfunction rather than as an evidence-based weight-loss intervention. Larger, longer-duration randomized controlled trials specifically designed with obesity-related endpoints—including body weight, body composition, visceral adiposity, and energy expenditure—as primary outcomes are required to determine its true clinical efficacy in obesity management.

### 4.2. Critical Appraisal of the Current Clinical Evidence and Translational Challenges

Although the available human clinical studies provide encouraging evidence regarding the metabolic effects of sulforaphane and broccoli-derived preparations, the overall level of clinical evidence remains considerably weaker than the extensive mechanistic and preclinical literature. Collectively, randomized clinical trials consistently demonstrate improvements in glycemic control, insulin sensitivity, oxidative stress, chronic inflammation, endothelial function, hepatic glucose metabolism, and selected lipid-related biomarkers. In contrast, evidence supporting clinically meaningful reductions in body weight, adiposity, waist circumference, or body composition remains limited and inconsistent [94,95,96,97,98,99,100,101,102,103]. Importantly, most clinical trials were designed primarily to evaluate metabolic biomarkers rather than obesity itself, with anthropometric outcomes generally serving as secondary or exploratory endpoints. Consequently, the current human evidence is more appropriately interpreted as supporting beneficial effects on obesity-associated metabolic dysfunction rather than establishing sulforaphane as an effective stand-alone intervention for weight loss or obesity treatment [94,95,96,97,98,99,100,101,102,103].

The discrepancy between the robust anti-obesity effects consistently observed in cellular and animal studies and the comparatively modest findings reported in humans likely reflects multiple biological and methodological factors. Experimental studies typically administer relatively high sulforaphane doses under tightly controlled conditions and frequently initiate treatment before or during the development of obesity. In contrast, clinical studies generally employ lower achievable doses, relatively short intervention periods ranging from acute administration to approximately 12 weeks, and heterogeneous populations with varying degrees of obesity, insulin resistance, prediabetes, T2DM, metabolic syndrome, MASLD, or other metabolic disorders. Furthermore, human obesity is a multifactorial chronic disease influenced by genetic, environmental, dietary, behavioral, socioeconomic, and lifestyle factors that cannot be fully reproduced in experimental models. Collectively, these differences likely contribute to the important translational gap between the highly consistent preclinical findings and the more modest clinical outcomes reported to date [94,95,96,97,98,99,100,101,102,103,104].

Another major challenge in interpreting the available clinical literature is the substantial heterogeneity of the broccoli-derived interventions evaluated across studies. Purified sulforaphane, glucoraphanin-rich preparations, broccoli sprouts, broccoli seed extracts, broccoli powders, and myrosinase-containing formulations should not be considered equivalent interventions because they differ considerably in glucoraphanin content, active sulforaphane concentration, myrosinase activity, formulation characteristics, conversion efficiency, bioavailability, and ultimately systemic sulforaphane exposure. Following ingestion, glucoraphanin may be converted into sulforaphane either by endogenous plant myrosinase or by myrosinase-like enzymes produced by the intestinal microbiota [18,105]. Consequently, systemic sulforaphane exposure varies substantially according to formulation characteristics, food processing, myrosinase activity, gastrointestinal physiology, dietary habits, and gut microbiota composition [18,105]. Furthermore, many primary clinical studies did not fully characterize these formulation-specific parameters or measure biomarkers of systemic sulforaphane exposure. As a result, direct comparisons among studies remain difficult, dose–response relationships cannot be reliably established, and the observed heterogeneity of the clinical findings is likely explained, at least in part, by differences in intervention composition and bioavailability [94,99,104]. These limitations underscore the importance of comprehensive formulation characterization and pharmacokinetic assessment in future clinical investigations.

Recent evidence further indicates that interindividual differences in gut microbiota composition may significantly influence the metabolic efficacy of sulforaphane. The responder analyses reported by Dwibedi et al. demonstrated that baseline gut microbial composition partly determined the glycemic response to broccoli sprout supplementation, introducing the concept of microbiome-dependent responsiveness and supporting future personalized nutrition approaches using sulforaphane-rich interventions [99]. These findings further emphasize that host-specific biological factors should be considered when interpreting clinical outcomes and designing future intervention studies.

Another important limitation of the current literature is that most randomized clinical trials enrolled participants with prediabetes, type 2 diabetes mellitus, metabolic syndrome, MASLD, or other obesity-associated metabolic disorders rather than individuals with uncomplicated obesity. Consequently, improvements in glycemic regulation, oxidative stress, inflammation, endothelial function, lipid metabolism, and hepatic function should be interpreted as evidence supporting improvements in obesity-associated metabolic dysfunction rather than as direct demonstrations of anti-obesity efficacy [94,95,96,97,98,99,100,101,102,103]. Likewise, although reductions in inflammatory mediators, oxidative stress biomarkers, and lipid peroxidation are biologically meaningful because these pathways contribute to adipose tissue dysfunction, insulin resistance, endothelial dysfunction, and cardiometabolic disease progression, improvements in these surrogate metabolic biomarkers should not be interpreted as evidence of clinically meaningful reductions in body weight, adiposity, obesity-related morbidity, or long-term clinical outcomes [94,95,96,97,98,99,100,101,102,103].

Nevertheless, the remarkable consistency of mechanistic observations across in vitro studies, animal models, and, to a lesser extent, human clinical investigations substantially strengthens the biological plausibility of sulforaphane as a multi-target metabolic regulator. Activation of Nrf2-dependent cytoprotective signaling, attenuation of oxidative stress and chronic inflammation, improvement of mitochondrial function, enhancement of insulin sensitivity, modulation of hepatic glucose metabolism, and regulation of lipid homeostasis have now been demonstrated across multiple experimental systems and, to varying degrees, in human studies [21,22,23,24,25,26,27,28,29,30,31,32,33,34,35,36,37,38,39,40,41,42,43,44,45,46,47,48,49,50,51,52,53,54,55,56,57,58,59,60,61,62,63,64,65,66,67,68,69,70,71,72,73,74,75,76,77,78,79,80,81,82,83,84,85,86,87,88,89,90,91,92,93,94,95,96,97,98,99,100,101,102,103,104,105]. This convergence of evidence provides a coherent mechanistic framework supporting the observed metabolic benefits of sulforaphane. However, despite these encouraging biological effects, their translation into clinically meaningful reductions in obesity-related anthropometric outcomes remains insufficiently established [94,95,96,97,98,99,100,101,102,103].

Overall, the current body of clinical evidence suggests that sulforaphane and broccoli-derived preparations should primarily be regarded as promising complementary nutritional interventions for improving obesity-associated metabolic dysfunction rather than as evidence-based therapies for inducing clinically meaningful weight loss. Their principal clinical value appears to lie in improving glycemic regulation, insulin sensitivity, oxidative stress, inflammation, endothelial function, hepatic metabolism, and other metabolic abnormalities that frequently accompany obesity, thereby complementing lifestyle modification and established pharmacological therapies [94,95,96,97,98,99,100,101,102,103]. However, based on the currently available human evidence, sulforaphane cannot yet be considered an effective stand-alone weight-loss intervention, and definitive conclusions regarding its efficacy for obesity treatment cannot presently be drawn.

Future research should prioritize adequately powered, long-term randomized controlled trials specifically designed to evaluate obesity-related outcomes as primary endpoints, including body weight, body composition, visceral adiposity, energy expenditure, and metabolic flexibility. Future studies should also employ well-characterized and standardized sulforaphane formulations, comprehensively reporting glucoraphanin and sulforaphane content, myrosinase activity, expected or measured sulforaphane yield, biomarkers of systemic sulforaphane exposure, pharmacokinetic parameters, and formulation characteristics. In addition, objective body composition techniques (e.g., DXA, MRI, or CT), gut microbiota profiling, and stratified analyses according to baseline metabolic phenotype should be incorporated to improve comparability across studies and facilitate interpretation of dose–response relationships [18,99,104,105]. Such studies will be essential to determine whether the compelling mechanistic and preclinical evidence can ultimately be translated into clinically meaningful benefits for obesity prevention and management.

### 4.3. Gut Microbiota as an Emerging Determinant of Sulforaphane Bioavailability and Clinical Responsiveness

Growing evidence indicates that the gut microbiota is an important determinant of the bioavailability, metabolism, and clinical responsiveness of sulforaphane and broccoli-derived preparations. Sulforaphane is generated from its precursor glucoraphanin through hydrolysis catalyzed either by endogenous plant myrosinase or by myrosinase-like enzymes produced by intestinal microorganisms [18,105]. Consequently, interindividual differences in gut microbial composition may substantially influence the efficiency of glucoraphanin conversion, resulting in considerable variability in systemic sulforaphane exposure and, ultimately, biological activity [18,69,70,71,72,73,105].

Beyond facilitating glucoraphanin conversion, the gut microbiota appears to regulate multiple processes involved in sulforaphane disposition, including intestinal absorption, biotransformation, enterohepatic circulation, and interactions with host metabolic and immune pathways [18,69,70,71,72,73]. In addition, host-related factors such as habitual diet, medication use, obesity-associated dysbiosis, genetic polymorphisms affecting phase II detoxification enzymes, age, and baseline metabolic status may further contribute to interindividual differences in sulforaphane bioavailability and therapeutic responsiveness [18,70,71,72,73,105]. These observations suggest that the metabolic effects of sulforaphane are determined by a complex interplay between the administered preparation and host-specific biological characteristics rather than by the administered dose alone.

As discussed in Section 3, accumulating experimental evidence indicates that the interaction between sulforaphane and the gut microbiota is bidirectional. In addition to facilitating glucoraphanin conversion, sulforaphane and broccoli-derived phytochemicals may themselves modulate gut microbial composition and metabolic activity, thereby improving intestinal barrier integrity, regulating immune homeostasis, promoting short-chain fatty acid production, modulating bile acid metabolism, and attenuating chronic low-grade inflammation [69,70,71,72,73]. These microbiota-mediated mechanisms have been associated with improvements in insulin sensitivity, lipid metabolism, mitochondrial function, energy homeostasis, and systemic metabolic regulation in experimental models [55,56,57,58,59,60,61,62,63,64,65,66,67,68,69,70,71,72,73]. Although these findings cannot yet be directly extrapolated to humans, they provide a coherent biological framework supporting the pleiotropic metabolic effects of sulforaphane observed in preclinical studies.

Emerging clinical evidence further supports the potential translational relevance of these mechanisms. In a recent randomized controlled trial, Dwibedi et al. demonstrated that improvements in fasting blood glucose following broccoli sprout extract supplementation occurred predominantly in participants with specific baseline gut microbiota profiles, suggesting that microbial composition may influence metabolic responsiveness to sulforaphane-rich interventions [99]. Although these findings require confirmation in larger and more diverse populations, they provide preliminary clinical evidence that interindividual variation in gut microbiota composition may contribute to the heterogeneous metabolic responses observed across human intervention studies.

Collectively, the available evidence indicates that gut microbiota composition represents an important source of interindividual variability in the metabolic effects of sulforaphane rather than a definitive explanation for the inconsistent findings reported in clinical trials [69,70,71,72,73,99]. Integrating gut microbiota analyses with pharmacokinetic assessments, biomarkers of sulforaphane exposure, dietary assessment, and metabolic phenotyping may therefore improve our understanding of treatment responsiveness and facilitate the identification of individuals most likely to benefit from sulforaphane-rich interventions. Such approaches are consistent with the emerging concept of precision nutrition and may ultimately contribute to the development of more effective, personalized nutritional strategies for obesity and obesity-associated metabolic dysfunction [18,99,105].

### 4.4. Clinical Safety, Tolerability, and Translational Considerations

The available clinical evidence indicates that sulforaphane and broccoli-derived preparations are generally well tolerated during short-term administration. A comprehensive review by Saito et al., which evaluated 84 registered clinical trials (39 published), concluded that sulforaphane-containing interventions were generally well tolerated across diverse populations, including healthy individuals and patients with obesity, T2DM, prediabetes, liver dysfunction, neurological disorders, and cancer [12]. Reported adverse events were predominantly mild, transient, and gastrointestinal in nature, including bloating, abdominal discomfort, nausea, and alterations in bowel habits, whereas serious treatment-related adverse events were uncommon [12]. Similarly, Fahey et al. concluded that broccoli sprout preparations have been administered safely in numerous human studies, with tolerability being influenced primarily by formulation, dose, and bioavailability rather than intrinsic toxicity [15]. Discontinuation due to adverse events has been rare, supporting the generally acceptable short-term tolerability of sulforaphane-containing interventions [12,15]. Nevertheless, interpretation of the available safety data should be made cautiously because most clinical studies have been of relatively short duration, have enrolled limited numbers of participants, and have evaluated heterogeneous broccoli-derived formulations. Consequently, the long-term safety of chronic supplementation, particularly with higher pharmacological doses of sulforaphane or glucoraphanin, remains insufficiently characterized [12,15]. Furthermore, no tolerable upper intake level has yet been established for sulforaphane or glucoraphanin in humans [12,15]. In addition, substantial variability in glucoraphanin content, myrosinase activity, formulation characteristics, and systemic sulforaphane exposure further complicates interpretation of both efficacy and long-term safety across clinical studies.

Despite the generally acceptable short-term tolerability reported in clinical studies, sulforaphane has the potential to influence xenobiotic metabolism through activation of the Keap1–Nrf2 signaling pathway and the subsequent induction of phase II detoxification enzymes involved in antioxidant defense and cellular detoxification [12,15,106]. Although these biological effects are generally considered beneficial, they also raise the theoretical possibility of altered metabolism or disposition of concomitantly administered medications. Experimental studies further suggest that sulforaphane may modulate the activity of selected phase I enzymes and membrane transporters, thereby influencing xenobiotic handling beyond Nrf2 activation alone [106,107]. However, clinically significant drug–nutrient interactions have not been consistently demonstrated in randomized clinical trials evaluating broccoli-derived preparations [12,15]. Nevertheless, caution may be warranted in individuals receiving medications with a narrow therapeutic index or drugs that depend extensively on phase II conjugation pathways until additional long-term clinical evidence becomes available.

As discussed in Section 4.2 and Section 4.3, considerable interindividual variability remains an important challenge for the clinical translation of sulforaphane. Differences in gut microbiota composition may substantially influence the microbial conversion of glucoraphanin into bioactive sulforaphane, thereby affecting systemic exposure and biological activity [18,99,105]. In addition, genetic polymorphisms in glutathione S-transferase genes, particularly GSTM1, GSTT1, and GSTP1, have been associated with differences in sulforaphane conjugation, pharmacokinetics, and biological responsiveness [15,18,105,108]. Other host-related factors, including age, sex, obesity status, hepatic and renal function, dietary habits, intestinal permeability, and baseline oxidative stress or inflammatory burden, may further contribute to variability in treatment response. Collectively, these findings emphasize the importance of considering individual biological characteristics when interpreting both efficacy and safety outcomes and underscore the need for standardized assessment of systemic sulforaphane exposure in future intervention studies.

Although the available clinical literature indicates generally acceptable short-term tolerability of sulforaphane and broccoli-derived preparations, several important knowledge gaps remain before their long-term safety profile can be adequately characterized. Most intervention studies have enrolled relatively small numbers of participants, have been of limited duration, and have focused primarily on common gastrointestinal symptoms, with comparatively limited evaluation of rare, delayed, cumulative, or dose-dependent adverse effects [12,15]. Future randomized controlled trials should therefore incorporate standardized and comprehensive safety monitoring, including systematic reporting of adverse events, evaluation of liver and kidney function, hematological and endocrine parameters, and careful assessment of potential drug–nutrient interactions, particularly in individuals receiving long-term pharmacotherapy [12,15]. In addition, future studies should routinely quantify circulating and urinary biomarkers of sulforaphane exposure, such as dithiocarbamate metabolites, together with biomarkers of Nrf2 activation, to better define dose–exposure–response relationships, characterize interindividual pharmacokinetic variability, and improve interpretation of both efficacy and long-term safety [18,105]. Finally, greater standardization of sulforaphane-containing preparations with respect to glucoraphanin content, myrosinase activity, formulation, bioavailability, and manufacturing quality will be essential to improve reproducibility across clinical studies and facilitate more reliable evaluation of the efficacy and long-term safety of sulforaphane-containing interventions before their broader clinical application in obesity management and other metabolic disorders [12,15,18,105].

## 5. Gut Microbiota as a Potential Mediator of the Metabolic Effects and Clinical Responsiveness of Sulforaphane

Growing evidence suggests that modulation of the gut microbiota may represent an important mechanism contributing to the metabolic effects and clinical responsiveness of sulforaphane and broccoli-derived preparations in obesity and obesity-associated metabolic dysfunction. Over the past decade, the gut microbiome has emerged as a key regulator of host metabolism, energy homeostasis, immune function, intestinal barrier integrity, and inflammatory responses. Alterations in gut microbial composition, commonly referred to as dysbiosis, have been consistently associated with obesity, insulin resistance, metabolic syndrome, and MASLD [109,110]. Consequently, interventions capable of restoring microbial homeostasis have attracted considerable attention as complementary strategies for improving obesity-associated metabolic dysfunction.

**Figure 3:**
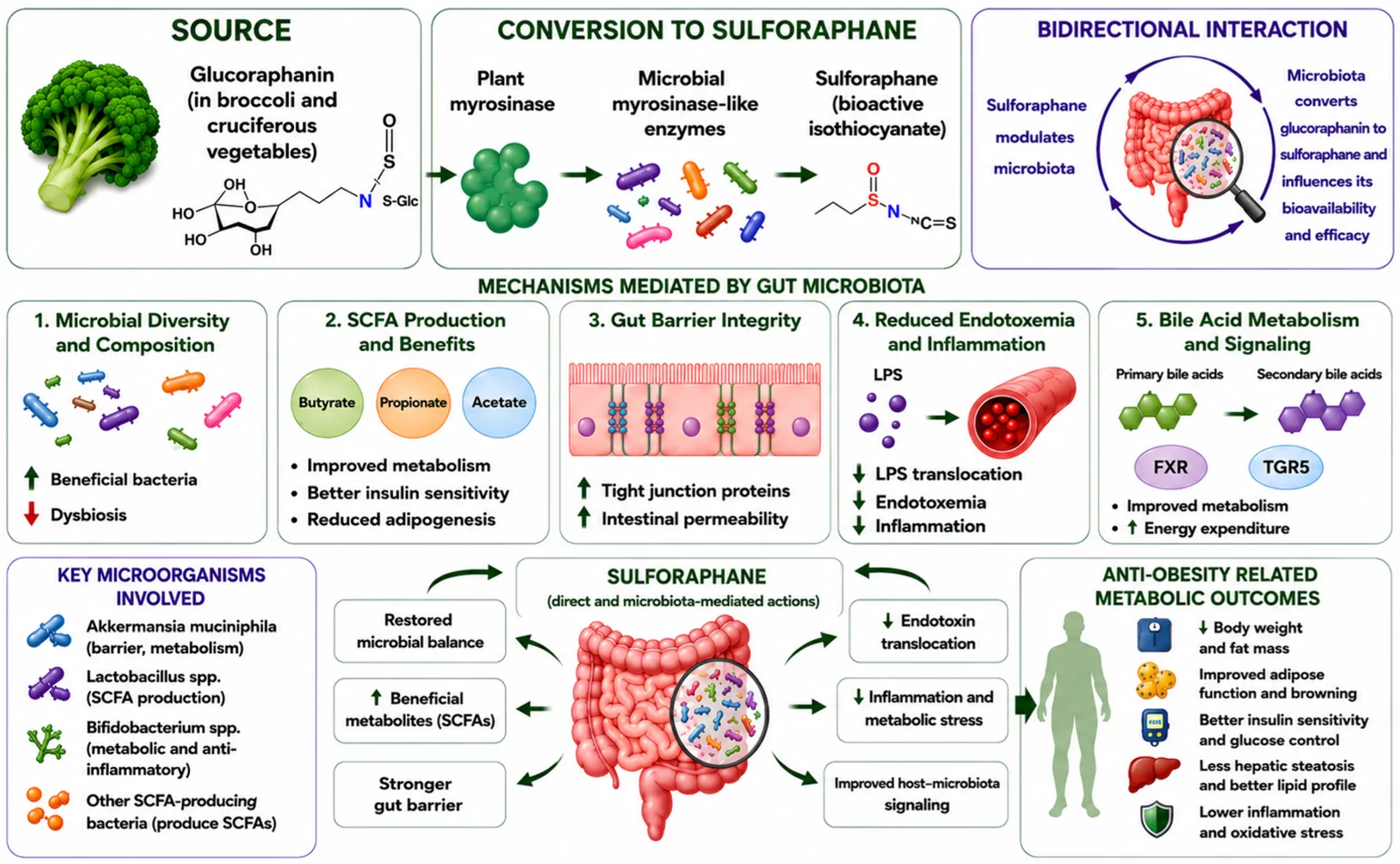
Proposed bidirectional interactions between sulforaphane and the gut microbiota and their potential contribution to obesity-associated metabolic dysfunction. The figure was created by the authors based on the evidence summarized in Refs. [13,18,69,76,90,99,111].

Although research in this field remains relatively recent, both experimental and limited clinical evidence suggest that sulforaphane and glucoraphanin-rich broccoli preparations may beneficially influence gut microbial communities and host–microbiota interactions [18,90,99]. Experimental evidence further suggests that these effects may involve modulation of microbial diversity, enhancement of intestinal barrier integrity, attenuation of metabolic endotoxemia, and suppression of obesity-associated inflammation [69,76,111]. Importantly, the relationship between sulforaphane and the gut microbiota appears to be bidirectional. While sulforaphane may influence microbial composition and function, intestinal microorganisms also contribute to the conversion of glucoraphanin into bioactive sulforaphane, thereby influencing its bioavailability and biological activity [13,18,90,99]. As discussed in Section 4, interindividual differences in gut microbiota composition may also contribute to the heterogeneous metabolic responses observed across clinical intervention studies, although current human evidence remains preliminary [99]. Nevertheless, despite increasing interest in this field, much of the available evidence remains preclinical, and many proposed microbiota-mediated mechanisms have yet to be conclusively demonstrated in humans. Consequently, the precise contribution of gut microbiota modulation to the clinical efficacy of sulforaphane in obesity remains to be established. Figure 3 summarizes the bidirectional interactions between sulforaphane and the gut microbiota and their potential contribution to obesity-associated metabolic dysfunction.

### 5.1. Sulforaphane–Microbiota Interactions

Sulforaphane is derived from glucoraphanin, a glucosinolate abundant in broccoli sprouts and other cruciferous vegetables. Under normal circumstances, conversion of glucoraphanin to sulforaphane is catalyzed by the plant enzyme myrosinase. However, food processing, cooking, and storage frequently reduce myrosinase activity, increasing reliance on intestinal microorganisms for glucoraphanin metabolism [10,16]. Numerous bacterial species possess myrosinase-like enzymatic activity capable of converting glucoraphanin into sulforaphane and related isothiocyanates.

This microbial contribution introduces substantial interindividual variability in sulforaphane bioavailability. Differences in gut microbiota composition may partially explain why individuals exhibit variable responses to broccoli-derived interventions despite consuming similar amounts of glucoraphanin. Emerging evidence suggests that bacterial genera including Bacteroides, Lactobacillus, Enterococcus, Bifidobacterium, and certain Clostridium species may participate in glucosinolate metabolism [13,18,99]. These observations are consistent with the clinical findings discussed in Section 4, where differences in baseline gut microbiota composition were associated with variable metabolic responses to broccoli sprout supplementation [99]. However, the specific microbial enzymes and metabolic pathways responsible for sulforaphane production remain incompletely characterized. Consequently, considerable uncertainty persists regarding the extent to which individual microbiota profiles determine systemic sulforaphane exposure, metabolic responsiveness, and ultimately clinical efficacy.

In addition to being metabolized by gut microorganisms, sulforaphane itself may influence microbial community structure and function. Experimental studies suggest that sulforaphane can modulate bacterial growth, microbial metabolic activity, and host–microbiota interactions [16,18,112], thereby establishing a potentially reciprocal relationship between sulforaphane exposure and microbial ecology. Nevertheless, many of the reported associations remain observational, and direct causal evidence linking specific microbiota alterations to improvements in obesity-associated metabolic dysfunction remains limited. Accordingly, further mechanistic investigations and well-designed human intervention studies integrating microbiome profiling with pharmacokinetic and clinical outcome assessments are required to clarify the role of gut microbiota in mediating the metabolic effects and clinical responsiveness of sulforaphane.

### 5.2. Effects on Microbial Diversity

Reduced microbial diversity is frequently observed in obesity and has been associated with impaired metabolic flexibility, increased inflammation, and greater susceptibility to metabolic disease [113]. Several animal studies have demonstrated that sulforaphane and broccoli-derived preparations can partially reverse obesity-associated dysbiosis and increase microbial diversity [68,69,89,90].

Experimental studies have shown that sulforaphane supplementation is associated with favorable alterations in the relative abundance of bacterial taxa involved in short-chain fatty acid (SCFA) production, bile acid metabolism, and maintenance of intestinal homeostasis [69,90,112,114,115]. In particular, increases in beneficial microbial groups such as *Lactobacillus*, *Bifidobacterium*, *Akkermansia muciniphila*, and other SCFA-producing microorganisms have been consistently reported in experimental models. These microbial alterations may contribute to improvements in glucose metabolism, adipose tissue function, and systemic inflammation, thereby providing a biologically plausible mechanism linking gut microbiota modulation with the metabolic effects of sulforaphane [69,90,112,114,115].

Among these microorganisms, *Akkermansia muciniphila* has attracted particular attention because of its consistent association with improved metabolic health, enhanced insulin sensitivity, preservation of intestinal barrier integrity, and reduced adiposity in both experimental models and human observational studies [116,117]. Nevertheless, caution is warranted when interpreting these findings. In many studies, alterations in microbial abundance are based primarily on association analyses rather than mechanistic investigations demonstrating direct causal relationships. Furthermore, changes in microbial composition do not necessarily reflect corresponding alterations in microbial function, metabolite production, or host–microbe interactions. Human intervention studies remain limited, and substantial methodological variability among microbiome studies—including differences in sequencing methodologies, analytical pipelines, and dietary interventions—further complicates interpretation. Consequently, although modulation of gut microbial diversity represents a plausible mechanism underlying the metabolic effects of sulforaphane, current evidence does not yet establish that these microbial changes directly mediate the beneficial metabolic responses observed in human clinical studies.

### 5.3. Gut Barrier Function

Impairment of intestinal barrier integrity is increasingly recognized as an important contributor to obesity-associated metabolic dysfunction. Disruption of tight junction proteins increases intestinal permeability, allowing translocation of bacterial products from the intestinal lumen into the systemic circulation. This process promotes chronic low-grade inflammation and contributes to insulin resistance and the progression of obesity-related metabolic disorders [118,119].

Several experimental studies indicate that sulforaphane may improve gut barrier function through multiple complementary mechanisms [75,120,121,122,123]. Activation of the Nrf2 signaling pathway enhances endogenous antioxidant defenses and protects intestinal epithelial cells against oxidative injury. Experimental studies further suggest that sulforaphane preserves the expression and organization of tight junction proteins, including occludin, claudins, and zonula occludens-1 (ZO-1), thereby helping to maintain epithelial barrier integrity. In addition, sulforaphane has been shown in experimental models to attenuate intestinal inflammatory signaling pathways, particularly TLR4/MyD88/NF-κB and MAPK signaling, while simultaneously modulating gut microbial communities and reducing oxidative stress [75,120,121,122,123].

By attenuating oxidative stress and intestinal inflammation, sulforaphane may protect the intestinal epithelium against obesity-associated injury [75,82,123,124]. Experimental evidence further suggests that improved epithelial barrier integrity may reduce the translocation of luminal antigens, bacterial products, and endotoxins into the systemic circulation, thereby potentially limiting activation of systemic inflammatory pathways associated with insulin resistance and metabolic dysfunction [118,125]. These observations provide a biologically plausible mechanism linking improvements in gut barrier integrity with the favorable metabolic effects reported in experimental obesity models. However, evidence supporting improvements in intestinal permeability is derived predominantly from animal studies. Direct assessment of gut barrier function has been performed only rarely in human intervention trials, and therefore the contribution of improved intestinal barrier integrity to the clinical efficacy of sulforaphane remains uncertain. Future randomized controlled trials incorporating validated biomarkers of intestinal permeability together with metabolic outcomes will be required to determine whether these promising mechanistic observations translate into clinically meaningful benefits in individuals with obesity and obesity-associated metabolic dysfunction.

### 5.4. Inflammation and Endotoxemia

Metabolic endotoxemia has emerged as a key pathogenic mechanism linking obesity-associated dysbiosis with systemic inflammation and metabolic dysfunction [126,127]. Increased intestinal permeability allows LPS, a component of the outer membrane of Gram-negative bacteria, to translocate from the intestinal lumen into the systemic circulation. Elevated circulating LPS concentrations activate TLR4 signaling, thereby promoting chronic low-grade inflammation, insulin resistance, adipose tissue dysfunction, and hepatic steatosis [126,127].

A growing body of experimental evidence suggests that sulforaphane may attenuate metabolic endotoxemia through complementary mechanisms, including modulation of gut microbiota composition, preservation of intestinal barrier integrity, and suppression of inflammatory signaling pathways [128,129]. Experimental studies have consistently demonstrated reductions in circulating LPS concentrations, decreased expression of pro-inflammatory cytokines, and attenuation of TLR4/NF-κB signaling following sulforaphane administration [13,128,129]. These findings are of particular interest because chronic low-grade inflammation is widely recognized as a central feature of obesity and a major contributor to the development of obesity-associated metabolic complications.

However, an important unresolved question is whether these beneficial effects are mediated primarily through alterations in the gut microbiota or instead reflect the direct cellular actions of sulforaphane. Activation of Nrf2 signaling, suppression of NF-κB-mediated inflammation, enhancement of mitochondrial function, and modulation of glucose metabolism have all been demonstrated in multiple tissues independently of microbial mechanisms. Consequently, the relative contribution of microbiota-dependent and microbiota-independent pathways to the overall metabolic effects of sulforaphane remains incompletely understood. Advanced experimental approaches—including germ-free animal models, fecal microbiota transplantation, metabolomics, metagenomics, and integrated multi-omics analyses—will be required to disentangle these complex interactions and determine the extent to which microbiota modulation contributes to the metabolic benefits of sulforaphane [130,131,132].

### 5.5. Concluding Perspective

The gut microbiota has emerged as one of the most promising and rapidly expanding areas of research regarding the metabolic effects and clinical responsiveness of sulforaphane and broccoli-derived preparations in obesity and obesity-associated metabolic dysfunction. Current evidence suggests that sulforaphane may beneficially influence microbial composition, enhance microbial diversity, improve intestinal barrier integrity, reduce metabolic endotoxemia, and attenuate obesity-associated inflammation. Collectively, these mechanisms may complement the direct actions of sulforaphane on adipose tissue, liver, skeletal muscle, and other metabolic signaling pathways, thereby providing a biologically plausible explanation for some of its beneficial metabolic effects.

Nevertheless, the current evidence base is characterized by several important limitations, including the predominance of animal studies, substantial interindividual variability in gut microbiota composition and sulforaphane metabolism, incomplete characterization of microbial glucoraphanin-converting pathways, and considerable methodological heterogeneity across microbiome studies. Therefore, although modulation of the gut microbiota represents one of the most compelling emerging mechanisms contributing to the metabolic effects of sulforaphane, many proposed interactions remain associative rather than definitively causal. At present, available human studies do not allow definitive conclusions regarding the extent to which gut microbiota modulation contributes to the clinical efficacy of sulforaphane, highlighting an important translational knowledge gap.

Future studies integrating comprehensive microbiome profiling, validated biomarkers of intestinal permeability, metabolomics, pharmacokinetic analyses, and well-designed randomized clinical trials will be essential to clarify the role of host–microbiota interactions. Such investigations should determine whether microbiota-mediated responses contribute to the metabolic benefits observed in human intervention studies and whether these mechanisms can be leveraged to optimize the therapeutic potential of sulforaphane as a complementary nutritional strategy for obesity-associated metabolic dysfunction.

## 6. Analyzing the Limitations of the Current Evidence

Despite the growing body of evidence indicating that sulforaphane and broccoli-derived preparations influence multiple biological pathways implicated in obesity and obesity-associated metabolic dysfunction, the overall strength of the available evidence remains uneven across different levels of investigation. The mechanistic evidence generated from cellular studies is extensive and internally consistent, while preclinical animal studies provide important physiological validation under controlled experimental conditions. However, the current clinical literature remains comparatively limited, heterogeneous, and primarily focused on surrogate metabolic endpoints rather than obesity-specific outcomes. Consequently, the certainty of evidence progressively decreases along the translational pathway from mechanistic discovery to clinical application.

A comprehensive assessment of sulforaphane as a potential strategy for obesity management therefore requires not only consideration of the reported biological effects but also critical evaluation of the methodological limitations associated with each level of evidence. Understanding these limitations is essential for distinguishing mechanistic plausibility from preclinical efficacy and ultimately from demonstrated clinical effectiveness. The following sections critically examine the principal strengths and limitations of the available in vitro, animal, and human studies and discuss how these factors influence the interpretation and translational relevance of the current evidence.

### 6.1. Limitations of In Vitro Cellular Studies

In vitro studies constitute the mechanistic foundation of the current evidence base and have substantially advanced our understanding of the molecular actions of sulforaphane in obesity-related metabolic regulation. Experimental models including 3T3-L1 preadipocytes, mature adipocytes, hepatocytes, myotubes, macrophages, and adipocyte–macrophage co-culture systems have consistently demonstrated that sulforaphane modulates multiple pathways implicated in adipogenesis, lipid metabolism, oxidative stress, inflammation, mitochondrial function, thermogenesis, and insulin signaling. Collectively, these studies provide strong evidence supporting the biological plausibility of sulforaphane as a modulator of obesity-associated metabolic dysfunction.

Nevertheless, the translational significance of these findings should be interpreted cautiously. Cell culture models are inherently reductionist and cannot reproduce the complex physiological interactions that characterize obesity in vivo. Whole-body metabolic regulation involves coordinated communication among adipose tissue, skeletal muscle, liver, pancreas, intestine, the immune system, the central nervous system, endocrine signaling, and the gut microbiota. Consequently, molecular responses observed in isolated cells cannot be assumed to accurately predict physiological responses at the organismal level.

Another important limitation concerns the experimental concentrations of sulforaphane employed in many mechanistic studies. Numerous investigations utilize concentrations that exceed those typically achievable following dietary consumption of cruciferous vegetables or currently available nutritional supplementation. Although supraphysiological concentrations are valuable for elucidating molecular mechanisms and identifying potential therapeutic targets, they may exaggerate the magnitude of biological responses and therefore overestimate the physiological relevance of some reported effects. As a result, demonstration of mechanistic activity under experimental conditions should not be interpreted as evidence that comparable effects occur under clinically achievable exposure levels.

Considerable methodological heterogeneity further complicates interpretation of the cellular literature. Differences in cell origin, species, differentiation stage, culture conditions, sulforaphane concentrations, exposure duration, and outcome assessment limit direct comparisons among studies and reduce overall reproducibility. Moreover, many investigations rely on immortalized murine cell lines, which may not fully reproduce the biological behavior of primary human cells or human adipose tissue. Consequently, although the reported molecular effects are remarkably consistent, their quantitative magnitude and physiological relevance remain uncertain.

A further limitation is that conventional cell culture systems do not adequately reflect the pharmacokinetic behavior of sulforaphane in vivo. Following oral administration, sulforaphane undergoes extensive absorption, metabolism through the mercapturic acid pathway, tissue distribution, and elimination, generating multiple metabolites that may contribute to—or differ from—the biological activity of the parent compound. These pharmacokinetic processes, together with factors such as bioavailability and gut microbiota-mediated metabolism, are largely absent from standard in vitro models. Therefore, cellular experiments generally evaluate direct exposure to sulforaphane rather than the dynamic physiological environment encountered following dietary intake or supplementation.

Finally, although mechanistic studies necessarily focus on individual signaling pathways to establish causal relationships, obesity is a multifactorial disorder arising from complex interactions among metabolic, inflammatory, endocrine, neural, genetic, behavioral, environmental, and microbiota-related factors. Consequently, modulation of a single molecular pathway under controlled laboratory conditions does not necessarily translate into clinically meaningful improvements in obesity or its associated metabolic complications.

Taken together, the available in vitro evidence provides compelling mechanistic support for the biological activities of sulforaphane and establishes a strong rationale for further investigation. However, these studies should primarily be regarded as hypothesis-generating and mechanistic, rather than as direct evidence of therapeutic efficacy. Confirmation of these findings therefore depends on their reproducibility in well-designed animal models and, ultimately, in adequately powered randomized clinical trials demonstrating meaningful improvements in obesity-related clinical outcomes.

### 6.2. Limitations of Animal Models

Animal studies represent the essential intermediate step between mechanistic discoveries in cellular systems and clinical evaluation in humans. Collectively, the available preclinical evidence consistently demonstrates that sulforaphane attenuates body weight gain, reduces adiposity, improves insulin sensitivity, suppresses inflammation and oxidative stress, enhances mitochondrial function and thermogenesis, and ameliorates obesity-associated metabolic disturbances in a variety of experimental models. Compared with the cellular literature, these studies provide substantially stronger evidence that the molecular mechanisms identified in vitro translate into physiological benefits within the context of whole-body metabolism.

Nevertheless, although the preclinical evidence is generally consistent and biologically compelling, several important methodological and translational limitations should be considered before extrapolating these findings to human obesity.

The principal limitation relates to the inherent biological differences between animal models and humans. Rodents differ from humans in numerous physiological, metabolic, endocrine, immunological, and microbiological characteristics that influence energy homeostasis, adipose tissue biology, inflammatory responses, and xenobiotic metabolism. Consequently, therapeutic responses observed in experimental animals cannot be assumed to predict equivalent efficacy in humans, particularly for complex multifactorial disorders such as obesity.

The experimental models themselves also have important limitations. Although high-fat diet-induced obesity, genetically obese mice, diabetic models, and other experimental systems successfully reproduce several metabolic features of human obesity, they cannot fully recapitulate its multifactorial etiology. Human obesity develops over many years through the complex interaction of genetic susceptibility, dietary patterns, physical inactivity, psychosocial stress, socioeconomic determinants, environmental exposures, and behavioral factors. In contrast, experimental obesity is typically induced under highly controlled laboratory conditions over relatively short time periods. Consequently, these models provide valuable proof-of-concept but represent simplified biological systems rather than complete models of human disease.

Another important consideration concerns sulforaphane exposure. Many preclinical studies administer doses that substantially exceed those achievable through habitual dietary consumption and, in some cases, employ routes of administration that bypass the normal processes of gastrointestinal absorption, glucoraphanin conversion, first-pass metabolism, and microbiota-mediated biotransformation. Although these approaches are valuable for establishing pharmacological efficacy and elucidating mechanisms of action, they may overestimate the therapeutic potential achievable through dietary intake or currently available nutritional supplementation. Therefore, demonstration of efficacy in animal models should not be interpreted as direct evidence that comparable benefits can be achieved under clinically relevant exposure conditions.

Methodological heterogeneity further complicates interpretation of the preclinical literature. Considerable variation exists among studies with respect to animal strain, obesity model, dietary composition, intervention timing, treatment duration, sulforaphane formulation, dose, route of administration, and outcome assessment. Although the overall direction of the reported findings is remarkably consistent, this heterogeneity limits direct comparison among studies and makes it difficult to identify the optimal intervention strategy or establish clear dose–response relationships.

The relatively short duration of most experimental studies also limits interpretation. Whereas obesity develops and persists over years or decades in humans, many animal experiments evaluate outcomes over only several weeks or months. Consequently, the long-term efficacy, durability, and safety of sulforaphane administration remain insufficiently characterized.

Another emerging consideration is the role of the gut microbiota. Increasing evidence indicates that microbial composition substantially influences glucoraphanin conversion, sulforaphane bioavailability, and metabolic responsiveness. Because the composition and functional capacity of rodent microbiota differ considerably from those of humans, microbiota-dependent effects observed in experimental animals may not accurately predict clinical responses. This limitation is particularly important given the growing recognition that both obesity pathogenesis and sulforaphane metabolism are strongly influenced by host–microbiome interactions.

Finally, publication bias may contribute to an overrepresentation of positive findings within the preclinical literature, as studies reporting neutral or negative outcomes are less likely to be published. Consequently, the overall efficacy of sulforaphane may be overestimated if the available evidence is interpreted without considering this potential source of bias.

Taken together, animal studies provide convincing proof-of-concept that the mechanistic effects identified in cellular models can translate into physiological improvements under controlled experimental conditions. However, they should primarily be viewed as translational evidence supporting biological efficacy rather than definitive evidence of clinical effectiveness. Confirmation of these promising findings ultimately requires adequately powered, well-designed randomized clinical trials evaluating clinically meaningful obesity-related outcomes under standardized intervention conditions.

### 6.3. Limitations of Human Clinical Trials

Human intervention studies represent the highest level of translational evidence and therefore provide the most direct basis for evaluating the clinical relevance of sulforaphane and broccoli-derived preparations. However, despite encouraging findings regarding improvements in insulin sensitivity, glycemic control, inflammation, oxidative stress, endothelial function, and other obesity-associated metabolic abnormalities, the current clinical literature remains comparatively limited. Importantly, most available studies have been conducted in individuals with metabolic syndrome, T2DM, insulin resistance, or related cardiometabolic disorders rather than populations specifically recruited for obesity. Consequently, the available evidence more strongly supports potential benefits for obesity-associated metabolic dysfunction than for obesity itself, and direct evidence demonstrating clinically meaningful anti-obesity efficacy remains limited.

One of the principal limitations is the relatively small size of most published clinical trials. Many studies include fewer than 100 participants, reducing statistical power and increasing the likelihood of both type II errors and imprecise estimates of treatment effects. As a result, modest but clinically relevant changes in body weight, body composition, or adiposity may remain undetected.

The relatively short duration of most interventions represents another important limitation. Because obesity is a chronic disease requiring sustained therapeutic intervention, studies lasting only several weeks or months are unlikely to adequately evaluate long-term effects on body weight regulation, adipose tissue remodeling, visceral fat accumulation, or maintenance of metabolic improvements. Consequently, whether the observed metabolic benefits translate into durable clinical outcomes remains uncertain.

Interpretation of the current clinical evidence is further complicated by substantial heterogeneity among intervention formulations. Published studies have investigated fresh broccoli sprouts, broccoli sprout beverages, broccoli powders, glucoraphanin-rich extracts, purified sulforaphane preparations, and products containing different levels of active myrosinase. These interventions differ markedly with respect to glucoraphanin content, sulforaphane yield, conversion efficiency, bioavailability, and pharmacokinetic behavior. Furthermore, many studies provide limited information regarding formulation composition, myrosinase activity, or biomarkers confirming systemic sulforaphane exposure. Consequently, it remains difficult to determine whether inconsistent clinical findings reflect genuine differences in biological efficacy or simply differences in formulation characteristics and bioavailability.

A related limitation concerns the lack of standardized dosing strategies. Some investigations report administered glucoraphanin content, whereas others report estimated sulforaphane yield or total extract dose. Because conversion of glucoraphanin to sulforaphane depends on factors such as myrosinase activity and gut microbiota composition, identical nominal doses may produce markedly different systemic exposure among participants. As a result, dose–response relationships remain poorly characterized, limiting identification of clinically effective dosing regimens.

Perhaps the most important limitation of the current clinical literature is that relatively few studies have been specifically designed to evaluate obesity-related outcomes. In many trials, body weight, body mass index, waist circumference, body composition, or visceral adiposity constitute secondary or exploratory outcomes rather than primary study endpoints. Instead, investigations predominantly assess surrogate biomarkers of glucose metabolism, oxidative stress, inflammation, or cardiovascular risk. Although these endpoints provide important mechanistic and metabolic information, improvements in surrogate biomarkers should not be interpreted as direct evidence of clinically meaningful reductions in obesity or adiposity.

This limitation is further compounded by the relatively infrequent use of advanced body composition techniques such as DXA, MRI, or CT. Consequently, it remains unclear whether sulforaphane preferentially influences visceral adipose tissue, ectopic fat accumulation, lean body mass, or total body fat distribution.

Interpretation of clinical findings is also complicated by substantial interindividual variability. Factors including gut microbiota composition, genetic polymorphisms affecting xenobiotic metabolism, habitual dietary intake, age, sex, medication use, and baseline metabolic status may all influence sulforaphane absorption, metabolism, and biological responsiveness. However, these potential modifiers have rarely been incorporated into study design or systematically evaluated, limiting understanding of the determinants of clinical response.

Finally, pharmacokinetic assessments remain notably underutilized in clinical intervention studies. Without objective biomarkers confirming systemic sulforaphane exposure, such as urinary dithiocarbamates or circulating metabolites, it is often difficult to distinguish true biological non-responsiveness from inadequate absorption or bioavailability. This limitation substantially complicates interpretation of both positive and negative clinical findings.

Overall, the current human evidence supports the biological and clinical plausibility of sulforaphane as a strategy for improving obesity-associated metabolic dysfunction. However, the available evidence remains insufficient to establish sulforaphane as an evidence-based intervention for obesity itself. Future clinical research should prioritize adequately powered randomized controlled trials using standardized formulations, validated pharmacokinetic biomarkers, clinically relevant obesity-specific endpoints, comprehensive body composition assessment, and longer intervention periods. Only through such rigorous studies will it be possible to determine whether the promising mechanistic and preclinical findings translate into clinically meaningful benefits for individuals living with obesity.

### 6.4. Concluding Perspective

Collectively, the current evidence demonstrates a progressive transition from strong mechanistic plausibility to promising preclinical efficacy, but considerably more limited clinical certainty. Cellular studies consistently show that sulforaphane modulates multiple molecular pathways implicated in obesity pathophysiology, including oxidative stress, inflammation, adipogenesis, mitochondrial function, thermogenesis, and insulin signaling, thereby establishing a robust mechanistic rationale for its potential therapeutic application. Animal studies further support the biological relevance of these mechanisms by demonstrating reproducible improvements in obesity-associated metabolic disturbances under controlled experimental conditions. However, the strength of the evidence decreases as the level of biological complexity increases, reflecting the methodological and translational challenges inherent in moving from experimental models to human disease.

The current clinical literature, although encouraging, remains insufficient to establish definitive conclusions regarding the efficacy of sulforaphane or broccoli-derived preparations as evidence-based interventions for obesity. Most clinical studies have evaluated populations with metabolic syndrome, insulin resistance, or T2DM rather than obesity itself, and have primarily examined surrogate metabolic biomarkers rather than obesity-specific outcomes. Furthermore, substantial heterogeneity in intervention formulations, dosing strategies, treatment duration, pharmacokinetic characterization, and outcome assessment limits cross-study comparability and complicates interpretation of the available evidence. Consequently, improvements in metabolic biomarkers should not be interpreted as definitive evidence of clinically meaningful reductions in body weight, adiposity, or obesity-related disease burden.

Taken together, the available literature supports continued investigation of sulforaphane as a promising dietary bioactive compound with potential applications in the management of obesity-associated metabolic dysfunction. However, the current evidence is more compelling for biological plausibility and mechanistic activity than for demonstrated clinical effectiveness. Accordingly, sulforaphane should presently be regarded as an investigational adjunctive strategy rather than an established therapeutic intervention for obesity.

Future research should therefore focus on bridging the gap between mechanistic discoveries and clinical application through adequately powered, long-term randomized controlled trials using standardized intervention formulations, rigorous pharmacokinetic and pharmacodynamic assessments, validated biomarkers of sulforaphane exposure, comprehensive body composition analyses, and clinically relevant obesity-specific endpoints. Greater consideration should also be given to individual determinants of responsiveness, including gut microbiota composition, genetic variability, dietary patterns, and other precision nutrition approaches. Addressing these methodological and translational challenges will be essential for determining whether the consistently promising mechanistic and preclinical findings ultimately translate into meaningful and reproducible clinical benefits for individuals living with obesity.

## 7. Future Perspectives

Although the available mechanistic and preclinical evidence provides a strong biological rationale for investigating sulforaphane and broccoli-derived preparations in obesity, substantial barriers continue to limit their translation into clinical practice. Future research should therefore move beyond demonstrating biological activity and focus on determining who is most likely to benefit, which formulation and dose provide reproducible systemic exposure, and whether the observed molecular and metabolic effects result in clinically meaningful improvements in adiposity and obesity-related health outcomes. Addressing these questions will require the integration of precision nutrition, multi-omics and systems biology, standardized intervention formulations, pharmacokinetic assessment, and rigorously designed clinical trials.

**Figure 4:**
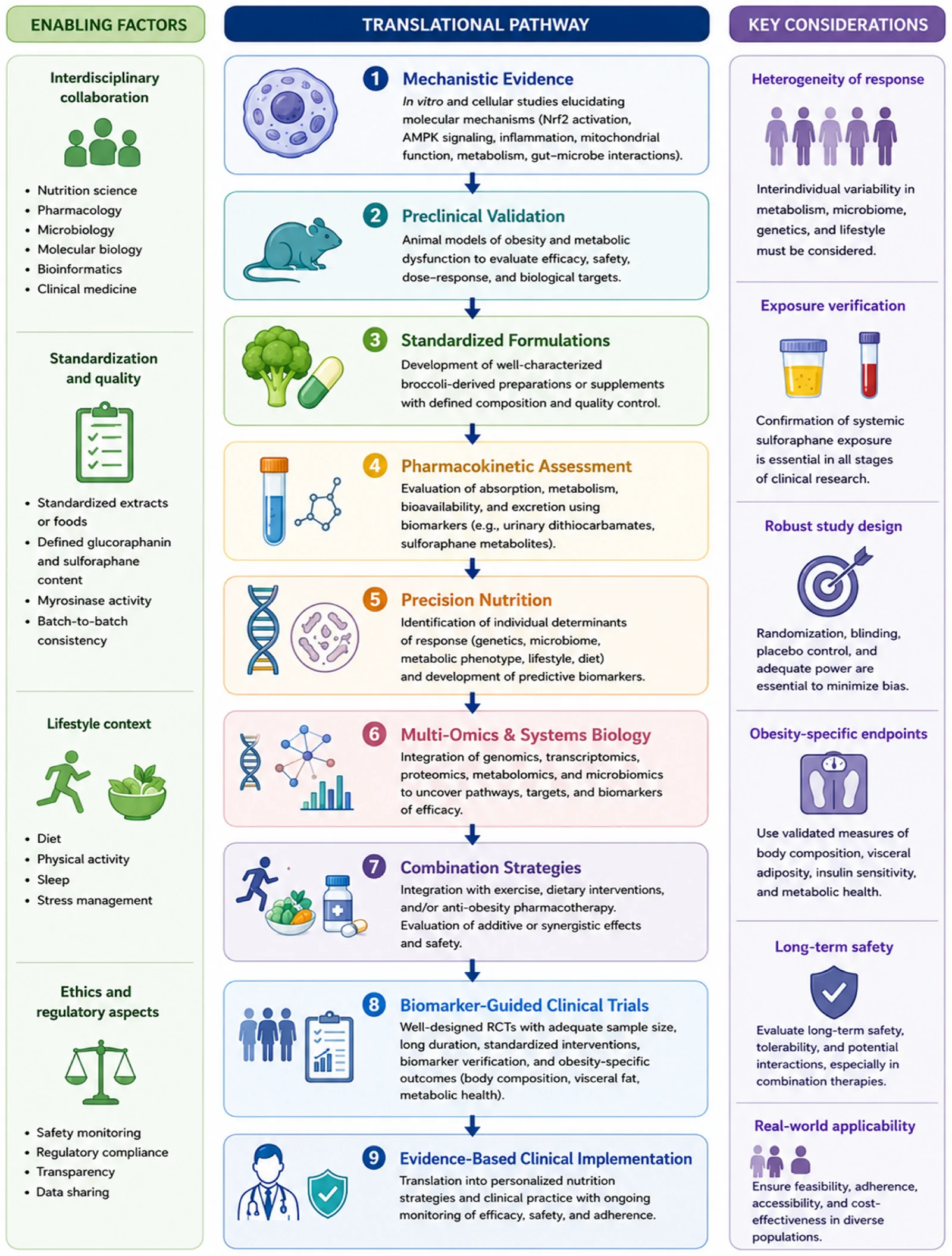
Proposed translational framework for the future clinical development of sulforaphane and broccoli-derived preparations in obesity and obesity-associated metabolic dysfunction. The framework summarizes evidence discussed throughout this review and was developed by the authors based on Refs. [18,22,28,29,30,31,32,33,34,61,72,96,118,130,133,134,135,136,137,138,139,140,141,142,143,144,145,146].

As summarized in Figure 4, the future clinical development of sulforaphane should follow an integrated translational framework linking individual biological characteristics, molecular response profiles, intervention composition, systemic exposure, and clinically relevant outcomes. Within this framework, precision nutrition may help identify responsive population subgroups, multi-omics approaches may clarify the interconnected mechanisms underlying treatment response, and standardized formulations with pharmacokinetic verification may improve reproducibility and cross-study comparability. Together, these strategies may help bridge the current gap between strong mechanistic plausibility, promising preclinical efficacy, and still-limited evidence of clinical effectiveness.

### 7.1. Precision Nutrition Approaches

The substantial interindividual variability observed following dietary and supplemental interventions has increased interest in precision, or personalized, nutrition. Unlike conventional population-level recommendations, precision nutrition seeks to tailor dietary strategies according to an individual’s genetic background, metabolic phenotype, gut microbiota composition, lifestyle characteristics, environmental exposures, and baseline disease status. This approach may be particularly relevant to sulforaphane because both its systemic availability and biological effects are influenced by multiple host- and intervention-related factors.

Sulforaphane is an attractive candidate for precision nutrition applications because its metabolism and biological activity may vary according to gut microbiota composition, genetic polymorphisms affecting xenobiotic-metabolizing and detoxification enzymes, inflammatory and oxidative status, dietary habits, and baseline metabolic health. Consequently, the inconsistent responses observed across existing clinical studies may not necessarily indicate an absence of biological efficacy but may partly reflect differences in systemic exposure and individual responsiveness. Future studies should therefore determine whether specific subgroups derive greater benefits from sulforaphane or broccoli-derived interventions than the general population [133,134]. Individuals with obesity-associated insulin resistance, elevated oxidative stress, chronic low-grade inflammation, impaired metabolic flexibility, or microbiome profiles favoring glucoraphanin conversion may, for example, demonstrate greater responsiveness [133,134]. However, these possibilities remain hypotheses that require prospective testing rather than assumptions based on subgroup observations.

A major research priority is the identification of biomarkers capable of distinguishing responders from non-responders before or during intervention. Potential biomarkers may include urinary or circulating indicators of sulforaphane exposure, glutathione S-transferase genotypes, inflammatory and oxidative stress profiles, insulin-resistance phenotypes, and microbiome-derived measures of glucoraphanin-conversion capacity. Their incorporation into randomized trials could clarify whether variability in outcomes reflects inadequate exposure, differences in biological sensitivity, or both. Predictive and response biomarkers may also improve participant stratification, increase statistical power, and reduce unnecessary supplementation among individuals unlikely to benefit [135,136].

As illustrated in Figure 4, precision nutrition should not be considered an isolated research direction but rather the clinical application of information generated through pharmacokinetic, microbiome, genetic, metabolic, and multi-omics analyses. Integrating these data may ultimately enable sulforaphane-based interventions to be targeted toward individuals with the greatest probability of achieving a meaningful metabolic response. Nevertheless, such implementation will require independently validated biomarkers, prospectively defined responder criteria, and evidence that biomarker-guided strategies provide greater clinical benefit than conventional population-based supplementation.

### 7.2. Multi-Omics and Systems Biology

Obesity is a heterogeneous and multifactorial disease arising from complex interactions among genetic, epigenetic, transcriptomic, proteomic, metabolomic, microbiomic, endocrine, behavioral, and environmental determinants. Similarly, sulforaphane influences several interconnected biological processes rather than acting through a single molecular target. Reductionist studies focusing on individual signaling pathways are therefore valuable for establishing causal mechanisms but are insufficient to characterize the full systemic response to sulforaphane or explain the marked variability observed among individuals.

Multi-omics technologies offer an important means of addressing this complexity. Genomic analyses may identify genetic variants associated with differences in sulforaphane metabolism, detoxification capacity, or metabolic responsiveness. Epigenomic and transcriptomic approaches can characterize changes in gene regulation related to Nrf2 activation, inflammatory suppression, mitochondrial remodeling, lipid metabolism, and insulin signaling. Proteomic analyses may clarify whether transcriptional changes translate into altered protein abundance and pathway activity, whereas metabolomics can identify changes in metabolic intermediates, redox status, lipid profiles, and biochemical fluxes. In parallel, microbiome analyses can characterize the microbial communities and functional pathways involved in glucoraphanin conversion, sulforaphane bioavailability, and downstream metabolic responses [130,137,138].

The greatest value of these technologies is likely to arise from their integration rather than from isolated analyses. Systems biology approaches can combine molecular, microbial, pharmacokinetic, and clinical data to identify response networks that cannot be detected through single-pathway investigations. Such integration may clarify how oxidative stress, inflammation, mitochondrial dysfunction, adipose-tissue remodeling, insulin resistance, and gut microbiota interact during sulforaphane exposure. It may also help identify molecular signatures associated with favorable responses and distinguish direct pharmacological effects from secondary changes resulting from improved metabolic status.

Within the translational framework presented in Figure 4, multi-omics and systems biology form the mechanistic bridge between individual participant characteristics and observed clinical outcomes. For example, integrating microbiome profiles with pharmacokinetic biomarkers may determine whether clinical non-response results from limited glucoraphanin conversion, while combining transcriptomic and metabolomic data may establish whether adequate exposure produces the anticipated molecular response. Linking these measurements to body composition and metabolic outcomes could help determine which biological changes are merely mechanistic signals and which predict clinically meaningful benefit.

Nevertheless, multi-omics approaches also introduce important methodological challenges. They are expensive, computationally demanding, susceptible to false-positive findings, and often limited by small sample sizes relative to the number of measured variables. Differences in sample collection, processing, analytical platforms, bioinformatic pipelines, and statistical methods may further reduce reproducibility across studies. Future investigations should therefore use prospectively defined hypotheses, adequate sample sizes, standardized protocols, external validation cohorts, and transparent data-integration methods. Without such rigor, multi-omics analyses may generate exploratory associations without improving clinical interpretation or intervention design.

### 7.3. Standardization of Interventions and Pharmacokinetic Characterization

A major obstacle to the interpretation of the current clinical literature is the substantial heterogeneity of the interventions described collectively as sulforaphane or broccoli-derived preparations. Existing studies have used fresh broccoli sprouts, broccoli sprout beverages, broccoli powders, glucoraphanin-rich extracts, broccoli seed preparations, purified sulforaphane, and products containing variable amounts of active myrosinase. These interventions are not pharmacologically equivalent because they differ in precursor content, active sulforaphane yield, conversion efficiency, stability, absorption, and systemic exposure.

This heterogeneity makes it difficult to determine whether differences in clinical outcomes reflect genuine differences in biological efficacy or simply variation in formulation quality and bioavailability. A nominal dose of glucoraphanin, for example, cannot be interpreted as equivalent to the same molar amount of preformed sulforaphane because conversion depends on active plant myrosinase, food processing, gastrointestinal conditions, and microbial thioglucosidase activity. Similarly, two glucoraphanin-containing products may generate markedly different systemic sulforaphane exposure despite apparently comparable administered doses. Future studies should therefore avoid treating all broccoli-derived formulations as interchangeable.

Standardization should include detailed reporting of the botanical source, processing method, glucoraphanin and sulforaphane content, myrosinase activity, product stability, administered dose, dosing frequency, storage conditions, and quality-control procedures. Where relevant, investigators should report both the administered precursor dose and the measured or expected amount of bioavailable sulforaphane. Such information is necessary to enable replication, comparison across trials, interpretation of dose–response relationships, and eventual evidence synthesis.

Pharmacokinetic characterization should also become a routine component of clinical intervention studies. Measurements of urinary dithiocarbamates, circulating sulforaphane metabolites, or other validated exposure biomarkers can confirm that participants received and absorbed the biologically active compound. These measurements are particularly important when neutral findings are observed because they can distinguish true biological non-response from inadequate conversion or systemic exposure. Repeated pharmacokinetic sampling may also help characterize interindividual variability and determine whether differences in exposure are associated with microbiome composition, genotype, formulation, or clinical response.

As indicated in Figure 4, formulation standardization and pharmacokinetic verification provide the methodological foundation connecting the administered intervention to molecular and clinical outcomes. Precision nutrition and multi-omics analyses cannot be interpreted reliably when actual sulforaphane exposure is unknown. Conversely, pharmacokinetic data alone cannot establish clinical benefit unless they are linked to validated biological responses and obesity-specific outcomes. Future research should therefore integrate standardized interventions, exposure biomarkers, molecular-response profiling, and clinically meaningful endpoints within the same trial design.

Ultimately, greater standardization will improve reproducibility, facilitate comparison among studies, support the establishment of dose–response relationships, and enable more reliable assessment of efficacy and safety. This integrated strategy is essential for determining whether sulforaphane and broccoli-derived preparations can progress from heterogeneous experimental interventions to reproducible, evidence-based nutritional approaches for obesity-associated metabolic dysfunction.

### 7.4. Combination Strategies

Given the multifactorial pathophysiology of obesity, it is unlikely that sulforaphane or any other single dietary bioactive compound will provide sufficient efficacy as a stand-alone intervention. Its most plausible future role may therefore be as an adjunct within integrated strategies combining lifestyle modification, dietary intervention, and, where clinically indicated, pharmacotherapy. Such combination approaches should not be assumed to be additive or synergistic solely because the interventions influence overlapping biological pathways. They require direct evaluation to determine whether combined treatment improves clinically meaningful outcomes, alters bioavailability or pharmacodynamics, introduces safety concerns, or provides benefits beyond those achieved by established therapies alone.

As illustrated in Figure 4, future combination strategies should integrate standardized sulforaphane exposure with well-defined lifestyle or pharmacological interventions, validated biomarkers of biological response, and obesity-specific clinical endpoints. This framework would enable investigators to distinguish mechanistic complementarity from genuine therapeutic benefit and clarify the position of sulforaphane within comprehensive obesity management.

#### 7.4.1. Sulforaphane Combined with Exercise

Physical activity remains a cornerstone of obesity management because it improves cardiorespiratory fitness, insulin sensitivity, mitochondrial function, inflammatory regulation, energy expenditure, and overall cardiometabolic health, even when body weight loss is modest [139,140]. Several of these physiological adaptations overlap with pathways influenced by sulforaphane, including AMPK-dependent signaling, PGC-1α-mediated mitochondrial biogenesis, oxidative metabolism, insulin signaling, and cellular antioxidant defense [139,141,142]. This mechanistic overlap provides a rationale for investigating combined interventions but does not, by itself, establish that their effects will be additive or synergistic.

A clinically relevant question is whether sulforaphane can enhance specific exercise-induced adaptations, particularly in individuals with obesity-associated mitochondrial dysfunction, insulin resistance, or chronic low-grade inflammation. Future randomized trials should compare exercise alone with exercise plus standardized sulforaphane supplementation and assess outcomes such as body composition, visceral adiposity, insulin sensitivity, cardiorespiratory fitness, skeletal-muscle mitochondrial function, inflammatory status, and adherence. Such studies should also confirm systemic sulforaphane exposure and determine whether baseline metabolic phenotype modifies treatment responsiveness.

Potential interactions with exercise-induced redox signaling require particular attention. Reactive oxygen species generated during exercise contribute to adaptive responses involving mitochondrial biogenesis, endogenous antioxidant defense, and metabolic remodeling. Because sulforaphane activates endogenous cytoprotective pathways rather than functioning solely as a direct antioxidant, its interaction with exercise may differ from that of high-dose antioxidant supplementation. Nevertheless, inappropriate dose, formulation, or timing could theoretically modify redox-sensitive adaptations. Future studies should therefore examine dose–response and timing effects and should not assume that greater antioxidant pathway activation necessarily produces superior exercise adaptation.

Overall, combined sulforaphane and exercise interventions are mechanistically plausible, but their clinical value remains unproven. The principal objective should be to determine whether sulforaphane provides incremental benefit beyond structured exercise rather than merely reproducing molecular effects already induced by physical activity.

#### 7.4.2. Sulforaphane Combined with Dietary Interventions

Dietary modification remains a foundational component of obesity prevention and treatment. Mediterranean, predominantly plant-based, energy-restricted, low-glycemic-index, and high-fiber dietary patterns may improve metabolic health through multiple mechanisms, including reduced energy density, improved nutrient quality, modulation of inflammation and oxidative stress, and favorable effects on the gut microbiota. Sulforaphane-rich foods or standardized broccoli-derived preparations could potentially complement these approaches; however, their added value should be evaluated relative to the effects of the overall dietary pattern.

The dietary context may influence both sulforaphane exposure and biological responsiveness. Food processing, mastication, cooking practices, meal composition, gastrointestinal transit, and the availability of plant or microbial myrosinase can substantially alter glucoraphanin conversion and systemic sulforaphane exposure. In addition, dietary patterns rich in fiber and plant-derived compounds may modify the gut microbiota in ways that affect glucoraphanin metabolism. Therefore, the efficacy of sulforaphane cannot necessarily be considered independently of the diet in which it is consumed.

Future trials should determine whether the addition of sulforaphane-rich foods or standardized supplements provides measurable benefits beyond evidence-based dietary intervention alone. Factorial or multi-arm designs would be particularly useful for separating the effects of the background diet from those of sulforaphane. Outcomes should include body weight, waist circumference, body composition, visceral and ectopic fat, insulin sensitivity, hepatic steatosis, inflammatory markers, microbiome composition, and validated biomarkers of sulforaphane exposure.

The potential contribution of sulforaphane to adherence also warrants careful interpretation. Incorporating broccoli-derived foods into a sustainable dietary pattern may support overall diet quality, whereas supplementation may improve dosing consistency but does not reproduce the nutritional complexity of whole foods. Accordingly, future research should directly compare food-based and supplement-based approaches and evaluate not only efficacy but also feasibility, acceptability, long-term adherence, and cost-effectiveness.

#### 7.4.3. Sulforaphane Combined with Anti-Obesity Pharmacotherapy

The rapidly evolving field of obesity pharmacotherapy provides an important clinical context for evaluating the potential role of sulforaphane and broccoli-derived preparations. GLP-1 receptor agonists such as liraglutide and semaglutide, together with the dual GIP/GLP-1 receptor agonist tirzepatide, have demonstrated substantially greater effects on body weight than those reported for nutritional bioactive compounds, with mean reductions of approximately 10–25% of initial body weight in major randomized trials, depending on the drug, dose, population, and treatment duration [143,144]. These therapies therefore represent a fundamentally different level of evidence and clinical efficacy from that currently available for sulforaphane.

The existing literature does not support sulforaphane as an alternative to established anti-obesity medications. Its potential relevance lies instead in its effects on biological processes associated with obesity-related metabolic dysfunction, including oxidative stress, chronic inflammation, mitochondrial impairment, insulin resistance, hepatic steatosis, and gut microbiota dysbiosis. These mechanisms are not entirely separate from those influenced indirectly by pharmacological weight loss, and it remains uncertain whether adding sulforaphane would provide clinically meaningful benefit once substantial weight reduction and metabolic improvement have already been achieved with medication.

Accordingly, mechanistic complementarity should not be equated with therapeutic additivity. Future trials should determine whether sulforaphane provides incremental improvements in residual metabolic abnormalities, treatment tolerability, body-composition preservation, hepatic outcomes, inflammatory status, or cardiometabolic risk beyond pharmacotherapy alone. Such studies should use standardized sulforaphane formulations, confirm systemic exposure, and include adequately powered comparisons between pharmacotherapy alone and pharmacotherapy plus sulforaphane.

Safety and interaction assessment will be essential. Although sulforaphane is generally regarded as well tolerated at commonly studied exposures, combined use with potent metabolic therapies may influence gastrointestinal tolerability, appetite, nutrient intake, glycemic control, medication adherence, or pharmacodynamic responses. Potential interactions should therefore be investigated rather than presumed absent. Particular attention should be paid to individuals receiving multiple medications or those with diabetes, hepatic disease, or other comorbidities.

Emerging treatments, including triple-receptor agonists, amylin analogues, and combination metabolic therapies, increasingly target multiple components of energy balance and metabolic regulation [145,146]. In this context, the future role of sulforaphane is most plausibly that of a supportive nutritional adjunct rather than a primary weight-loss therapy. Establishing such a role will require evidence of additive clinical benefit, acceptable safety, and practical value beyond contemporary pharmacological and lifestyle care.

#### 7.4.4. Personalized Sulforaphane Supplementation

Personalized sulforaphane supplementation represents the convergence of precision nutrition, pharmacokinetics, microbiome science, pharmacogenomics, and systems biology. Rather than applying a uniform dose to all individuals, future strategies may tailor formulation, dose, timing, and treatment duration according to characteristics that influence glucoraphanin conversion, sulforaphane exposure, and biological responsiveness.

Potential determinants of individualized intervention include glutathione S-transferase and other xenobiotic-metabolizing enzyme polymorphisms, gut microbiota composition, baseline dietary exposure, obesity phenotype, insulin resistance, inflammatory burden, sex, age, medication use, and habitual dietary patterns. Biomarkers such as urinary dithiocarbamates, circulating sulforaphane metabolites, inflammatory mediators, oxidative stress indices, metabolic signatures, and microbiome-derived functional measures may help distinguish inadequate exposure from limited biological responsiveness.

However, the concept of personalized supplementation currently remains largely investigational. No validated clinical algorithm is available to determine which individuals should receive sulforaphane, which formulation should be selected, or what exposure is required for a clinically meaningful response. Biomarker associations identified in exploratory studies must therefore be validated prospectively before being used to guide supplementation.

Future personalized trials should incorporate predefined responder criteria, standardized products, exposure verification, and clinically relevant outcomes. Adaptive or biomarker-stratified designs may help determine whether tailoring supplementation improves efficacy compared with uniform dosing. Cost, accessibility, analytical complexity, and feasibility must also be considered, because a biologically sophisticated strategy will have limited clinical value if it cannot be implemented reproducibly in routine practice.

Within the framework shown in Figure 4, personalized supplementation should be regarded as the endpoint of an integrated evidence pathway rather than an immediate clinical application. Reliable personalization will depend on validated relationships among intervention composition, systemic exposure, molecular response, and clinical benefit.

### 7.5. Concluding Perspective

Future research on sulforaphane and broccoli-derived preparations should progress beyond conventional supplementation studies toward an integrated translational framework. As summarized in Figure 4, this framework should connect standardized formulation and dosing, pharmacokinetic confirmation, microbiome and multi-omics profiling, participant stratification, combination interventions, and clinically meaningful obesity-related outcomes. These components are interdependent: precision nutrition cannot be implemented reliably without validated biomarkers, multi-omics findings cannot be interpreted without adequate exposure characterization, and combination strategies cannot be justified without evidence of benefit beyond established care.

The most immediate priority is not to expand the number of small exploratory studies, but to improve the methodological quality and comparability of future research. Adequately powered randomized controlled trials should use well-characterized formulations, standardized reporting of glucoraphanin and sulforaphane content, validated exposure biomarkers, sufficiently long follow-up, and obesity-specific primary endpoints. Comprehensive body-composition assessment should be incorporated whenever feasible, alongside metabolic, inflammatory, hepatic, and functional outcomes.

Precision nutrition, microbiome-guided interventions, and multi-omics technologies may eventually help explain interindividual variability and identify populations more likely to benefit. However, these approaches should remain hypothesis-generating until predictive biomarkers and responder profiles are independently validated. Similarly, combining sulforaphane with exercise, dietary interventions, or anti-obesity medications is mechanistically plausible, but additive or synergistic effects cannot be assumed and must be demonstrated in appropriately controlled studies. Overall, the future clinical value of sulforaphane will depend less on identifying additional molecular targets and more on establishing reproducible relationships among formulation, systemic exposure, biological response, and meaningful patient outcomes. Until such evidence is available, sulforaphane should be regarded as a promising investigational nutritional adjunct for obesity-associated metabolic dysfunction rather than an established treatment for obesity. A coordinated multidisciplinary research strategy integrating nutrition science, pharmacology, microbiology, molecular biology, bioinformatics, and clinical medicine will be essential to determine whether its mechanistic and preclinical promise can be translated into safe, reproducible, and clinically relevant benefit.

## 8. Conclusions

Obesity is a multifactorial chronic disease characterized by disturbances in energy homeostasis, chronic low-grade inflammation, oxidative stress, mitochondrial dysfunction, and metabolic dysregulation. The increasing global burden of obesity and the limitations of current therapeutic approaches have stimulated growing interest in complementary nutritional strategies capable of targeting multiple pathogenic pathways. Among dietary bioactive compounds, sulforaphane and broccoli-derived preparations have attracted considerable scientific interest because of their pleiotropic biological activities and the generally acceptable short-term tolerability reported in the available clinical studies. However, their therapeutic role in obesity remains to be established.

Substantial mechanistic and preclinical evidence supports the biological plausibility that sulforaphane favorably modulates multiple pathways implicated in obesity-associated metabolic dysfunction. Experimental studies consistently demonstrate activation of the Nrf2 and AMPK signaling pathways, suppression of NF-κB-mediated inflammation, improvement of mitochondrial function, promotion of thermogenesis and adipose tissue browning, regulation of lipid metabolism, attenuation of oxidative stress, and modulation of gut microbiota composition. Consistent findings from in vitro and animal studies further demonstrate beneficial effects on adipogenesis, lipid accumulation, insulin resistance, hepatic steatosis, inflammation, oxidative stress, and energy metabolism, providing strong mechanistic support for continued investigation of sulforaphane as a potential nutritional approach for obesity-associated metabolic dysfunction.

Human clinical evidence, however, remains limited and heterogeneous. Although several studies suggest improvements in glycemic control, insulin sensitivity, endothelial function, oxidative stress, inflammation, and selected liver-related biomarkers, clinically meaningful effects on body weight, body mass index, adiposity, and body composition have not been consistently demonstrated. Furthermore, the reported improvements primarily involve surrogate metabolic biomarkers and should not be interpreted as evidence of reduced obesity-related morbidity, clinically meaningful adiposity reduction, or established anti-obesity efficacy. Therefore, the current clinical evidence is more supportive of potential improvements in obesity-associated metabolic dysfunction than of clinically meaningful reductions in body weight or adiposity.

Important translational challenges also remain. Variability in sulforaphane bioavailability, formulation characteristics, myrosinase activity, gut microbiota composition, host genetic background, and interindividual responsiveness complicates interpretation of the available evidence and limits comparisons across studies. Moreover, most clinical investigations have been designed primarily to evaluate metabolic biomarkers rather than obesity-specific clinical endpoints and are frequently limited by relatively small sample sizes, short intervention durations, methodological heterogeneity, and insufficient pharmacokinetic characterization. Although the available clinical literature indicates generally acceptable short-term tolerability, long-term safety, optimal dosing regimens, standardized exposure, and the safety of prolonged supplementation remain insufficiently characterized.

Overall, the available evidence demonstrates strong mechanistic plausibility and encouraging preclinical efficacy for sulforaphane and broccoli-derived preparations in obesity-associated metabolic dysfunction. However, current human evidence is insufficient to establish their efficacy for clinically meaningful weight reduction, adiposity management, or the treatment of obesity. Accordingly, sulforaphane and broccoli-derived preparations should presently be regarded as promising research candidates for obesity-associated metabolic dysfunction rather than evidence-based nutritional interventions for obesity. Future adequately powered, long-term randomized controlled trials employing standardized formulations, rigorous pharmacokinetic assessments, validated biomarkers of sulforaphane exposure, comprehensive body composition analyses, and clinically relevant obesity-specific endpoints will be essential to determine whether the promising mechanistic and preclinical findings can ultimately be translated into safe, reproducible, and clinically meaningful benefits for individuals living with obesity.

## 9. Methods

### 9.1. Review Design

The present study was conducted as a structured narrative review to provide a comprehensive and critical overview of the available evidence regarding the role of sulforaphane and broccoli-derived preparations in obesity and obesity related metabolic dysfunction. The review integrates findings from basic, translational, and clinical research to evaluate the biological mechanisms, pharmacological actions, preclinical efficacy, clinical evidence, bioavailability, gut microbiota interactions, and translational perspectives of sulforaphane in obesity and obesity-associated metabolic disorders.

The review methodology was developed in accordance with the recommendations of the Scale for the Assessment of Narrative Review Articles (SANRA), a validated instrument designed to improve the methodological quality, transparency, and reporting of narrative reviews [147,148,149]. In accordance with SANRA principles, particular attention was given to clearly defining the rationale and objectives of the review, conducting a comprehensive literature search, critically appraising and interpreting the available evidence, ensuring appropriate referencing, and providing a balanced synthesis of current knowledge while identifying important knowledge gaps and future research priorities [147,148,149].

Unlike systematic reviews, the objective of the present review was not to identify every potentially eligible publication or to perform a formal quantitative synthesis of the evidence, but rather to critically integrate and interpret the available mechanistic, preclinical, and clinical literature relevant to the role of sulforaphane and broccoli-derived preparations in modulating biological pathways involved in obesity-associated metabolic dysfunction [147,148,149].

### 9.2. Literature Search Strategy

A comprehensive literature search was performed using four major electronic databases: PubMed/MEDLINE, Scopus, Web of Science, and Google Scholar. The search included publications from database inception until 30 June 2026. In addition, manual screening of the reference lists of eligible original articles, review papers, and other key publications was performed to identify additional relevant studies.

Although studies published throughout the available literature were considered, particular emphasis was placed on publications from the previous 15–20 years, reflecting the substantial advances that have occurred in obesity and metabolic abnormalities biology, molecular pharmacology, nutrigenomics, and translational nutrition research during this period.

The search strategy combined Medical Subject Headings (MeSH), where applicable, together with free-text keywords related to sulforaphane, broccoli-derived preparations, obesity, and metabolic health. Representative search terms included “sulforaphane”, “glucoraphanin”, “broccoli sprouts”, “broccoli extract”, “broccoli seed extract”, “cruciferous vegetables”, “obesity”, “overweight”, “adipogenesis”, “adipocyte differentiation”, “lipid metabolism”, “thermogenesis”, “body weight”, “metabolic syndrome”, “insulin resistance”, “glucose metabolism”, “oxidative stress”, “inflammation”, “gut microbiota”, “Nrf2”, “AMPK”, “clinical trial”, “animal study”, and “cell culture”. Boolean operators (AND, OR) were used to combine search terms and maximize retrieval of relevant publications. Representative search combinations included (“sulforaphane” OR “glucoraphanin”) AND (“obesity” OR “overweight”), (“broccoli sprouts” OR “broccoli extract”) AND (“adipogenesis” OR “weight loss”), (“sulforaphane”) AND (“insulin resistance” OR “metabolic syndrome”), (“sulforaphane”) AND (“thermogenesis” OR “brown adipose tissue”), and (“cruciferous vegetables”) AND (“obesity” OR “metabolic health”). Only studies published in English were considered.

The search strategy was adapted according to the indexing system and search functionality of each database. In PubMed/MEDLINE, MeSH terms were combined with free-text keywords to maximize retrieval of both indexed and recently published articles. Because Scopus and Web of Science do not use MeSH indexing, keyword-based searches employing Boolean operators were performed primarily within the title, abstract, and author keyword fields. Google Scholar was searched using broader keyword combinations and citation searching to identify additional potentially relevant publications not readily captured by the other databases. These adaptations were intended to optimize literature retrieval while maintaining consistency across the different database platforms.

Only studies published in English were considered. The literature search was designed to ensure broad coverage of the available evidence relevant to the objectives of this narrative review rather than exhaustive identification of all potentially eligible publications, as would be expected in a systematic review [147,148,149].

### 9.3. Eligibility Criteria

Studies were considered eligible if they investigated sulforaphane, glucoraphanin, broccoli-derived preparations, or cruciferous vegetable-derived interventions and evaluated outcomes related to obesity, overweight, adiposity, body weight regulation, metabolic syndrome, insulin resistance, lipid metabolism, inflammation, oxidative stress, thermogenesis, energy expenditure, or other obesity-related metabolic abnormalities.

Eligible evidence included in vitro cellular studies, animal experiments, randomized and non-randomized clinical studies, observational investigations, and relevant review articles, where appropriate, to provide mechanistic, preclinical, and clinical context. Mechanistic investigations elucidating the molecular and cellular actions of sulforaphane relevant to obesity and obesity related metabolic abnormalities were also included. Only articles published in peer-reviewed journals and available in English were considered.

Studies were excluded if they did not investigate sulforaphane, glucoraphanin, or broccoli-derived preparations, focused on unrelated disease areas without reporting obesity-related outcomes, consisted solely of conference abstracts, editorials, letters, commentaries, or unpublished reports lacking sufficient methodological information, or were unavailable in full-text form.

### 9.4. Study Selection and Data Extraction

Publications identified through the literature search were evaluated for their relevance to the objectives and thematic scope of the review. Full-text articles considered directly relevant were examined in detail and incorporated into the evidence synthesis.

Data were extracted manually and included information regarding study design, experimental model or study population, intervention characteristics, dose, duration, route of administration, principal obesity-related outcomes, proposed molecular mechanisms, and the main conclusions of each study. For human investigations, additional information concerning participant characteristics, intervention protocols, metabolic outcomes, and safety findings was extracted where available.

The extracted information was subsequently organized according to major thematic areas, including molecular mechanisms, adipogenesis, lipid metabolism, inflammation, oxidative stress, mitochondrial function, thermogenesis, insulin sensitivity, gut microbiota, bioavailability, pharmacokinetics, animal studies, clinical evidence, and safety considerations, thereby facilitating critical comparison of findings across different experimental models.

### 9.5. Evidence Synthesis

The available literature was synthesized narratively according to study design and thematic relevance. Particular emphasis was placed on integrating evidence derived from mechanistic cellular studies, animal models, and human clinical investigations to provide a comprehensive overview of the potential effects of sulforaphane and broccoli-derived preparations in modulating biological pathways involved in obesity-associated metabolic dysfunction.

Special consideration was given to the biological plausibility of reported mechanisms, consistency of findings across experimental models, translational relevance, methodological limitations of the available evidence, and current knowledge gaps. Rather than providing a formal quantitative assessment of study quality or certainty of evidence, the review critically evaluated the strengths and limitations of the available literature within each thematic section in accordance with the objectives of a structured narrative review and the recommendations of SANRA [147,148,149].

Although relevant review articles were consulted to provide background information, contextualize the current state of knowledge, and identify additional primary studies, the evidence synthesis presented in this review was based primarily on original in vitro, animal, and human investigations.

### 9.6. Methodological Limitations

Several methodological limitations should be considered when interpreting the findings of this review. Although a structured and comprehensive literature search was conducted following SANRA recommendations [147,148,149], the present work was designed as a narrative review rather than a systematic review. Consequently, the literature identification and study selection processes were intended to provide comprehensive coverage of the current evidence rather than exhaustive retrieval of every potentially eligible publication. Accordingly, a PRISMA-style study-selection flow diagram, numerical accounting of records retrieved from individual databases, and quantitative reporting of full-text exclusions were not generated because these procedures are characteristic of systematic reviews and would not accurately reflect the iterative literature identification and selection process employed in the present narrative review.

Furthermore, the available literature is characterized by considerable heterogeneity with respect to experimental models, intervention formulations, sulforaphane dosage, treatment duration, outcome measures, and methodological approaches, limiting direct comparison among studies. Much of the current evidence originates from mechanistic in vitro investigations and animal studies, whereas human clinical evidence remains comparatively limited and is frequently constrained by relatively small sample sizes, short intervention periods, heterogeneous formulations, and substantial variability in sulforaphane bioavailability.

Only English-language publications were included in this review. Consequently, language bias cannot be excluded, and potentially relevant studies published in other languages may not have been identified. This restriction may have influenced the completeness of the available evidence and should therefore be considered when interpreting the findings of the present review.

As with all narrative reviews, publication bias and selective reporting cannot be completely excluded [150,151]. Consequently, the conclusions presented herein should be interpreted within the context of these methodological limitations. Additional well-designed randomized controlled trials employing standardized sulforaphane formulations, clinically relevant obesity-related endpoints, and comprehensive pharmacokinetic and body composition assessments are required to further clarify the therapeutic potential of sulforaphane and broccoli-derived preparations in modulating biological pathways involved in obesity-associated metabolic dysfunction.

## Figures and Tables

**Table 1:** In vitro cellular studies investigating the effects of sulforaphane and broccoli-derived preparations in models of obesity and obesity-related metabolic dysfunction.

Study	Cellular Model	Evidence Type *	Sulforaphane/Preparation Dose	Main Findings	Proposed Mechanisms
Choi et al. [21]	Murine 3T3-L1 preadipocytes	Direct obesity-related evidence	5–20 μM SFN	Inhibited adipocyte differentiation and lipid accumulation	Inhibition of mitotic clonal expansion; G0/G1 cell-cycle arrest; ↓ cyclin D1, cyclin A, CDK2, CDK4, p-Rb; ↑ p21/p27; AMPK activation; suppression of Akt and ERK signaling; ↓ PPARγ and C/EBPα
Lee et al. [22]	BPA-treated murine 3T3-L1 preadipocytes	Direct obesity-related evidence	20 μM SFN	Attenuated BPA-induced adipogenesis and triglyceride accumulation	Cell-cycle arrest; AMPK activation; inhibition of Akt and ERK1/2 signaling; suppression of PPARγ and C/EBPα
Valli et al. [23]	Human adipocytes and murine 3T3-L1 cells	Direct obesity-related evidence	10–50 μM SFN	Reduced adipocyte differentiation and adipogenic gene expression	Downregulation of pro-adipogenic genes; inhibition of adipocyte maturation and lipid accumulation
Li et al. [24]	Mammary adipose mesenchymal stem cells	Direct obesity-related evidence	10 μM SFN	Suppressed adipogenic differentiation	Reduced adipogenic commitment; inhibition of adipocyte maturation; suppression of adipogenic transcriptional program
Sakuma et al. [25]	Murine 3T3-L1 preadipocytes	Direct obesity-related evidence	2–10 μM SFN	Inhibited adipogenesis and lipid accumulation	Downregulation of PPARγ and C/EBPα; inhibition of adipocyte differentiation and triglyceride accumulation
Masuda et al. [26]	Differentiated murine 3T3-L1 adipocytes	Direct obesity-related evidence	10 μM SFN	Reduced lipid accumulation; stimulated lipophagy	Activation of AMPK–mTOR–ULK1 pathway; induction of autophagy/lipophagy; enhanced lipid droplet degradation
Zhang et al. [27]	Murine 3T3-L1 adipocytes	Direct obesity-related evidence	0.2–10 μM SFN	Induced adipocyte browning; increased glucose and lipid utilization	↑ UCP1, PGC-1α and thermogenic genes; AMPK activation; Nrf2 activation; enhanced mitochondrial biogenesis
Liu et al. [28]	Differentiated murine C3H10T1/2 adipocytes (beige/browning adipocyte model)	Direct obesity-related evidence	1–20 μM SFN	Promoted adipocyte browning and thermogenic activity	Upregulation of UCP1, PGC-1α and PRDM16; enhanced mitochondrial biogenesis and fatty acid oxidation
Williams et al. [29]	Palmitic acid-stimulated human THP-1 monocytes and adipose tissue macrophages	Supportive mechanistic evidence	1–10 μM SFN	Reduced inflammatory cytokine production and inflammasome activation	Inhibition of NF-κB signaling; suppression of TNF-α, IL-6, IL-1β; inhibition of NLRP3 inflammasome; promotion of anti-inflammatory phenotype
Lei et al. [30]	Human HepG2 hepatocytes and murine AML12 hepatocytes with lipid-overload-induced steatosis	Supportive mechanistic evidence	10 μM SFN	Enhanced mitochondrial function and fatty acid oxidation	↑ PGC-1α, NRF1, TFAM; increased mitochondrial biogenesis, respiratory function and ATP production
Yang et al. [31]	Murine C2C12 skeletal muscle myotubes	Supportive mechanistic evidence	10 μM SFN	Enhanced mitochondrial biogenesis and oxidative metabolism	HDAC8 inhibition; activation of PGC-1α signaling; increased mitochondrial content and oxidative capacity
Teng et al. [32]	Palmitate-induced insulin-resistant human HepG2 hepatocytes	Supportive mechanistic evidence	1–20 μM SFN	Improved insulin sensitivity	Inhibition of serine palmitoyltransferase 3 (SPTLC3); reduced ceramide biosynthesis; restoration of Akt-mediated insulin signaling
Li et al. [33]	Palmitic acid-induced human HepG2 hepatocytes (NAFLD model)	Supportive mechanistic evidence	1–10 μM SFN	Reduced lipid accumulation and apoptosis	AMPK activation; Nrf2 activation; suppression of SREBP-1c, FASN, DGAT1 and PPARγ; reduced ROS, ceramide accumulation and apoptosis
Hong et al. [34]	Bisphenol A-treated LO2 human hepatocytes	Supportive mechanistic evidence	0.25–1 μM SFN	Reduced hepatic lipid accumulation	Inhibition of ER stress; suppression of CHOP and lipogenic pathways; improvement of lipid homeostasis
Tian et al. [35]	Human HepG2 and LO2 human steatotic hepatocytes	Supportive mechanistic evidence	1–20 μM SFN	Improved lipid metabolism and reduced lipogenesis	Inhibition of ER stress-dependent XBP1/ACC/SCD1 pathway and ER stress-independent SREBP/FASN signaling
Lei et al. [36]	Oleic acid/palmitic acid-induced human HepG2 hepatocytes	Supportive mechanistic evidence	10 μM SFN	Reduced steatosis and lipid accumulation	Induction of lipophagy; activation of autophagic flux; enhanced lipid droplet degradation
Miyata et al. [37]	Human hepatoma Huh-7 cells	Supportive mechanistic evidence	30–100 μM SFN	Suppressed lipogenic gene expression	Accelerated degradation of SREBP precursors; inhibition of SREBP transcriptional activity
Ranaweera et al. [38]	Murine 3T3-L1 preadipocytes	Direct obesity-related evidence	Broccoli leaf extract: 20–200 μg/mL	Reduced adipogenesis and lipid accumulation	Downregulation of PPARγ and C/EBPα; suppression of adipogenic gene expression; anti-adipogenic activity attributed largely to sulforaphane
Men et al. [39]	Murine 3T3-L1 preadipocytes	Direct obesity-related evidence	SFN broccoli sprout + mustard seed preparations (162.29 ± 1.24 µmol/g SFN)	Anti-adipogenic and anti-obesogenic activity	Suppression of adipogenic transcription factors; reduced lipid accumulation; enhanced lipid utilization
Men et al. [40]	Murine 3T3-L1 preadipocytes	Direct obesity-related evidence	SFN broccoli sprout + mustard seed preparations (162.29 ± 1.24 µmol/g SFN)	Reduced triglyceride accumulation and adipocyte differentiation	↓ PPARγ and ↓ C/EBPα; inhibition of adipocyte maturation and lipid storage
Zhou et al. [41]	CYP2E1-overexpressing HepG2 human hepatocytes	Supportive mechanistic evidence	6 μM SFN	Reduced hepatic steatosis and oxidative stress	Nrf2 activation; suppression of CYP2E1-mediated oxidative injury; reduced lipid accumulation
Tubbs et al. [42]	INS-1 pancreatic β-cells and primary mouse hepatocytes	Supportive mechanistic evidence	3 μM SFN	Improved ER–mitochondria communication and glucose metabolism	Restoration of ER–mitochondria interactions; reduced ER stress; suppression of hepatic glucose production
He et al. [43]	Immortalized human hepatocytes (L02 cells)	Supportive mechanistic evidence	1–20 μM SFN	Attenuated homocysteine-induced ER stress	Nrf2 activation; induction of antioxidant enzymes; ↓ GRP78, PERK and CHOP signaling
Li et al. [44]	Rat H9c2 cardiomyocytes subjected to hypoxia/reoxygenation	Supportive mechanistic evidence	0.1–5 μM SFN	Reduced ER stress and apoptosis	SIRT1 activation; ↓ GRP78, CHOP, caspase-12; improved mitochondrial membrane potential
Zhang et al. [45]	Palmitate-induced insulin-resistant human HepG2 hepatocytes	Supportive mechanistic evidence	5 nM SFN	Improved insulin sensitivity and redox homeostasis	AMPK/Nrf2/GPx4 activation; reduced oxidative stress; restoration of insulin signaling
Li et al. [46]	Retinal pigment epithelial cells and diabetic retinal models	Supportive mechanistic evidence	2.5 μM SFN	Suppressed NLRP3 inflammasome activation	Nrf2 activation; inhibition of NLRP3-mediated inflammatory signaling

Abbreviations: SFN, sulforaphane; ACC, acetyl-CoA carboxylase; Akt, protein kinase B; AMPK, AMP-activated protein kinase; BPA, bisphenol A; C/EBPα, CCAAT/enhancer-binding protein α; CDK, cyclin-dependent kinase; CHOP, C/EBP homologous protein; CYP2E1, cytochrome P450 2E1; DGAT1, diacylglycerol acyltransferase 1; ER, endoplasmic reticulum; ERK1/2, extracellular signal-regulated kinases 1/2; FAS, fatty acid synthase; GPx4, glutathione peroxidase 4; GRP78, glucose-regulated protein 78; HDAC8, histone deacetylase 8; IL, interleukin; mTOR, mechanistic target of rapamycin; MASLD, metabolic dysfunction-associated steatotic liver disease; NF-κB, nuclear factor kappa B; NLRP3, NLR family pyrin domain containing 3; NRF1, nuclear respiratory factor 1; Nrf2, nuclear factor erythroid 2-related factor 2; PERK, protein kinase RNA-like endoplasmic reticulum kinase; PGC-1α, peroxisome proliferator-activated receptor gamma coactivator-1α; PPARγ, peroxisome proliferator-activated receptor γ; PRDM16, PR domain containing 16; ROS, reactive oxygen species; SCD1, stearoyl-CoA desaturase 1; SIRT1, sirtuin 1; SPTLC3, serine palmitoyltransferase long chain base subunit 3; SREBP, sterol regulatory element-binding protein; TFAM, mitochondrial transcription factor A; THP-1, human acute monocytic leukemia cell line; TNF-α, tumor necrosis factor-α; UCP1, uncoupling protein 1; ULK1, unc-51-like kinase 1; XBP1, X-box binding protein 1; FABP4: fatty acid-binding protein 4; ACOX1: Acyl-CoA Oxidase 1. * Evidence Type: Direct obesity-related evidence refers to studies directly investigating obesity-associated cellular processes, including adipogenesis, adipocyte differentiation, lipid accumulation, thermogenesis, and adipocyte browning. Supportive mechanistic evidence refers to studies investigating obesity-associated metabolic abnormalities, including insulin resistance, hepatic steatosis, inflammation, oxidative stress, mitochondrial dysfunction, endoplasmic reticulum stress, and glucose metabolism.

**Table 2:** In vivo studies investigating the effects of sulforaphane and broccoli-derived preparations in animal models of obesity and obesity-related metabolic dysfunction.

Study	Animal Model	Evidence Type *	Intervention	Main Findings	Proposed Mechanisms
Liu et al. [28]	HFD-fed obese C57BL/6 mice	Direct obesity-related evidence	10 mg SFN/kg b.w. daily, 30 days (intraperitoneal injection)	Increased energy expenditure and reduced fat mass, adipocyte size, insulin resistance	WAT browning; UCP1/PGC-1α-mediated thermogenesis; AMPK activation
Yang et al. [31]	HFD-fed obese C57BL/6J mice	Direct obesity-related evidence	50 mg SFN/kg b.w. daily, 15 weeks (oral)	Reduced obesity and insulin resistance	Skeletal-muscle mitochondrial biogenesis via HDAC8–PGC1α
Li et al. [33]	HFD-fed MASLD C57BL/6J mice	Supportive mechanistic evidence	10 mg SFN/kg b.w. daily, 3 weeks (intraperitoneal injection)	Reduced hepatic steatosis and apoptosis	Suppression of lipogenesis; enhanced β-oxidation
Hong et al. [34]	Bisphenol A-induced hepatic lipid accumulation in C57/BL6J mice	Supportive mechanistic evidence	10 mg SFN/kg b.w. daily, 4 weeks (intraperitoneal injection)	Reduced hepatic lipid accumulation	Inhibition of ER stress
Tian et al. [35]	HFD-fed MASLD Wistar rats	Supportive mechanistic evidence	5, 10, and 20 mg SFN/kg b.w, 3 times a week, 10 weeks (oral)	Improved lipid metabolism and hepatic steatosis	ER stress modulation; XBP1/ACC/SCD1 and SREBP/FAS regulation
Lei et al. [36]	HFD-fed obese Wistar rats	Supportive mechanistic evidence	20 mg SFN/kg b.w. three times a week, 10 weeks (oral)	Improved hepatic lipid metabolism	Lipophagy activation; AMPK–mTOR signaling
Ranaweera et al. [38]	Leptin-deficient *ob/ob* mice (C57BL/6JHamSlc-ob)	Direct obesity-related evidence	0.5 mg SFN/kg b.w. daily, 6 weeks (oral) 50 and 500 mg broccoli leaf extract /kg b.w. daily, 6 weeks (oral)	Reduced obesity and improved lipid/glucose metabolism	AMPK activation; improved energy homeostasis
Men et al. [39]	Bisphenol A-induced obese C57BL/6J mice	Direct obesity-related evidence	150 mg sulforaphane-rich broccoli sprout powder with or without mustard seed powder/kg b.w. daily, 12 weeks (oral)	Reduced obesity and adiposity	Thermogenesis; anti-adipogenic effects; AMPK activation
Zhou et al. [41]	SV129 humanized CYP2E1 knockin mice	Supportive mechanistic evidence	0.05 g SFN/kg b.w. daily, 5 days (oral)	Reduced hepatic steatosis and oxidative injury	Nrf2 activation; antioxidant defense
Tubbs et al. [42]	HFD & high-sucrose diet diabetic *ob/ob* C57BL/6 mice	Supportive mechanistic evidence	10 mg SFN/kg b.w. daily, 4 weeks (oral)	Reduced hepatic glucose production	Improved ER–mitochondria interactions
Zhang et al. [45]	HFD-fed ICR mice	Direct obesity-related evidence	0.5 mg SFN/kg b.w. five times a week, 8 weeks (subcutaneous injection)	Reduced body weight, improved insulin sensitivity and metabolic dysfunction	AMPK/Nrf2/GPx4 activation; reduced oxidative stress
Li et al. [46]	STZ-diabetic Sprague Dawley rats	Supportive mechanistic evidence	0.5 and 1 mg SFN/kg b.w. daily, 12 weeks (oral)	Reduced oxidative stress and inflammation	Nrf2 activation; NLRP3 inhibition
Yang et al. [59]	HFD-induced MASLD C57BL/6 mice	Supportive mechanistic evidence	30 mg SFN/kg b.w. daily, 9 weeks (oral)	Reduced hepatic inflammation and steatosis	NLRP3 inflammasome suppression
Lee et al. [64]	HFD-fed obese Sprague-Dawley rats	Direct obesity-related evidence	200 and 400 Broccoli sprouts mg/kg b.w. daily, 4 weeks (oral)	Reduced body weight gain and improved lipid profile	Broccoli-derived SFN precursors; lipid-lowering effects
de Souza et al. [65]	STZ-diabetic Wistar rats	Supportive mechanistic evidence	0.1, 0.25, or 0.5 mg SFN/kg b.w. daily, 21 days (oral)	Improved insulin sensitivity, hepatic glycogen, lipid metabolism	Improved glucose homeostasis and antioxidant defenses
Choi et al. [66]	HFD-fed obese C57BL/6N mice	Direct obesity-related evidence	Diet including 1 g SFN/kg daily, 6 weeks (oral)	Reduced body weight gain, adiposity, adipocyte hypertrophy, hepatic steatosis	AMPK activation; inhibition of adipogenesis and lipogenesis
Shawky et al. [67]	High fructose-fed obese Sprague-Dawley rats	Direct obesity-related evidence	0.5 mg SFN/kg b.w. daily, 44 days (oral)	Improved insulin resistance, dyslipidemia, hepatosteatosis	Antioxidant and anti-inflammatory actions
Xu et al. [68]	HFD-fed obese C57BL/6J mice	Supportive mechanistic evidence	18.77 freeze-dried broccoli powder g/kg b.w. daily and 150 μmol glucoraphanin/kg b.w. daily (2.64 g broccoli seed extract powder/kg b.w., 8 weeks (oral)	Improved lipid levels and gut microbiota	Gut microbiota modulation
Jun et al. [69]	Young and aged C57BL/6 mice	Supportive mechanistic evidence	Diet including 442.5 mg SFN/kg daily, 8 weeks (oral)	Altered microbiome and metabolome	Microbiome–metabolome remodeling
Tian et al. [70]	HFD-fed obese C57BL/6J mice	Direct obesity-related evidence	10 mg SFN/kg b.w. daily, 8 weeks (oral)	Improved glucose and lipid metabolism	JNK inhibition; insulin and FGF21 pathway activation
Ashmawy et al. [71]	HFD-fed obese Wistar rats	Direct obesity-related evidence	0.5 or 1 mg SFN/kg b.w. daily, 6 weeks (oral)	Reduced obesity-related metabolic dysfunction	JAK2/STAT3/SOCS3 modulation; leptin signaling
Mansour et al. [72]	HFD-fed MASLD Wistar rats	Supportive mechanistic evidence	10 mg SFN/kg b.w. daily, 4 weeks (oral)	Improved liver pathology and metabolic status	AMPK-mediated attenuation of ER stress
Çakır et al. [73]	HFD-fed obese wild-type, C57BL/6J mice and Lep^ob/ob^ and Lepr^db/db^ mice	Direct obesity-related evidence	5 or 10mg SFN/kg b.w. daily, 2, 3 or 4 weeks (intraperitoneal injection)	Reduced body weight and adiposity	Reversal of leptin resistance
Martins et al. [74]	HFD-fed obese C57BL/6J mice	Direct obesity-related evidence	0.25 or 0.5 SFN mg/kg b.w. daily, 4 weeks (oral)	Reduced adipocyte size and obesity-related alterations	Thermogenic responses and metabolic remodeling
Xu et al. [75]	HFD/high-fructose-fed MASLD C57BL/6 mice	Supportive mechanistic evidence	15 and 30mg SFN/kg b.w. daily, 12 weeks (oral)	Improved MASLD and insulin resistance	Gut–liver axis modulation; LPS/TLR4 suppression
Bankole et al. [76]	HFD-fed obese wild-type C57BL/6J mice	Supportive mechanistic evidence	Diet including 1% broccoli seed extracts with 0.13% glucoraphanin and 0.322% Mustard Powder with myrosinase, 20 weeks (oral)	Improved hepatic steatosis and metabolic dysfunction	Gut microbiota and host metabolome modulation
Zhang et al. [77]	HFD-fed obese C57BL/6J mice	Direct obesity-related evidence	10 mg SFN/kg b.w. daily, 5 times a week, 4 weeks (subcutaneous injection)	Reduced adipose tissue fibrosis and inflammation	M2 macrophage polarization
Huang et al. [78]	High-fat and high-fructose diet-fed MASLD C57BL/6J mice	Supportive mechanistic evidence	15 and 30 mg SFN/kg b.w. every 2 days, 12 weeks (oral)	Improved hepatic inflammation and steatohepatitis	KLF4-mediated M2 macrophage polarization
Luo et al. [79]	Young and middle-aged HFD-fed obese C57BL/6J mice	Direct obesity-related evidence	Prevention study: Diet including 0.5 g SFN/kg, 18 weeks (oral) or Diet including 0.25 g SFN/kg, 7 weeks (oral) Treatment study: 40 mg SFN/kg b.w. daily, 30 days (oral)	Reduced adiposity and insulin resistance	Improved oxidative metabolism, mitochondrial function, metabolic flexibility
Jeon et al. [80]	HFD-fed obese C57BL/6 mice	Direct obesity-related evidence	40–800 mg Broccoli seed hydrolysate/kg b.w. or 400 mg Broccoli seed water extract/kg b.w. or 1.0 and 10 mg SFN/kg 8 b.w. daily, 8 weeks (oral)	Reduced obesity, steatosis, dyslipidemia	5-HT2A/AMPK signaling
Çubuk et al. [81]	High-glycemic-index diet (HGID)-fed C57BL/6 mice	Direct obesity-related evidence	5 mg SFN/kg b.w. daily, 15 weeks (oral) or 5 and 20 mg SFN/kg b.w. daily, 5 weeks (oral) after HGID for 15 weeks	Prevented/treated obesity-related metabolic abnormalities	Improved glycemic control and insulin sensitivity
Kumosani et al. [82]	High-fat/high-fructose-fed Wistar rats	Supportive mechanistic evidence	10 mg SFN/kg b.w. daily, 12 weeks (oral)	Improved oxidative stress, inflammation, and obesity hormone abnormalities	Reduced inflammatory and oxidative stress responses
El-Shahat et al. [83]	High-cholesterol + high-fructose diet-fed Sprague Dawley rats	Supportive mechanistic evidence	0.5 mg SFN/kg b.w. daily, 4 weeks	Alleviated oxidative damage, improved antioxidant status, enhanced glycemic status	Decreased MDA and NO, reduced glutathione, and decreased caspase 9 concentration
Nagata et al. [84]	HFD-fed obese C57BL/6J mice	Direct obesity-related evidence	Diet containing 0.3% glucoraphanin for 14 weeks (oral)	Reduced body weight gain, adiposity, insulin resistance, hepatic steatosis, and metabolic endotoxemia; improved glucose tolerance	Gut microbiota modulation; adipose tissue browning; increased energy expenditure; reduced plasma LPS (metabolic endotoxemia); enhanced intestinal barrier integrity; activation of Nrf2-dependent antioxidant pathways

Abbreviations: SFN, sulforaphane; Nrf2, nuclear factor erythroid 2-related factor 2; HFD, high fat-diet; STZ, streptozocin; AMPK, AMP-activated protein kinase; WAT, white adipose tissue; UCP1, uncoupling protein 1; PGC-1α, peroxisome proliferator-activated receptor gamma coactivator-1α; MASLD, metabolic dysfunction-associated steatotic liver disease; ACC, acetyl-CoA carboxylase; XBP1, X-box binding protein 1; SCD1, stearoyl-CoA desaturase 1; ER, endoplasmic reticulum; SREBP, sterol regulatory element-binding protein; FAS, fatty acid synthase; NLRP3, NLR family pyrin domain containing 3; JNK, c-Jun N-terminal kinase; FGF21, fibroblast growth factor 21; JAK2, janus kinase 2; STAT3, signal transducer and activator of transcription 3; SOCS3, suppressor of cytokine signaling 3; mTOR, mammalian target of rapamycin; GPx4, Glutathione Peroxidase 4; LPS, lipopolysaccharide; TLR4, toll-like receptor 4; HDAC, histone deacetylase; KLF4, Krüppel-like factor 4; 5-HT2A, 5-hydroxytryptamine receptor 2A; MDA, malondialdehyde. * Evidence Type: Direct obesity-related evidence refers to studies evaluating obesity-related phenotypes, including body weight, adiposity, adipocyte morphology, energy expenditure, thermogenesis, and metabolic outcomes directly linked to obesity. Supportive mechanistic evidence refers to studies investigating obesity-associated metabolic abnormalities, including MASLD, insulin resistance, glucose dysregulation, oxidative stress, inflammation, mitochondrial dysfunction, gut microbiota alterations, and other organ-specific complications that provide mechanistic insight into obesity pathophysiology. This makes the rationale for including all studies explicit and fully addresses the reviewer’s concern.

**Table 3:** Summary of clinical studies investigating the effects of sulforaphane and broccoli-derived preparations: direct obesity-related evidence and supportive mechanistic evidence.

Study	Study Design	Evidence Type *	Population	Extract Type or Plant Part	Intervention (Dose and Duration)	Main Findings
Axelsson et al. [94]	RCT with two parallel arms	Supportive mechanistic clinical evidence	97 patients with type 2 diabetes (subgroup analyses in obese individuals with poorly controlled diabetes)	SFN-containing broccoli sprout extracts (150µmol SFN)	Once daily, 12 weeks	Improved fasting glucose and HbA1c, particularly in obese patients, but body weight was essentially unchanged. Benefits occurred independently of weight loss.
Bahadoran et al. [95]	Double-blind, placebo-controlled RCT	Supportive mechanistic clinical evidence	66 patients with type 2 diabetes	Broccoli sprout powder (containing 22.5µmol SFN/g)	5 or 10 g/day, 4 weeks	Reduced oxidative stress markers; increased total antioxidant capacity; decreased oxidized LDL; no significant weight-loss effect was reported
Bahadoran et al. [96]	Double-blind, placebo-controlled RCT	Supportive mechanistic clinical evidence	63 patients with type 2 diabetes	Broccoli sprout powder (containing 22.5µmol SFN/g)	5 or 10 g/day, 4 weeks	Improved insulin resistance indices; reductions in serum insulin and HOMA-IR; no significant weight-loss effect was reported
Bahadoran et al. [97]	Double-blind, placebo-controlled RCT	Supportive mechanistic clinical evidence	72 patients with type 2 diabetes	Broccoli sprout powder (containing 22.5µmol SFN/g)	5 or 10 g/day, 4 weeks	Reduced serum triglycerides; improved oxidized LDL/LDL-C ratio; favorable effects on lipid profile; no significant weight-loss effect was reported
Mirmiran et al. [98]	Double-blind, placebo-controlled RCT	Supportive mechanistic clinical evidence	63 patients with type 2 diabetes	Broccoli sprout powder (containing 22.5µmol SFN/g)	5 or 10 g/day, 4 weeks	Reduced inflammatory biomarkers including hs-CRP and IL-6; attenuation of systemic inflammation; no significant weight-loss effect was reported
Dwibedi et al. [99]	Double-blind, placebo-controlled RCT	Direct obesity-related clinical evidence	74 overweight/obese individuals with prediabetes	Broccoli sprout extract product (containing 150 µmol SFN)	Once broccoli sprout product daily, 12 weeks	Reduced fasting blood glucose in a subgroup of individuals with specific baseline gut microbiota signatures; responders exhibited improved glycemic control; no significant body-weight reduction as a primary finding
van Steenwijk et al. [100]	Double-blind, placebo-controlled crossover RCT	Direct obesity-related clinical evidence	12 healthy overweight adults exposed to a controlled hypercaloric dietary challenge	Whole broccoli sprouts	A single dose of 16 g, blood measurement at 90 and 120 min after intervention	Attenuated calorie-induced inflammatory responses; reduced postprandial inflammatory signaling; improved inflammatory responses to overfeeding; body weight was not significantly affected
Kikuchi et al. [101]	Double-blind, placebo-controlled RCT	Supportive mechanistic clinical evidence	55 adults with fatty liver	Broccoli sprout capsules containing glucoraphanin (approximately 10mg glucoraphanin per capsule)	3 broccoli sprout capsules daily, 8 weeks	Improved blood ALT, γ-GTP levels; prevention of NDMA-induced chronic liver failure. No clinically meaningful weight loss
Satomi et al. [102]	Double-blind, placebo-controlled RCT	Supportive mechanistic clinical evidence	70 healthy overweight adults with elevated liver biomarkers	Broccoli sprout capsules enriched in glucoraphanin (137.1 μmol glucoraphanin per 6 capsules)	6 capsules daily, 24 weeks	Improved liver biomarkers; no meaningful body-weight reduction
López-Chillón et al. [103]	Controlled clinical trial	Direct obesity-related clinical evidence	40 healthy overweight adults	Broccoli sprouts product (51.08mg glucoraphanin/30g broccoli sprouts)	30g daily, 10 weeks	Reduced inflammatory markers IL-6 and CRP. No clinically meaningful weight loss

Abbreviations: SFN, sulforaphane; RCT, randomized controlled trial; HbA1c, hemoglobin A1c; LDL, low-density lipoprotein; HOMA-IR, homeostatic model assessment for insulin resistance; CRP, C-reactive protein; IL-6, interleukin-6; ALT, alanine aminotransferase; γ-GTP, gamma-glutamyl transferase. * Evidence Type: Direct obesity-related clinical evidence refers to studies conducted in overweight or obese individuals that included obesity-related clinical outcomes (e.g., body weight, adiposity, body composition, or anthropometric measures), irrespective of whether significant weight loss was achieved. Supportive mechanistic clinical evidence refers to studies primarily evaluating obesity-associated metabolic abnormalities, including glycemic control, insulin resistance, inflammation, oxidative stress, endothelial function, hepatic function, and lipid metabolism, rather than obesity-related endpoints.

## Data Availability

No new data were created or analyzed in this study. Data sharing is not applicable to this article.
